# Design, computational studies, synthesis and in vitro antimicrobial evaluation of benzimidazole based thio-oxadiazole and thio-thiadiazole analogues

**DOI:** 10.1186/s13065-021-00785-8

**Published:** 2021-10-28

**Authors:** Nada A. Noureldin, Jennifer Richards, Hend Kothayer, Mohammed M. Baraka, Sobhy M. Eladl, Mandy Wootton, Claire Simons

**Affiliations:** 1 School of Pharmacy and Pharmaceutical Sciences, Cardiff University, Cardiff, CF10 3NB UK; 2 Department of Medicinal Chemistry, Faculty of Pharmacy, Zagazig University, Zagazig, P.C. 44519 Egypt; 3 Specialist Antimicrobial Chemotherapy Unit, University Hospital of Wales, Heath Park, Cardiff, CF14 4XW UK

**Keywords:** Benzimidazole, Antimicrobial resistance, PheRS, Phe-AMP, Flexible alignment, Molecular dynamics

## Abstract

**Background:**

Two series of benzimidazole based thio-oxadiazole and thio-thiadiazole analogues were designed and synthesised as novel antimicrobial drugs through inhibition of phenylalanyl-tRNA synthetase (PheRS), which is a promising antimicrobial target. Compounds were designed to mimic the structural features of phenylalanyl adenylate (Phe-AMP) the PheRS natural substrate.

**Methods:**

A 3D conformational alignment for the designed compounds and the PheRS natural substrate revealed a high level of conformational similarity, and a molecular docking study indicated the ability of the designed compounds to occupy both Phe-AMP binding pockets. A molecular dynamics (MD) simulation comparative study was performed to understand the binding interactions with PheRS from different bacterial microorganisms. The synthetic pathway of the designed compounds proceeded in five steps starting from benzimidazole. The fourteen synthesised compounds **5a**-**d**, **6a**-**c**, **8a**-**d** and **9a**-**c** were purified, fully characterised and obtained in high yield.

**Results:**

In vitro antimicrobial evaluation against five bacterial strains showed a moderate activity of compound **8b** with MIC value of 32 μg/mL against *S. aureus*, while all the synthesised compounds showed weak activity against both *E. faecalis* and *P. aeruginosa* (MIC 128 μg/mL).

**Conclusion:**

Compound **8b** provides a lead compound for further structural development to obtain high affinity PheRS inhibitors.

**Supplementary Information:**

The online version contains supplementary material available at 10.1186/s13065-021-00785-8.

## Introduction

Antimicrobial resistance is one of the most challenging medical dilemmas worldwide [1–4]. Antibacterial resistance annually leads to 700,000 deaths, and this number is estimated to reach 10 million annual deaths by 2050 [3]. The emergence of pan-drug resistant bacterial strains that are resistant to all known antimicrobial agents, have been reported [5], which is alarming and could result in a return to the ‘post-antibiotic era’ where a minor injury could be a life-threatening event [6]. Bacterial cells have exceptional survival genetic plasticity mechanisms that are highly flexible in encountering environmental threats [7], therefore efforts should be focused on decreasing antimicrobial resistance development. Improper and over prescription of broad-spectrum antibiotics are the main causes that exert a high pressure enhancing the natural genetic mutations in pathogenic bacteria [1]. Finding new targets within the bacterial cells is considered a promising strategy to overcome the high rates of antimicrobial resistance development.

Aminoacyl tRNA synthetases (aaRSs) are a group of enzymes that encode the aminoacylation of the tRNA in the protein synthesis process [8]. These enzymes are vital for the cell function and their inhibition leads to a cascade of events named ‘the stringent response’ resulting in cell growth cessation [9]. The availability of the resolved crystal structures of most of the aaRS enzymes together with their different structures in bacterial cells compared with their human counterparts, make them a hopeful antibacterial target [10–16].

The aaRS family consists of 23 enzymes, which are classified into two main classes (class I and class II) according to their active site structure and the enzyme kinetics [17]. Class I enzymes usually ligate the bulky amino acids [18], and the active site consists of two motifs: KMSKS (Lys-Met-Ser-Lys-Ser) and HIGH (His-Ile-Gly-His). Class I enzymes usually aminoacylate tRNA at the 2´ hydroxy group of the ribose sugar at the tRNA CCA 3´ end [18–22]. Class II aaRS enzymes are characterised by the presence of three active motifs in their active sites assigned motif I, II and III, and they have either dimeric or tetrameric structures. Class II members charge their cognate amino acids at the 3´ hydroxy group of the A76 ribose at the 3´ end of the tRNA [18–22]. The tRNA aminoacylation reaction takes place in two main steps. Firstly, the amino acid is activated by interaction with ATP, and the product of this reaction is the highly reactive aminoacyl adenylate (aa-AMP) intermediate. Secondly, the esterification reaction between the carboxylic group of the amino acid and either the 2´ or 3´ hydroxy group of the terminal adenine ribose sugar of the tRNA^aa^ occurs resulting in the aminoacyl charged tRNA (aa-tRNA^aa^) [18–22].

Among class II aaRS enzymes is the uniquely structured phenylalanyl-tRNA synthetase (PheRS). Bacterial PheRS is a heterodimer (αβ)_2_ with two small α subunits (PheS) and two large β subunits (PheT), and the aminoacylation active site is located in the α subunit. Although PheRS is a member of class II enzymes, it exclusively attaches phenylalanine at the 2´ hydroxy group of its tRNA A76 ribose moiety [18, 19, 21, 23]. The structure of the PheRS enzyme together with its active site interactions have been extensively studied [24–30]. The PheRS aminoacylation active site contains three main binding pockets: the phenylalanine, ATP and tRNA binding pockets, which are located close to each other in all aaRS enzymes [22]. Studies have revealed that the most active aaRS inhibitors are those that have a dual inhibition activity for two or more of these binding pockets as they exhibit the highest binding affinity [22].

**Fig. 1:**
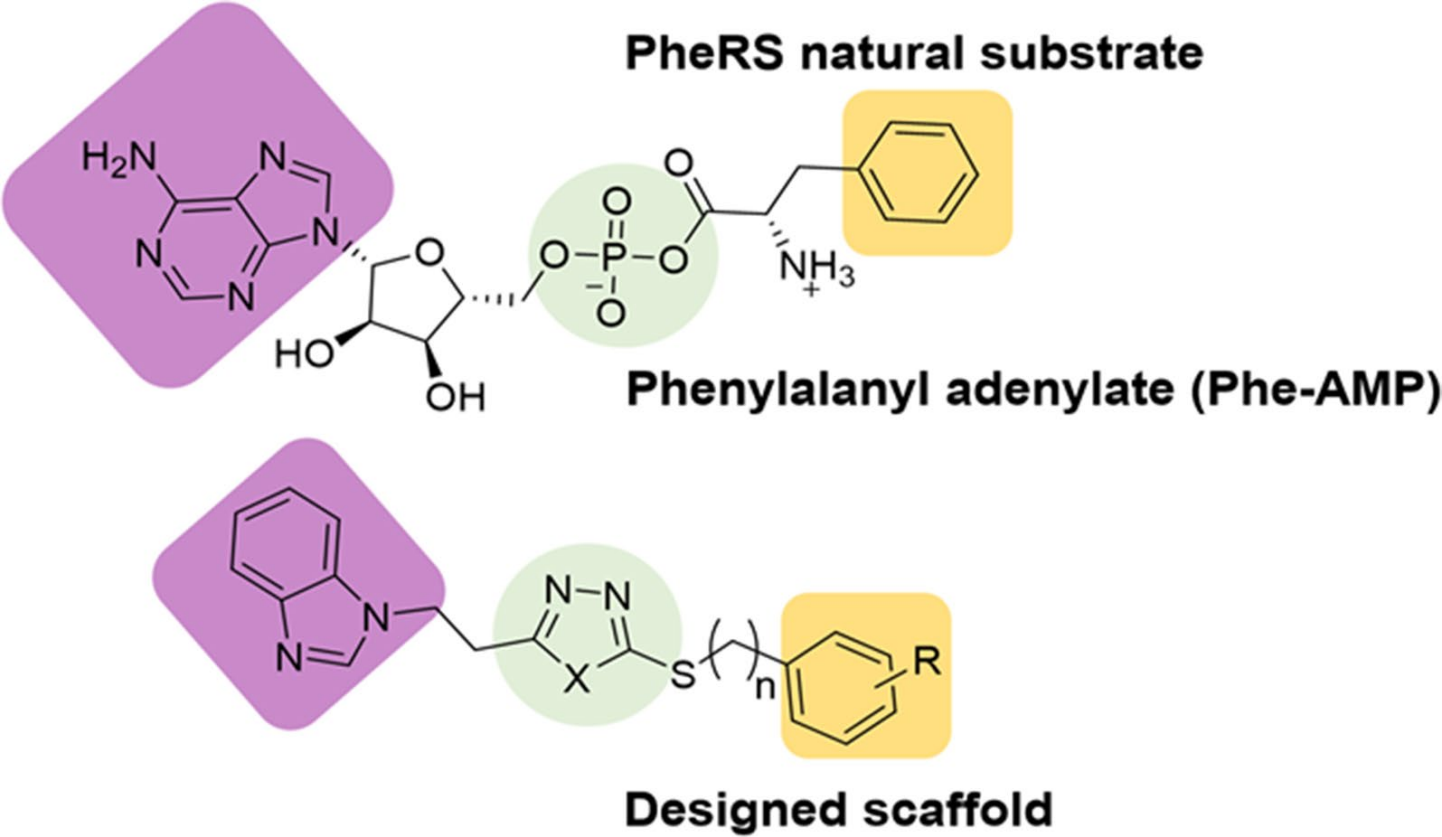
The main features of Phe-AMP and the corresponding structure of the designed compounds

**Fig. 2:**
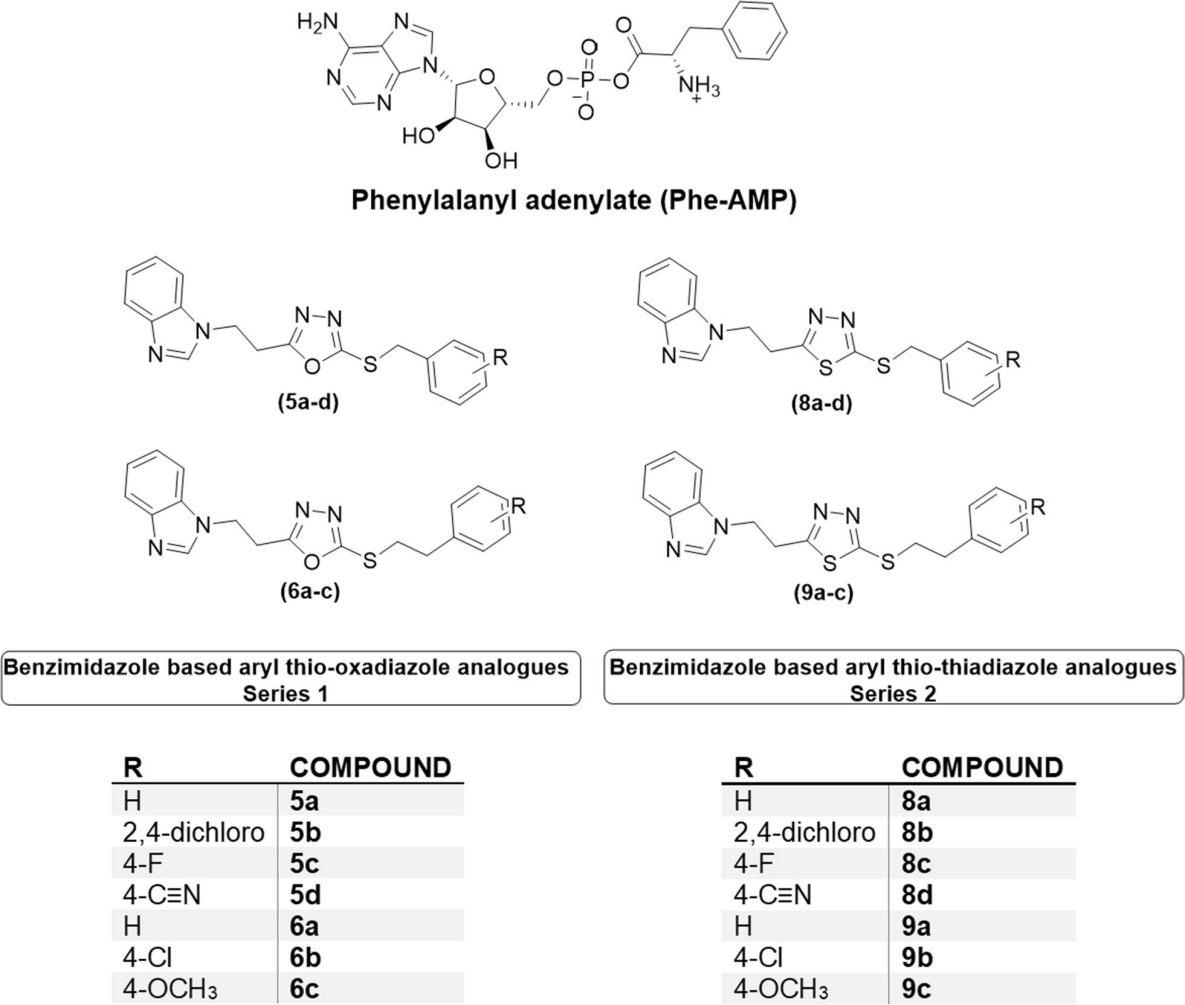
The structure of phenylalanyl adenylate (Phe-AMP) and the structures of series 1 and 2 designed compounds

This research describes the design and synthesis of PheRS inhibitors that have the ability to occupy both phenylalanine and ATP binding pockets at the same time. As the aminoacylation active site amino acid key residues are highly conserved among bacterial species, the design criteria should ensure the generation of broad-spectrum active antibiotics [9]. The compounds were designed to keep the same structural features of the Phe-AMP natural substrate to retain the same stabilising conserved interactions within the active aminoacylation pockets (Fig. 1). Based on this, two series of compounds; benzimidazole based aryl thio-oxadiazole analogues and benzimidazole based aryl thio-thiadiazole analogues were designed and examined by flexible alignment studies to ensure conformational similarity with the Phe-AMP natural substrate (Fig. 2). Furthermore, a molecular dynamics (MD) simulation comparative study was performed to understand the binding interactions of the designed compounds with PheRS from different bacterial microorganisms. The designed compounds were evaluated against a range of bacterial species to determine whether broad spectrum activity was obtained.

## Results and discussion

### Computational studies

**Fig. 3:**
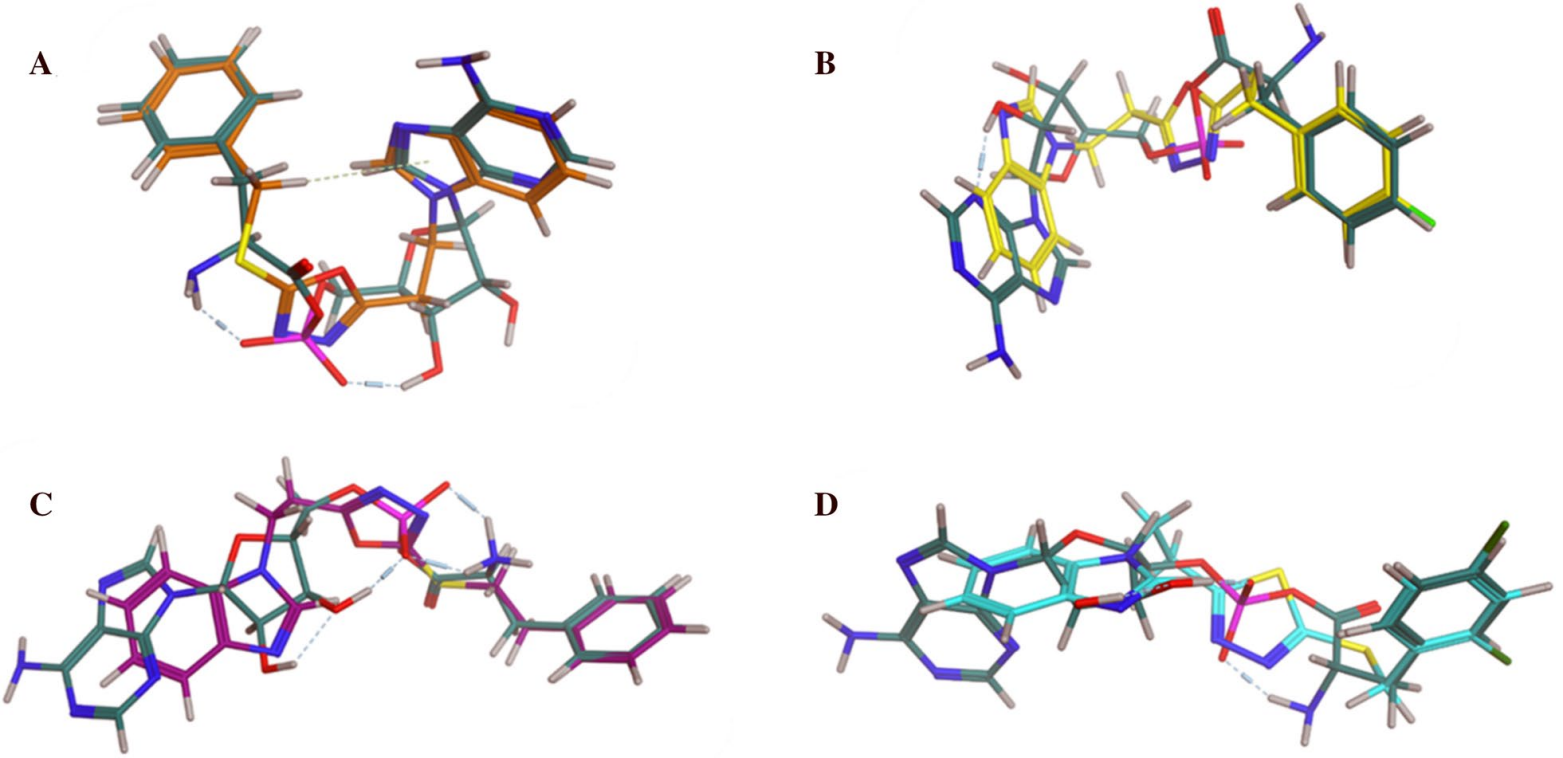
Flexible alignment results of Phe-AMP (green colour), **A** **5a** (orange colour) S = -111.9730, **B** **5c** (yellow colour) S = -115.9783, **C** **6a** (purple colour) S = -141.5878, **D** **8b** (light blue colour) S = -115.3427

The designed compounds were examined for their conformational similarity with the natural PheRS substrate Phe-AMP using the flexible alignment tool in Molecular Operating Environment (MOE) [31]. The results of some representative compounds from both series are shown in Fig. 3.

As revealed from the 3D flexible alignment results, the designed compounds have similar conformations to the natural substrate Phe-AMP. The benzimidazole moiety in the designed scaffold overlaps with the adenine moiety of Phe-AMP, and both the oxadiazole and thiadiazole moieties overlap with the phosphate group of the natural substrate, while the terminal aromatic moiety aligns with the phenylalanine aromatic ring. As the complementary moieties share nearly the same chemical features, and the designed compounds share the same 3D conformation of the natural substrate, it was expected that they would share the same active site interactions.

**Fig. 4:**
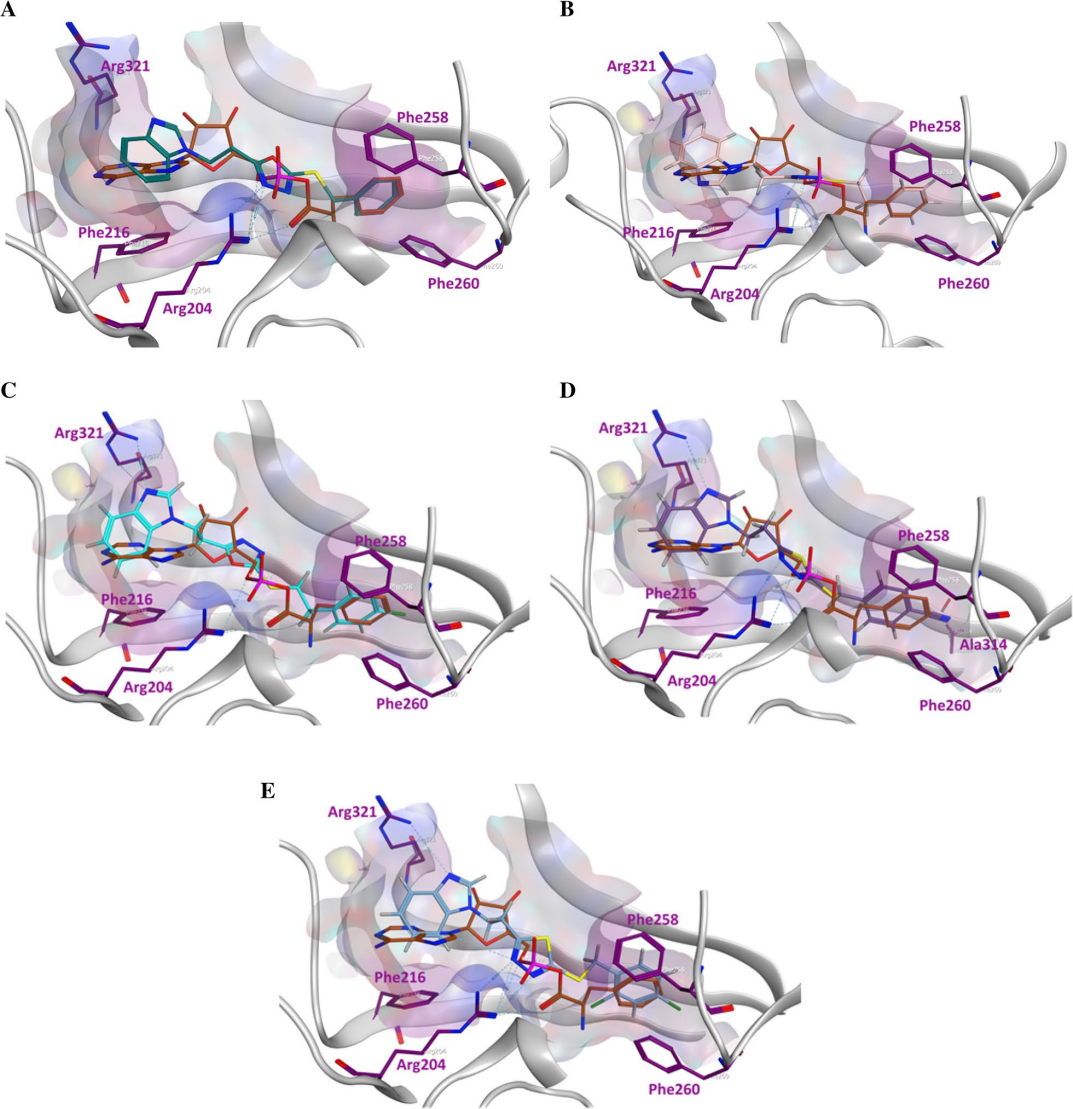
Docking results of some of the designed compounds in the aminoacylation active site of *Th. thermophilus* PheRS [pdb: 1JJC] [32]. The Phe-AMP (orange colour) was placed to compare the binding mode of the natural substrate and the designed compound. The illustrated designed compounds are **A** **5a** (green colour), **B** **6a** (pink colour), **C** **6b** (light blue colour), **D** **8d** (violet colour) and **E** **8b** (blue colour). Some key residues in the active site are shown: Phe216 stabilises the adenine of Phe-AMP in the ATP binding site, Arg204 and Arg312 are responsible for hydrophilic interactions of AMP in the ATP binding pocket, and Phe258 and Phe260 stabilise the phenyl ring of the phenylalanine in the binding pocket with π-π stacking

**Fig. 5:**
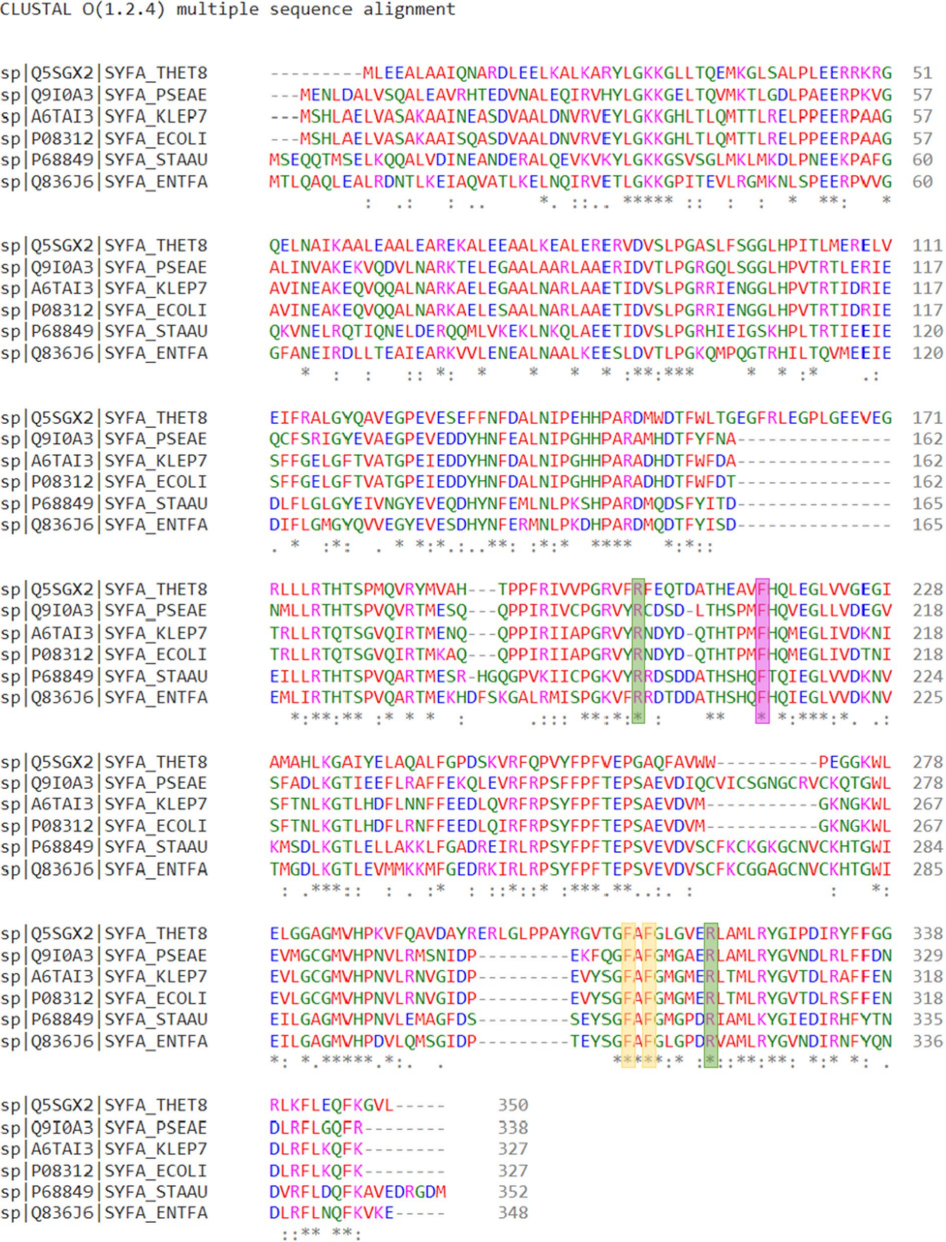
Multiple sequence alignment [33] of PheRS α subunit amino acid sequences from *Th. thermophilus (*Q5SGX2*), P. aeruginosa* (Q9I0A3), *K. pneumoniae* (A6TAI3), *E. coli* (P08312), *S. aureus* (P68849) and *E. faecalis* (Q836J6). The purple box shows the conserved phenylalanine residue responsible for hydrophobic stabilisation of the adenine moiety in Phe-AMP. The green boxes show the conserved arginine residues responsible for the hydrophilic interactions that stabilise the AMP moiety of the natural substrate. The yellow boxes show the conserved phenylalanine residues responsible for positioning of the phenyl ring of the Phe-AMP by face-to-edge hydrophobic interactions

A docking study using *Thermus thermophilus* PheRS [32] was performed (Fig. 4), *Th. thermophilus* was chosen for this study as it is the only resolved crystal structure with the complete enzyme substrate (Phe-AMP), while other available crystal structures were resolved with either unbound substrate or with inhibitors. Also, a multiple sequence alignment using PheRS amino acid sequences from five different bacterial species was performed to ensure the high level of conservation between the aminoacylation active site key amino acid residues among bacterial strains (Fig. 5). The two series of compounds were designed to occupy the Phe-AMP binding pockets in the PheRS aminoacylation active site, and to have the same binding interactions of the natural substrate. Benzimidazole was used as the base for synthesis to mimic the adenine moiety of Phe-AMP and to stabilise the compound in the active site pocket by hydrophobic interactions and hydrogen bonding with the conserved phenylalanine and arginine residues in this position, respectively (Phe216 and Arg321 in *Th. thermophilus* PheRS). Benzimidazole was chosen rather than the more hydrophilic adenine to improve the overall hydrophobicity of the compounds with the aim of improving permeability. Either oxadiazole or thiadiazole rings were positioned to allow the hydrophilic interactions at the ATP binding pocket comparable with the phosphate group in Phe-AMP (Arg204 in *Th. thermophilus* PheRS). While the phenyl ring (free or substituted) was positioned to resemble the phenyl ring of the phenylalanine in Phe-AMP. The presence of an aryl group at this position is important to stabilise the structure in the Phe-AMP binding pocket with π stacking with the conserved phenylalanine residues in that position of the active site (Phe258 and Phe260 in *Th. thermophilus* PheRS). Docking studies suggested that the designed compounds can occupy the aminoacylation active site encountering the key amino acid residues responsible for the natural substrate binding (Fig. 4). Due to the high level of conservation between the PheRS aminoacylation active sites among bacteria as revealed from the multiple sequence alignment, the aim was to design broad-spectrum antimicrobial agents sharing the same binding criteria as the substrate (Fig. 5).

**Fig. 6:**
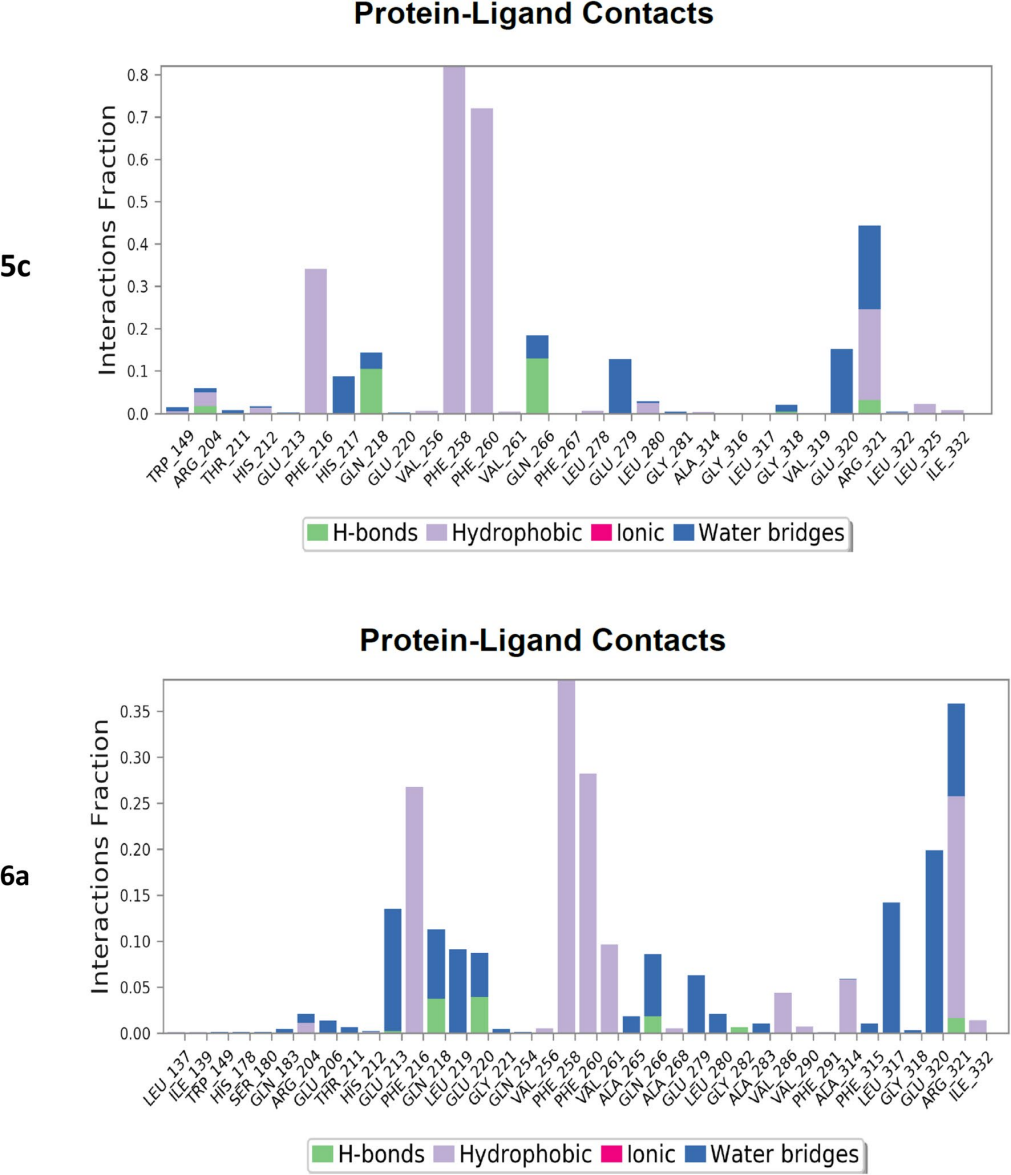

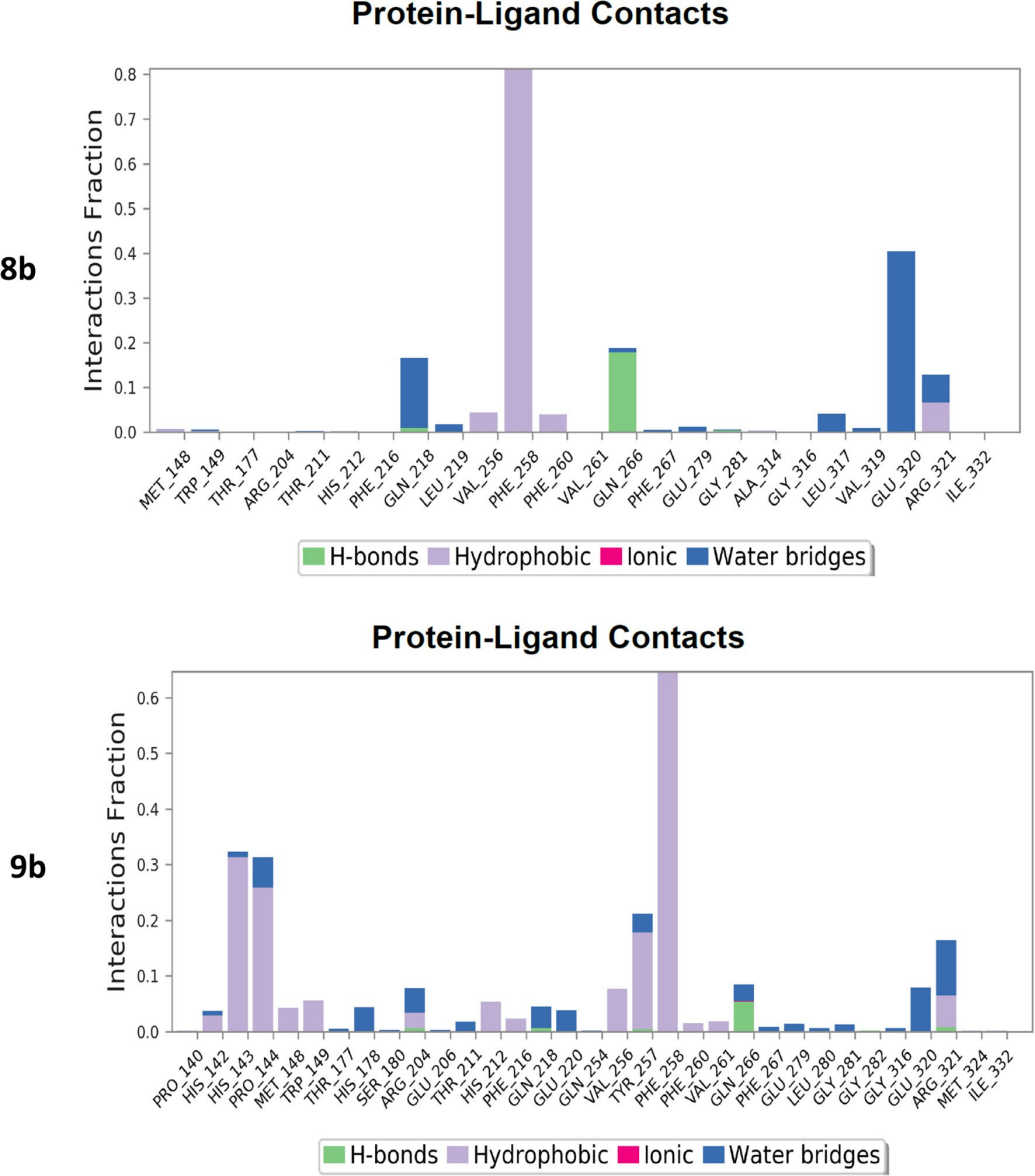
Simulation interaction diagrams showing the protein–ligand interactions with *Th. thermophilus* PheRS throughout the 200 ns simulation. The stacked bar charts are normalised over the course of the trajectory, for example a value of 0.4 indicates that the specific reaction is maintained 40% of the simulation time

Molecular dynamics was performed for compounds **5c**, **6a**, **8b** and **9b**. Each of the compounds chosen represents those with the best docking binding interaction in each series. The PheRS-ligand complexes were subjected to a 200 ns simulation (Fig. 6). The final frame of compound **5c** showed the stabilisation of the compound in the phenylalanine binding pocket by hydrophobic interaction between the *para*-fluorophenyl group and the key amino acid residue Phe260, and the stabilisation of the compound in the binding pocket by hydrophilic interaction between the sulphide group and Gly316, while the benzimidazole moiety of the structure moved away from the adenine binding pocket. It is important to mention that the protein- ligand **5c** complex did not reach equilibrium during the simulation time. The final frame of compound **6a** showed the best fit in all binding pockets with the structure stabilised in the phenylalanine binding pocket via π stacking with the key amino residues Phe258 and Phe260. Compound **6a** was also stabilised in the phosphate binding pocket by hydrogen bonding between the sulphide group and Gly316 and stabilised in the ribose binding pocket by hydrophobic interaction with Arg204 and water mediated interaction with Met148, while being stabilised in the adenine binding pocket by hydrophobic interaction with Phe216. However, the final frame interactions obtained for compounds **8b** and **9b** were unexpected. Both compounds protruded outside the binding pockets while remaining connected to the active site via hydrophobic interaction with Phe258 that were maintained for more than 60% of the simulation time for the two compounds. From these computational data it was expected that series 1 compounds would show better activity than series 2 compounds.

**Fig. 7:**
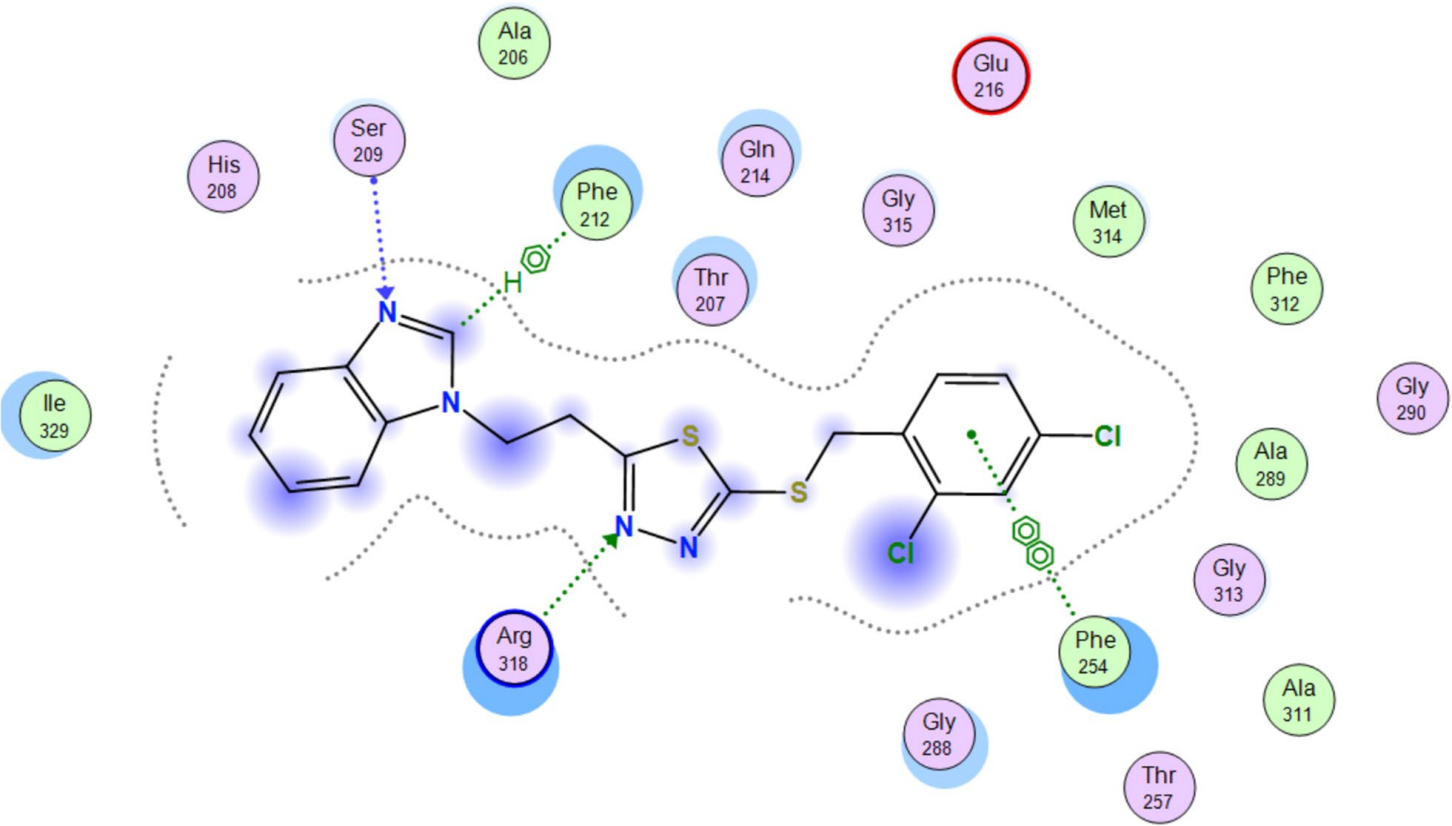
2D ligand interactions of the final frame of **8b** with *S. aureus* PheRS homology model after MD simulation

**Fig. 8:**
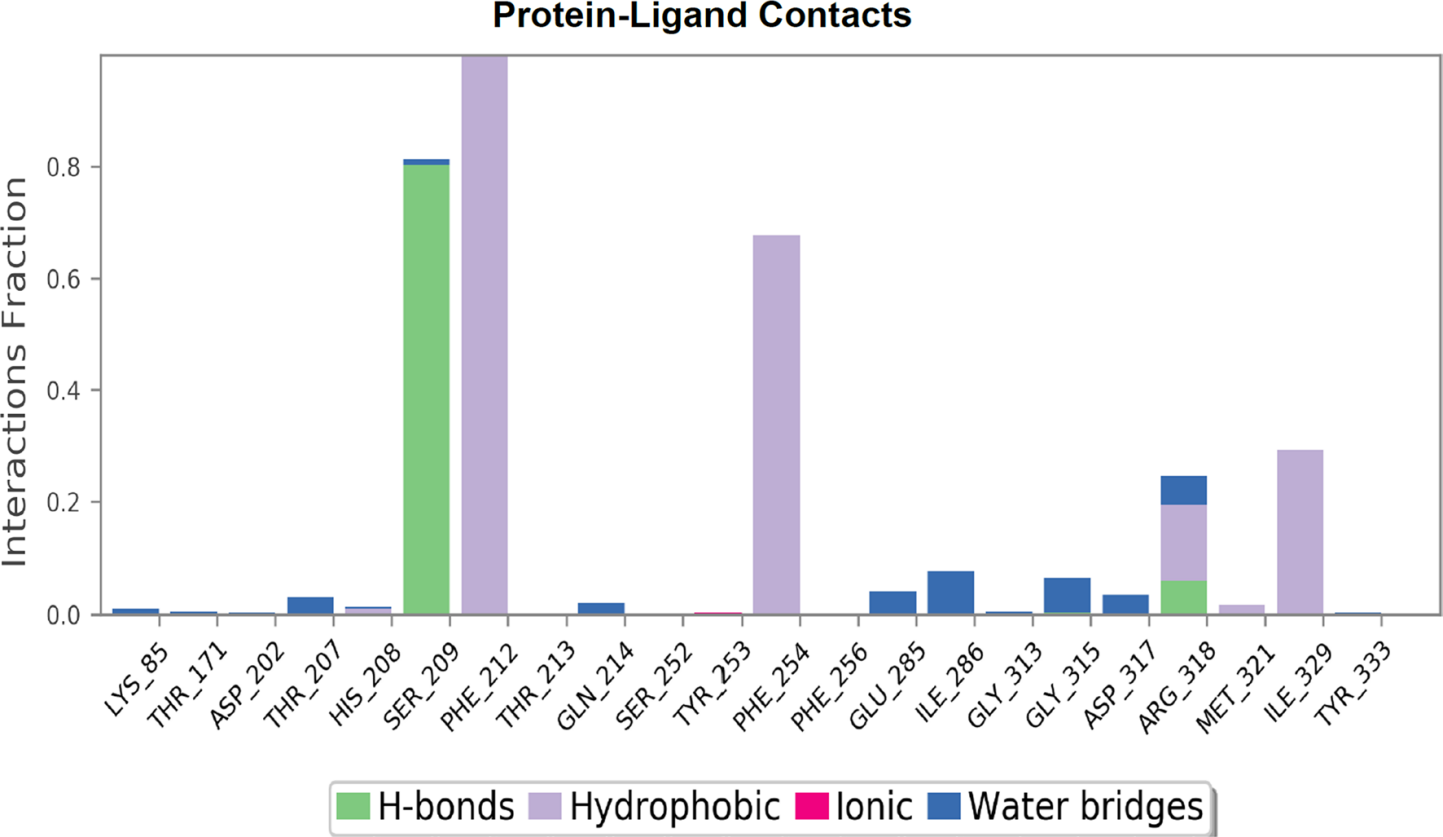
Diagram showing the *S. aureus* PheRS homology model—compound **8b** interactions throughout the 200 ns simulation. The stacked bar charts are normalised over the course of the trajectory, for example; a value of 0.4 indicates the specific reaction is maintained 40% of the simulation time

Unexpectedly, the microbiological evaluation using *S. aureus*, *E. faecalis*, *P. aeruginosa*, *K. pneumoniae* and *E. coli* revealed that compound **8b** from series 2 had the best activity against *S. aureus* (MIC 32 μg/mL), however, it had poor molecular interactions with *Th. thermophilus* PheRS. All the compounds from the two series showed weak inhibitory activity against *E. faecalis* and *P. aeruginosa* (MIC 128 μg/mL) and were inactive against both *E. coli* and *K. pneumoniae*. To provide a clearer insight of these unexpected results on a molecular level a published homology model of *S. aureus* PheRS [34] was used to study the interactions between **8b** and the *S. aureus* PheRS enzyme using MD simulation. On visualising the final frame 2D interactions of the simulation using MOE software (Fig. 7) hydrophobic interactions with both the conserved Phe212 and the conserved Phe254 stabilising the structure in the right pocket were observed. Moreover, hydrogen bonding between N3 in the thiadiazole ring and the conserved Arg318 (Arg321 in *Th. thermophilus* PheRS) and between N3 in the benzimidazole ring and Ser209 were observed. On monitoring the MD protein ligand diagram (Fig. 8) it shows strong hydrophobic stabilisation of the compound inside the right pocket throughout the simulation time with both Phe212 (interaction maintained more than 80% of the simulation time) and Phe254 (interaction maintained for more than 60% of the simulation time). A strong hydrogen bond was maintained for about 80% of the simulation time with Ser209 and fair hydrophilic interactions with Arg318 were maintained for more than 20% of the simulation time. The strong hydrogen bonding with Ser209 seems to be highly important to hold the structure in the ATP binding site, stabilising the structure in the intended conformation contributing to the more promising activity. This agrees with the results revealed in a study by Wang et al*.* [29], which found that good interactions in the ATP binding site offers a great opportunity to inhibit both phenylalanine and ATP binding pockets resulting in a bi-substrate promising inhibitor.

**Fig. 9:**
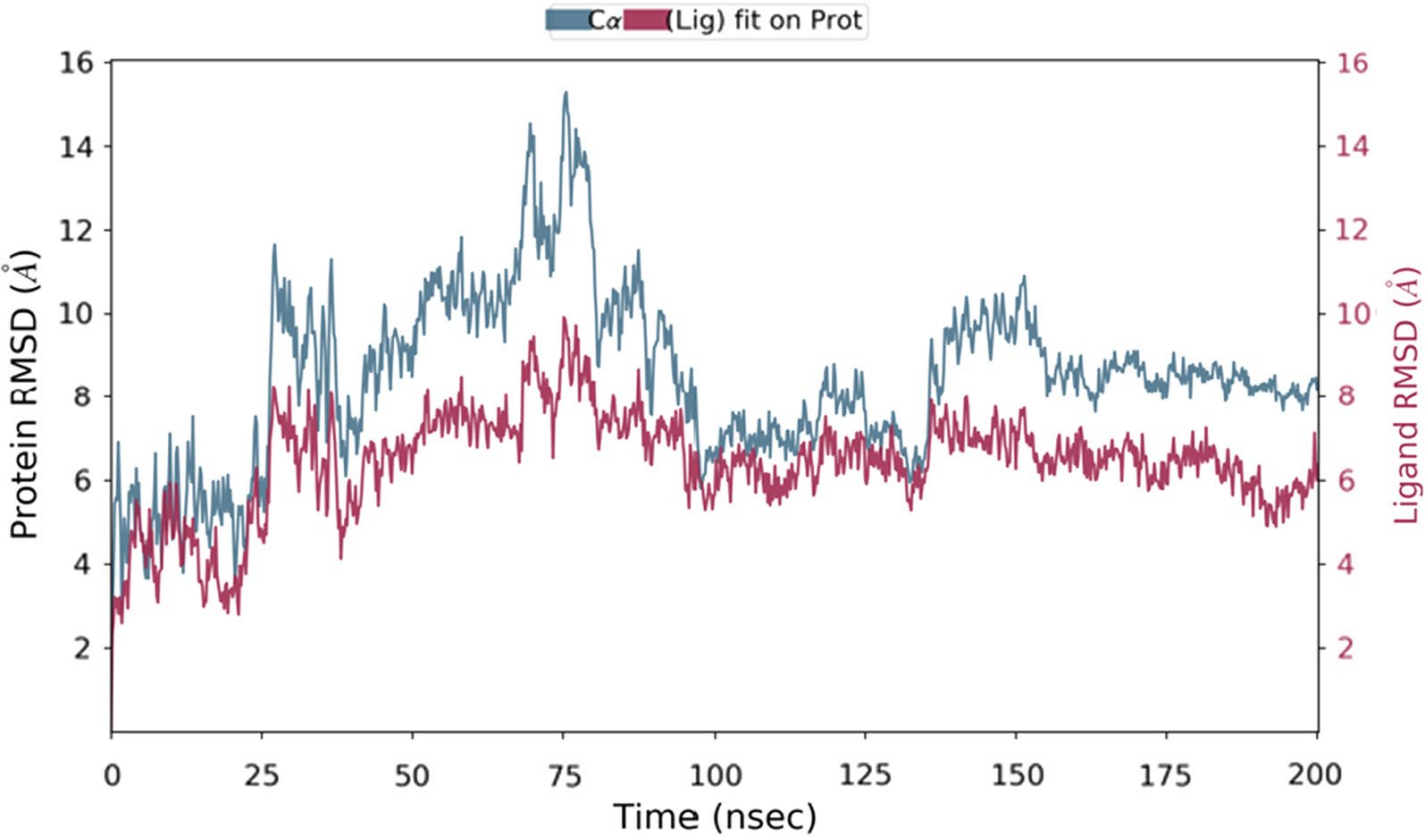
RMSD [Å] plot with respect to time in nanoseconds during 200 ns MD simulation of *S. aureus* homology model and compound **8b** complex

The binding free energy (ΔG) for compound **8b** complexed with the *S. aureus* homology model was calculated from each frame starting from the last 100 ns with respect to the RMSD (Fig. 9). The calculated ΔG of -69.0431 ± 3.23 kcal/mol indicated an optimal positioning within the active site and a high ligand binding affinity.

**Fig. 10:**
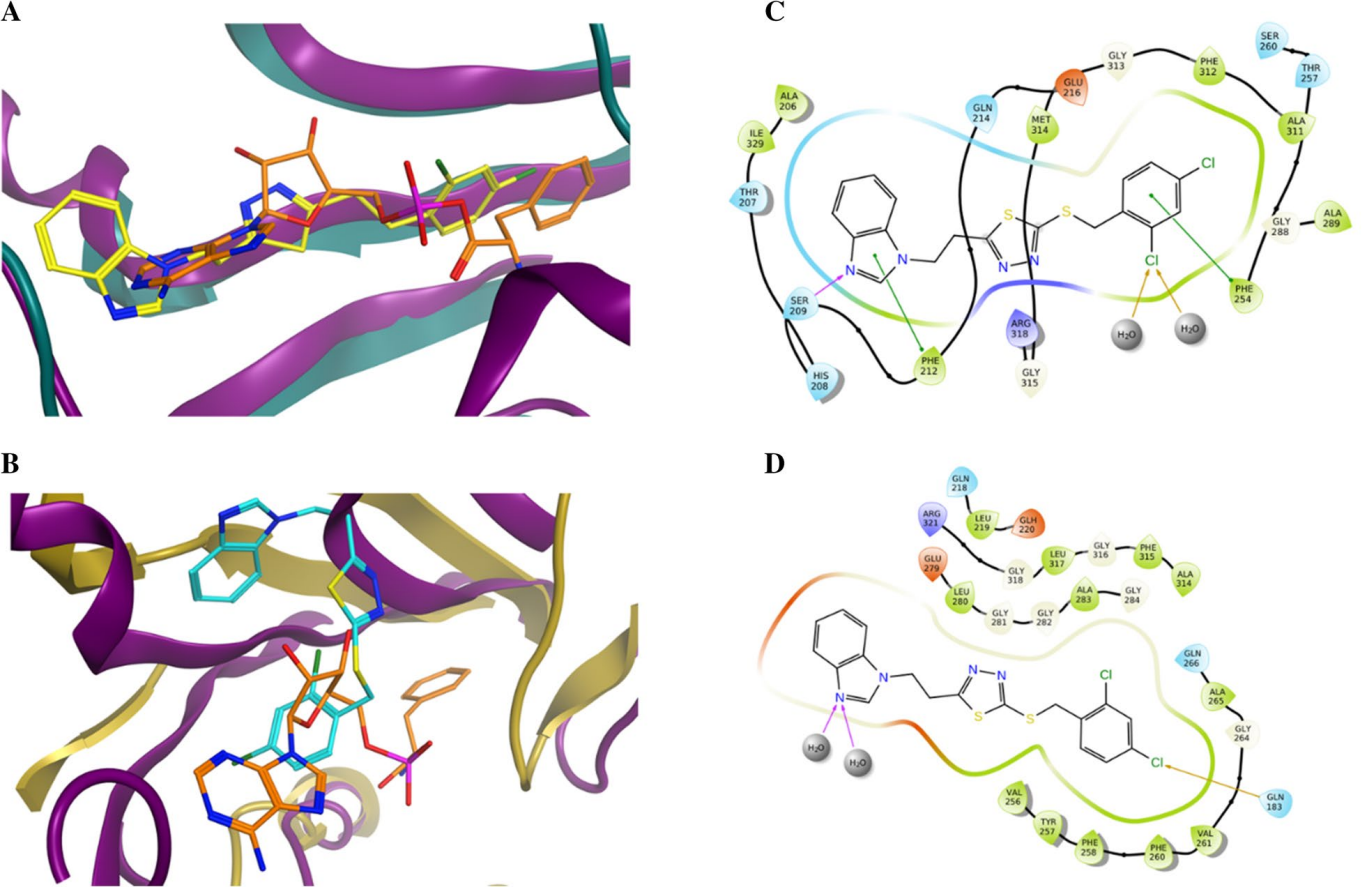
Illustrating the difference between **8b** interactions with *Th. thermophilus* PheRS and *S. aureus* PheRS. A) The purple ribbon is *Th. thermophilus* PheRS co-crystallised with Phe-AMP (orange colour) superimposed with the *S. aureus* PheRS homology model (green ribbon) and the MD simulation final frame of **8b** (yellow colour), B) The purple ribbon is *Th. thermophilus* PheRS co-crystallised with Phe-AMP (orange colour) superimposed with the *Th. thermophilus* PheRS (golden ribbon) and the MD simulation final frame of **8b** (light blue colour), C) the 2D ligand interactions of the MD simulation final frame of **8b** with *S. aureus* PheRS homology model, D) the 2D ligand interactions of the MD simulation final frame of **8b** with *Th. thermophilus* PheRS

These results highlighted that despite the active site residues conservation in various bacterial PheRSs, there are distinct differences between inhibitor interactions, as illustrated by the MD interactions of **8b**, which were completely different against both *S. aureus* and *Th. thermophilus* PheRSs (Fig. 10). This difference in ligand–protein interactions would make it more difficult to obtain a broad-spectrum antimicrobial agent from such a design strategy. Despite this, compound **8b** (benzimidazole based thio-thiadiazole) was identified as a bi-substrate lead compound for further structural development to obtain high affinity *S. aureus* PheRS inhibitors. Future design strategies of PheRS inhibitors should focus on maintaining strong binding interactions in the ATP binding pocket to ensure correct positioning of the designed compounds in the Phe-AMP binding sites to achieve both high inhibitory activity and low resistance opportunity.

### Chemistry

**Fig. 11:**
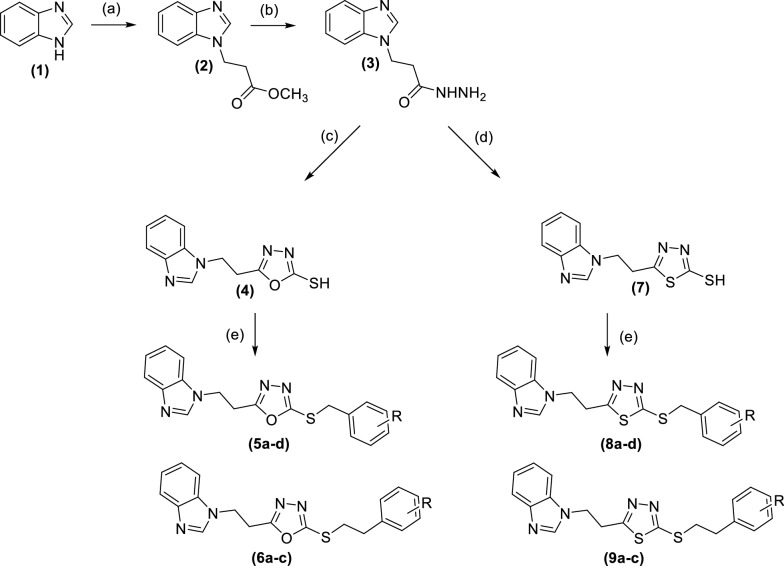
Scheme of preparation of compounds **5a-d**, **6a-c**, **8a-d** and **9a-c** (a) dry K_2_CO_3_, 18-crown-6, methyl 3-bromopropionate, anhydrous DMF, overnight, 60 °C (b) NH_2_NH_2_. H_2_O, ethanol, overnight, r.t (c) KOH, CS_2_, absolute ethanol, overnight, 80 °C, 1 N HCl/H_2_O, (d) KOH, CS_2_, absolute ethanol, overnight, 80 °C, conc.H_2_SO_4_/H_2_O (e) K_2_CO_3_, anhydrous DMF, appropriate alkyl halide, overnight, r.t

Compounds **5a**-**d**, **6a**-**c**, **8a**-**d** and **9a**-**c** were then synthesised following Fig. 11.

Methyl 3-(*1H*-benzo[*d*]imidazol-1-yl)propanoate **(2)** was synthesised by a nucleophilic substitution reaction between benzimidazole **(1)** and methyl 3-bromopropionate in the presence of K_2_CO_3_ and 18-crown-6, which is used to enhance the ionisation of K_2_CO_3_ in DMF [35–37]. 3-(*1H*-Benzo[*d*]imidazol-1-yl)propane hydrazide **(3)** was synthesised by hydrazinolysis of methyl 3-(*1H*-benzo[*d*]imidazol-1-yl)propanoate **(2)** using excess hydrazine hydrate at room temperature in EtOH [38, 39]. The hydrazide intermediate was used to synthesise two more intermediates, **(4)** and **(7)**, following the reported procedure [40]. The hydrazide was first refluxed with KOH and carbon disulphide to afford 2-(3-(1*H*-benzo[*d*]imidazol-1-yl)propanoyl)hydrazine-1-carbodithioate intermediate, which was cyclised either into 5-[2-(1*H*-benzo[*d*]imidazol-1-yl)ethyl)-1,3,4-oxadiazole-2-thiol **(4)** by acidification with 1 N aqueous HCl or into 5-(2-(1*H*-benzo[*d*]imidazol-1-yl)ethyl]-1,3,4-thiadiazole-2-thiol **(7)** by acidification with conc. H_2_SO_4_, with structure confirmation by high resolution mass spectroscopy. Reaction of 5-[2-(1*H*-benzo[*d*]imidazol-1-yl)ethyl]-1,3,4-oxadiazole-2-thiol **(4)** and 5-[2-(1*H*-benzo[*d*]imidazol-1-yl)ethyl]-1,3,4-thiadiazole-2-thiol **(7)** with various alkyl halides in the presence of K_2_CO_3_ afforded the target compounds **5a**-**d**, **6a**-**c**, **8a**-**d** and **9a**-**c** (Table 1).

**Table 1:** Yields and melting points (m.p.) of compounds **5a-d**, **6a-c**, **8a-d** and **9a-c**

Series 1			Series 2		
Compounds	% yield	m.p. (°C)	Compounds	% yield	m.p. (°C)
5a	59	80–82	**8a**	62	88–90
5b	79	86–88	**8b**	69	84–86
5c	71	semisolid	**8c**	71	76–78
5d	67	semisolid	**8d**	61	112–114
6a	60	78–80	**9a**	51	76–78
6b	65	90–92	**9b**	40	96–98
6c	75	65–67	**9c**	55	68–70

### Microbiological evaluation

All series 1 and 2 compounds were tested against *S. aureus*, *E. faecalis*, *P. aeruginosa*, *E. coli* and *K. pneumoniae* using ciprofloxacin as a reference drug. Microbiological evaluation indicated a moderate activity of compound **8b** against *S. aureus* (MIC 32 μg/mL). The structurally similar compound **5b** showed weak inhibitory activity against *S. aureus* (MIC 128 μg/mL), while all other compounds had poor activity against *S. aureus* (MIC > 128 μg/mL). All the compounds showed weak inhibitory activity against both *E. faecalis* and *P. aeruginosa* (MIC 128 μg/mL), and all the compounds were inactive against both *E. coli* and *K. pneumoniae* (MIC > 128 μg/mL) except for compound **6c,** which had weak inhibitory activity against *K. pneumoniae* (MIC 128 μg/mL).

## Conclusion

Development of PheRS inhibitors has been a promising strategy with good antimicrobial results [29, 30, 41–45]. In particular, drugs that have a dual inhibition of both phenylalanine and ATP binding pockets have the highest binding affinity [22]. Two series of compounds were designed to occupy both phenylalanine and ATP binding pockets, and to mimic Phe-AMP to retain the main stabilising binding interactions of the natural substrate. A comparative computational study revealed that, although the PheRS amino acid sequence is highly conserved among bacterial species, their interactions with enzyme inhibitors are completely different. The reasons behind such findings need more structural and crystallographic studies to optimise the rational design of inhibitors. Compounds were synthesised and evaluated for their in vitro antimicrobial activity against five bacterial strains (Gram + *ve* and Gram *-ve*). Only compound **8b** showed a moderate activity against *S. aureus* with MIC of 32 μg/mL.

## Experimental

### Chemistry

#### General

All reagents and solvents employed were of general purpose or analytical grade and purchased from Fluka, Acros, Alfa-Aeser and Sigma-Aldrich. Solvents were appropriately dried over molecular sieves (4 Å). ^1^H and ^13^C NMR spectra were recorded on a Bruker Advance DP500 spectrometer operating at 500 MHz and 125 MHz respectively. Chemical shifts are given in parts per million (ppm) relative to the internal standard tetramethylsilane (Me_4_Si). Coupling constants (J value) were calculated in hertz (Hz). An additional file shows these spectra in more details (see Additional file 1). Silica gel Fluka Kieselgel 60, particle size 35–70 μm Davisil® chromatography grade, was used for column chromatography in a glass column. Gradient column chromatography was performed with the aid of a pump. Analytical thin layer chromatography (TLC) was carried out on precoated silica plates (ALUGRAM® SIL G/UV254) with visualisation via UV light (254 nm). Melting points were determined using a Gallenkamp melting point apparatus and are uncorrected. UV HPLC was performed at the University of Bath using a sheath gas temperature of 350 °C, flow rate of 12 L/min, and nebuliser gas at 45 psi (3.10 bar). MS was calibrated using reference calibrant introduced from the independent ESI reference sprayer. Chromatographic separation was performed on a Zorbax Eclipse Plus C18 Rapid Resolution column 2.1 × 50 mm, 1.8 µm particle size using H_2_O (Merck, LC–MS grade) with 0.1% formic acid (FA, Fluka) v/v and methanol (MeOH, VWR, HiPerSolv) with 0.1% FA v/v as mobile phase A and B, respectively.

Compounds **2** and **3** were prepared as previously described [35–39, 46].

#### 5- [2-(1*H*-benzo[*d*]imidazol-1-yl)ethyl]-1,3,4-oxadiazole-2-thiol (4)

A mixture of 3-(1*H*-benzo[*d*]imidazol-1-yl)propane hydrazide (**3**) (1 g, 4.9 mmol) and KOH (0.27 g, 4.9 mmol) in absolute EtOH (12 mL) was allowed to stir at room temperature for 30 min, then CS_2_ (0.56 g, 7.35 mmol) was added and the reaction mixture was heated under reflux overnight. The solvent was removed under vacuum and H_2_O (20 mL) was added and the resulting solution was cooled and acidified with 1 N aqueous HCl. The formed solid was collected by filtration, washed with H_2_O and dried under vacuum at 40 °C to obtain the product as a light brown solid: Yield: 1.11 g (92%); m.p. 225–227 °C; HPLC: 95.65% at R.T. 3.27 min; ^1^H NMR (DMSO-*d*_*6*_) δ: 3.33 (t, *J* = 6.8 Hz, 2H, NCH_2_CH_2_), 4.65 (t, *J* = 6.8 Hz, 2H, NCH_2_), 7.22 (td, *J* = 1.2, 7.2 Hz, 1H, Ar), 7.28 (td, *J* = 1.3, 8.2 Hz, 1H, Ar), 7.64 (dd, *J* = 7.9, 13.2 Hz, 2H, Ar), 8.25 (s, 1H, Imidazole).^13^C NMR (DMSO-*d*_*6*_) δ: 26.5 (NCH_2_CH_2_), 40.7 (NCH_2_), 110.8 (CH), 119.9 (CH), 122.2 (CH), 123.0 (CH), 133.9 (C), 143.5 (C), 144.5 (CH-imidazole), 161.9 (C), 178.2 (C). HRMS (ESI) *m/z* Calculated: 246.0575 [M + H]^+^, Found: 246.0583 [M + H]^+^.

#### 5- [2-(1*H*-benzo[*d*]imidazol-1-yl)ethyl]-1,3,4-thiadiazole-2-thiol (7)

A mixture of 3-(1*H*-benzo[*d*]imidazol-1-yl)propane hydrazide (**3**) (1 g, 4.9 mmol) and KOH (0.27 g, 4.9 mmol) in absolute EtOH (12 mL) was allowed to stir at room temperature for 30 min, then CS_2_ (0.56 g, 7.35 mmol) was added. The reaction mixture was heated under reflux overnight. The solvent was removed under vacuum and concentrated H_2_SO_4_ (25 mL) was added and the mixture was allowed to stir for 4 h. The reaction mixture was poured into crushed ice and stirred for 1 h, then the resulting solid was collected by filtration, washed several times with H_2_O and dried under vacuum at 40 °C to obtain the product as a beige solid: Yield: 0.78 g (60%); mp 232–234 °C; HPLC: 100% at R.T. 3.28 min. ^1^H NMR (DMSO-*d*_*6*_) δ: CH_2_ peak is obscured by H_2_O of DMSO-*d*_*6*_ peak, 4.80 (t, *J* = 6.7 Hz, 2H, NCH_2_), 7.46 (m, 2H, Ar), 7.78 (d, *J* = 7.3 Hz, 1H, Ar), 7.88 (d, *J* = 7.5 Hz, 1H, Ar), 9.01 (s, 1H, Imidazole). ^13^C NMR (DMSO-*d*_*6*_) δ: 25.9 (NCH_2_CH_2_), 41.9 (NCH_2_), 112.4 (CH), 117.4 (CH), 124.9 (CH), 125.1 (CH), 132.4 (C), 136.6 (C), 143.5 (CH-imidazole), 155.1 (C). HRMS (ESI) *m/z* Calculated: 262.0347 [M + H]^+^, Found: 262.0387 [M + H]^+^

#### General method for the preparation of 2-(2-(1*H*-benzo[*d*]imidazol-1-yl)ethyl)-5-(arylthio)-1,3,4-oxadiazole (5a-d, 6a-c) and 2-(2-(1*H*-benzo[*d*]imidazol-1-yl)ethyl)-5-(arylthio)-1,3,4-thiadiazole (8a-d, 9a-c)

A mixture of aromatic thiol (**4** or **7**) (1 equivalent) and K_2_CO_3_ (1.5 equivalent) in anhydrous DMF (8 mL/0.89 mmol) was allowed to stir at room temperature for 30 min. Then the appropriate alkyl halide (1.1 equivalent) in anhydrous DMF (4 mL/0.89 mmol) was added and the reaction mixture allowed to stir at room temperature overnight. The solvent was removed under vacuum and then H_2_O (50 mL/0.89 mmol) was added, and the mixture extracted with chloroform (3 × 50 mL/0.89 mmol). The combined organic layers were dried (MgSO_4_) and evaporated, then the product was purified using gradient column chromatography.

*2-{[2-(1H-benzo[d]imidazol-1-yl)ethyl]-5-(benzylthio)}-1,3,4-oxadiazole* **(5a).**

Prepared from 5-(2-(1*H*-benzo[*d*]imidazol-1-yl)ethyl)-1,3,4-oxadiazole-2-thiol (**4**) (0.2 g, 0.813 mmol) and benzyl chloride (0.11 g, 0.89 mmol). Purified using gradient chromatography eluting with 100% EtOAc to afford the product as a white solid: Yield: 0.16 g (59%); mp 80–82 °C; TLC: 100% EtOAc, R_f_ 0.22; HPLC: 100% at R.T. 4.25 min. ^1^H NMR (DMSO-*d*_*6*_) δ: 3.44 (t, *J* = 6.8 Hz, 2H, NCH_2_CH_2_), 4.40 (s, 2H, SCH_2_), 4.67 (t, *J* = 6.7 Hz, 2H, NCH_2_), 7.19–7.34 (m, 5H, Ar), 7.37 (m, 2H, Ar), 7.57 (dd, *J* = 1.2, 7.3 Hz, 1H, Ar), 7.64 (dd, *J* = 0.9, 7 Hz,1H, Ar), 8.19 (s, 1H, CH-imidazole). ^13^C NMR (DMSO-*d*_*6*_) δ: 26.2 (NCH_2_CH_2_), 36.1 (SCH_2_), 41.2 (NCH_2_), 110.7 (CH), 120.0 (CH), 122.1 (CH), 122.9 (CH), 128.2 (CH), 129.0 (2xCH), 129.4 (2xCH), 133.9 (C), 136.9 (C), 143.8 (C), 144.5 (CH-imidazole), 163.6 (C), 165.9 (C). HRMS (ESI) *m/z* Calculated: 336.1045 [M + H]^+^, Found: 336.1056 [M + H]^+^.

*2-{[2-(1H-benzo[d]imidazol-1-yl)ethyl]-5- [(2,4-dichlorobenzyl)thio]}-1,3,4-oxadiazole* **(5b).**

Prepared from 5-(2-(1*H*-benzo[*d*]imidazol-1-yl)ethyl)-1,3,4-oxadiazole-2-thiol (**4**) (0.2 g, 0.813 mmol) and 2,4-dichlorobenzyl chloride (0.17 g, 0.89 mmol). Purified using gradient chromatography eluting with 100% EtOAc to afford the product as a white solid: Yield: 0.26 g (79%); mp 86–88 °C; TLC: 100% EtOAc, R_f_ 0.36; HPLC: 100% at R.T. 4.62 min. ^1^H NMR (DMSO-*d*_*6*_) δ: 3.44 (t, *J* = 6.8 Hz, 2H, NCH_2_CH_2_), 4.47 (s, 2H, SCH_2_), 4.67 (t, *J* = 6.8 Hz, 2H, NCH_2_), 7.21 (m, 2H, Ar), 7.38 (dd, *J* = 2.2, 8.3 Hz, 1H, Ar), 7.49 (d, *J* = 8.4 Hz, 1H, Ar), 7.56 (dd, *J* = 1.7, 7.2 Hz, 1H, Ar), 7.64 (m, 2H, Ar), 8.21 (s, 1H, imidazole). ^13^C NMR (DMSO-*d*_*6*_) δ: 26.3 (NCH_2_CH_2_), 33.9 (SCH_2_), 41.2 (NCH_2_), 110.6 (CH), 120.0 (CH), 122.1 (CH), 122.9 (CH), 128.0 (CH), 129.6 (CH), 133.2 (CH), 133.5 (C), 133.9 (C), 134.0 (C), 134.7 (C), 143.8 (C), 144.6 (CH-imidazole), 162.9 (C), 166.2 (C). HRMS (ESI) *m/z* Calculated: 404.0265 [M + H]^+^, Found: 404.0276 [M + H]^+^.

*2-{[2-(1H-benzo[d]imidazol-1-yl)ethyl]-5- [(4-fluorobenzyl)thio]}-1,3,4-oxadiazole* **(5c).**

Prepared from 5-(2-(1*H*-benzo[*d*]imidazol-1-yl)ethyl)-1,3,4-oxadiazole-2-thiol (**4**) (0.2 g, 0.813 mmol) and 4-flourobenzyl chloride (0.13 g, 0.89 mmol). Purified using gradient chromatography eluting with 100% EtOAc to afford the product as a white semisolid: Yield: 0.205 g (71%); TLC: 100% EtOAc, R_f_ 0.20; HPLC: 100% at R.T. 4.28 min. ^1^H NMR (DMSO-*d*_*6*_) δ: 3.44 (t, *J* = 6.7 Hz, 2H, NCH_2_CH_2_), 4.40 (s, 2H, SCH_2_), 4.67 (t, *J* = 6.7 Hz, 2H, NCH_2_), 7.14 (t, *J* = 8.9 Hz, 2H, Ar), 7.22 (m, 2H, Ar), 7.42 (m, 2H, Ar), 7.57 (dd, *J* = 1.3, 7.3 Hz, 1H, Ar), 7.64 (dd, *J* = 1, 6.9 Hz, 1H, Ar), 8.19 (s, 1H, imidazole). ^13^C NMR (DMSO-*d*_*6*_) δ: 26.2 (NCH_2_CH_2_), 35.3 (SCH_2_), 41. (NCH_2_), 110.7 (CH), 115.7 (CH), 115.9 (CH), 120.0 (CH), 122.1 (CH), 122.9 (CH), 131.5 (CH), 131.6 (CH), 131.3 (C), 133.9 (C), 143.8, (C), 144.6 (CH-imidazole), 161.1 (C), 163.0 (C), 163.5 (C), 165.9 (C). HRMS (ESI): Calculated: 354.0951 [M + H]^+^, Found: 354.0961 [M + H]^+^.

*4-{[( 5- [2-(1H-benzo[d]imidazol-1-yl)ethyl]-1,3,4-oxadiazol-2-yl)}thio)methyl] benzonitrile* **(5d).**

Prepared from 5-(2-(1*H*-benzo[*d*]imidazol-1-yl)ethyl)-1,3,4-oxadiazole-2-thiol (**4**) (0.2 g, 0.813 mmol) and 4-(chloromethyl)benzonitrile (0.14 g, 0.89 mmol). Purified using gradient chromatography eluting with 100% EtOAc to afford the product as a white semisolid: Yield: 0.195 g (67%); TLC: 100% EtOAc, R_f_ 0.36; HPLC: 100% at R.T. 4.02 min. ^1^H NMR (DMSO-*d*_*6*_) δ: 3.43 (t, *J* = 6.8 Hz, 2H, NCH_2_CH_2_), 4.48 (s, 2H, SCH_2_), 4.66 (t, *J* = 6.7 Hz, 2H, NCH_2_), 7.21 (m, 2H, Ar), 7.56 (m, 3H, Ar), 7.64 (m, 1H, Ar), 7.77 (d, *J* = 8.4 Hz, 2H, Ar), 8.19 (s, 1H, imidazole). ^13^C NMR (DMSO-*d*_*6*_) δ: 26.2 (NCH_2_CH_2_), 35.5 (SCH_2_), 41.2 (NCH_2_), 110.6 (CH), 110.9 (CN), 119.1 (C), 119.9 (CH), 122.1 (CH), 122.9 (CH), 130.4 (2xCH), 132.9 (2xCH), 133.9 (C), 143.2, (C), 143.8 (C), 144.6 (CH-imidazole), 163.2 (C), 166.1 (C). HRMS (ESI): Calculated: 361.0997 [M + H]^+^, Found: 361.1004 [M + H]^+^.

*2- {[2-(1H-benzo[d]imidazol-1-yl)ethyl]-5-(phenethylthio)}-1,3,4-oxadiazole* **(6a).**

Prepared from 5-(2-(1*H*-benzo[*d*]imidazol-1-yl)ethyl)-1,3,4-oxadiazole-2-thiol (**4**) (0.2 g, 0.813 mmol) and 2-bromomethyl benzene (0.166 g, 0.89 mmol). Purified using gradient chromatography eluting with CH_2_Cl_2_-MeOH 97.5:2.5 v/v to afford the product as a white semisolid: Yield: 0.166 g (62%); mp 78–80 °C; TLC: CH_2_Cl_2_-MeOH 9:1 v/v, R_f_ 0.55; HPLC: 100% at RT = 4.35 min. ^1^H NMR (DMSO-*d*_*6*_) δ: 2.96 (t, *J* = 7.2 Hz 2H, SCH_2_CH_2_), CH_2_ signal is obscured by H_2_O in DMSO-*d*_*6*_ signal, 3.44 (t, *J* = 6.7 Hz, 2H, SCH_2_), 4.69 (t, *J* = 6.7 HzH, NCH_2_), 7.19–7.31 (m, 7H, Ar), 7.59 (d, *J* = 7.9 Hz, 1H, Ar), 7.63 (d, *J* = 7.8 Hz, 1.0 Hz, 1H, Ar), 8.22 (s, 1H, imidazole). ^13^C NMR (DMSO-*d*_*6*_) δ: 26.2 (NCH_2_CH_2_), 33.6 (SCH_2_), 35.2 (SCH_2_ CH_2_), 41.3 (NCH_2_), 110.7 (CH), 120.0 (CH), 122.2 (CH), 122.8 (CH), 127.0 (CH), 128.9 (2xCH), 129.1 (2xCH), 133.9 (C), 139.6 (C), 143.8 (C), 144.6 (CH-imidazole), 163.9 (C), 165.7 (C). HRMS (ESI) *m/z* Calculated: 350.1201 [M + H]^+^, Found: 350.1211 [M + H]^+^.

*2- {[2-(1H-benzo[d]imidazol-1-yl)ethyl]-5- [(4-chlorophenethyl)thio]}-1,3,4-oxadiazole* **(6b).**

Prepared from 5-(2-(1*H*-benzo[*d*]imidazol-1-yl)ethyl)-1,3,4-oxadiazole-2-thiol (**4**) (0.2 g, 0.813 mmol) and 4-chlorophenethyl bromide (0.196 g, 0.89 mmol). Purified using gradient chromatography eluting with 100% EtOAc to afford the product as a white solid: Yield: 0.203 g (65%); mp 90–92 °C; TLC: CH_2_Cl_2_-MeOH 9:1 v/v, R_f_ 0.57; HPLC: 100% at R.T. 4.50 min. ^1^H NMR (DMSO-*d*_*6*_) δ: 2.96 (t, *J* = 7.4 Hz, 2H, SCH_2_CH_2_), CH_2_ signal is obscured by H_2_O in DMSO-*d*_*6*_ signal, 3.44 (t, *J* = 6.7 Hz, 2H, SCH_2_), 4.68 (t, *J* = 6.7 Hz, 2H, NCH_2_), 7.22 (m, 4H, Ar), 7.35 (d, *J* = 8.4 Hz, 2H, Ar), 7.58 (d, *J* = 8.1 Hz, 1H, Ar), 7.63 (d, *J* = 7.8 Hz, 1H, Ar), 8.22 (s, 1H, CH-imidazole). ^13^C NMR (DMSO-*d*_*6*_) δ: 26.2 (NCH_2_CH_2_), 33.3 (SCH_2_), 34.5 (SCH_2_ CH_2_), 41.3 (NCH_2_), 110.7 (CH), 120.0 (CH), 122.1 (CH), 122.9 (CH), 128.8 (2xCH), 131.0 (2xCH), 131.7 (C), 133.9 (C), 138.6 (C), 143.8 (C), 144.6 (CH-imidazole), 163.8 (C), 165.8 (C). HRMS (ESI) *m/z* Calculated: 384.0812 [M + H]^+^, Found: 384.0820 [M + H]^+^.

*2- {[2-(1H-benzo[d]imidazol-1-yl)ethyl]-5- [(4-methoxyphenethyl)thio]}-1,3,4-oxadiazole* **(6c).**

Prepared from 5-(2-(1*H*-benzo[*d*]imidazol-1-yl)ethyl)-1,3,4-oxadiazole-2-thiol (**4**) (0.2 g, 0.813 mmol) and 4-methoxyphenethyl bromide (0.19 g, 0.89 mmol). Purified using gradient chromatography eluting with 100% EtOAc to afford the product as a white solid: Yield: 0.2 g (75%); mp 65–67 °C; TLC: CH_2_Cl_2_-MeOH 9:1 v/v, R_f_ 0.57; HPLC: 100% at R.T. 4.34 min. ^1^H NMR (DMSO-*d*_*6*_) δ: 2.89 (t, *J* = 6.8 Hz, 2H, SCH_2_CH_2_), CH_2_ signal is obscured by H_2_O in DMSO-*d*_*6*_ signal, 3.44 (t, *J* = 6.8 Hz, 2H, SCH_2_), 3.73 (s, 3H, OCH_3_), 4.69 (t, *J* = 6.7 Hz, 2H, NCH_2_), 6.85 (d, *J* = 8.7 Hz, 2H, Ar), 7.13 (d, *J* = 8.7 Hz, 2H, Ar), 7.20 (td, *J* = 1.2, 7.3 Hz, 1H, Ar), 7.25 (td, *J* = 1.2, 8.1 Hz, 1H, Ar), 7.59 (d, *J* = 7.9 Hz, 1H, Ar), 7.63 (d, *J* = 7.7 Hz, 1, 1H, Ar), 8.22 (s, 1H, imidazole). ^13^C NMR (DMSO-*d*_*6*_ δ: 26.2 (NCH_2_CH_2_), 33.9 (SCH_2_), 34.4 (SCH_2_ CH_2_), 41.3 (NCH_2_), 55.9 (OCH_3_), 110.7 (CH), 114.3 (2xCH), 120.0 (CH), 122.1 (CH), 122.9 (CH), 130.1 (2xCH), 131.5 (C), 140.0 (C), 143.8 (C), 144.6 (CH-imidazole), 158.4 (C), 164.0 (C), 165.7 (C). HRMS (ESI) *m/z* Calculated: 380.130 [M + H]^+^, Found: 380.1320 [M + H]^+^.

*2- {[2-(1H-benzo[d]imidazol-1-yl)ethyl]-5-(benzylthio)}-1,3,4-thiadiazole* **(8a).**

Prepared from 5-(2-(1*H*-benzo[*d*]imidazol-1-yl)ethyl)-1,3,4-thiadiazole-2-thiol (**7**) (0.2 g, 0.76 mmol) and benzyl chloride (0.11 g, 0.84 mmol), and purified using gradient chromatography eluting with CH_2_Cl_2_-MeOH 97.5:2.5 v/v to afford the as a white solid: Yield: 0.167 g (62%); mp 88–90 °C; TLC: CH_2_Cl_2_-MeOH 9:1 v/v, R_f_ 0.5; HPLC: 100% at R.T. 4.24 min. ^1^H NMR (DMSO-*d*_*6*_) δ: 3.44 (t, *J* = 6.8 Hz, 2H, NCH_2_CH_2_), 4.40 (s, 2H, SCH_2_), 4.67 (t, *J* = 6.7 Hz, 2H, NCH_2_), 7.19–7.34 (m, 5H, Ar), 7.37 (m, 2H, Ar), 7.57 (m, 1H, Ar), 7.64 (m, 1H, Ar), 8.19 (s, 1H, imidazole). ^13^C NMR (DMSO-*d*_*6*_) δ: 26.2 (NCH_2_CH_2_), 36.1 (SCH_2_), 41.3 (NCH_2_), 110.7 (CH), 120.0 (CH), 122.1 (CH), 122.9 (CH), 128.2 (CH), 129.0 (2xCH), 129.4 (2xCH), 133.9 (C), 136.9 (C), 143.8 (C), 144.5 (CH-imidazole), 163.6 (C), 165.9 (C). HRMS (ESI): Calculated: 352.0816 [M + H]^+^, Found: 352.0850 [M + H]^+^.

*2- {[2-(1H-benzo[d]imidazol-1-yl)ethyl]-5- [(2,4-dichlorobenzyl)thio]} -1,3,4-thiadiazole* **(8b).**

Prepared from 5-(2-(1*H*-benzo[*d*]imidazol-1-yl)ethyl)-1,3,4-thiadiazole-2-thiol (**7**) (0.2 g, 0.76 mmol) and 2,4-dichlorobenzyl chloride (0.16 g, 0.84 mmol). Purified using gradient chromatography eluting with CH_2_Cl_2_-MeOH 96:4 v/v to afford the product as a white solid: Yield: 0.22 g (69%); mp 84–86 °C; TLC: CH_2_Cl_2_-MeOH 9:1 v/v, R_f_ 0.55; HPLC: 100% at R.T. 4.60 min; ^1^H NMR (DMSO-*d*_*6*_) δ: 3.44 (t, *J* = 6.7 Hz, 2H, NCH_2_CH_2_), 4.47 (s, 2H, SCH_2_), 4.67 (t, *J* = 6.7 Hz, 2H, NCH_2_), 7.21 (m, 2H, Ar), 7.37 (dd, *J* = 8.3 Hz, 2.2 Hz, 1H, Ar), 7.49 (d, *J* = 8.4 Hz, 1H, Ar), 7.56 (m, 1H, Ar), 7.63 (m, 1H, Ar), 7.66 (d, *J* = 2.2 Hz, 1H, Ar), 8.21 (s, 1H, imidazole). ^13^C NMR (DMSO-*d*_*6*_) δ: 26.3 (NCH_2_CH_2_), 33.9 (SCH_2_), 41.2 (NCH_2_), 110.6 (CH), 120.0 (CH), 122.1 (CH), 122.9 (CH), 128.0 (CH), 129.6 (CH), 133.2 (CH), 133.5 (C), 133.9 (C), 134.0 (C), 134.7 (C), 143.8 (C), 144.6 (CH-imidazole), 162.9 (C), 166.2 (C). HRMS (ESI) *m/z* Calculated: 420.0037 [M + H]^+^, Found: 420.0035 [M + H]^+^.

*2- {[2-(1H-benzo[d]imidazol-1-yl)ethyl]-5- [(4-fluorobenzyl)thio]} -1,3,4-thiadiazole* **(8c).**

Prepared from 5-(2-(1*H*-benzo[*d*]imidazol-1-yl)ethyl)-1,3,4-thiadiazole-2-thiol (**7**) (0.2 g, 0.76 mmol) and 4-flourobenzyl chloride (0.12 g, 0.84 mmol). Purified using gradient chromatography eluting with 100% EtOAc to afford the product as a white solid: Yield: 0.16 g (71%); mp 76–78 °C; TLC: CH_2_Cl_2_-MeOH 9:1 v/v, R_f_ 0.62; HPLC: 100% at R.T. 4.27 min. ^1^H NMR (DMSO-*d*_*6*_) δ: 3.44 (t, *J* = 6.8 Hz, 2H, NCH_2_CH_2_), 4.40 (s, 2H, SCH_2_), 4.67 (t, *J* = 6.7 Hz, 2H, NCH_2_), 7.14 (t, *J* = 8.9 Hz, 2H, Ar), 7.22 (m, 2H, Ar), 7.41 (m, 2H, Ar), 7.56 (m, 1H, Ar), 7.64 (m, 1H, Ar), 8.19 (s, 1H, Imidazole). ^13^C NMR (DMSO-*d*_*6*_) δ: 26.2 (NCH_2_CH_2_), 35.3 (SCH_2_), 41.2 (NCH_2_), 110.7 (CH), 115.7 (CH), 115.9 (CH), 119.9 (CH), 122.1 (CH), 122.9 (CH), 131.5 (CH), 131.6 (CH), 131.3 (C), 133.9 (C), 143.8 (C), 144.6 (CH-imidazole), 161.1 (C), 163.0 (C), 163.5 (C), 165.9 (C). HRMS (ESI) m/z Calculated: 370.0722 [M + H]^+^, Found: 370.0717 [M + H]^+^.

*4-{[( 5- [2-(1H-benzo[d]imidazol-1-yl)ethyl]-1,3,4-thiadiazole-2-yl}thio)methyl] benzonitrile* **(8d).**

Prepared from 5-(2-(1*H*-benzo[*d*]imidazol-1-yl)ethyl)-1,3,4-thiadiazole-2-thiol (**7**) (0.1 g, 0.38 mmol) and 4-(chloromethyl)benzonitrile (0.07 g, 0.42 mmol). Purified using gradient chromatography eluting with 100% EtOAc + 1% Et_3_N to afford the product as a white solid: Yield: 0.17 gm, 61%; mp 112–114 °C; TLC: CH_2_Cl_2_-MeOH 9:1 v/v, R_f_ 0.56; HPLC: 100% at R.T. 4.02 min. ^1^H NMR (DMSO-*d*_*6*_) δ: 3.43 (t, *J* = 6.8 Hz, 2H, NCH_2_CH_2_), 4.48 (s, 2H, SCH_2_), 4.66 (t, *J* = 6.7 Hz, 2H, NCH_2_), 7.21 (m, 2H, Ar), 7.57 (m, 3H, Ar), 7.64 (m, 1H, Ar), 7.77 (d, *J* = 8.4 Hz, 2H, Ar), 8.19 (s, 1H, Imidazole). ^13^C NMR (DMSO-*d*_*6*_) δ: 26.2 (NCH_2_CH_2_), 35.5 (SCH_2_), 41.2 (NCH_2_), 110.6 (CH), 110.9 (CN), 119.1 (C), 120.0 (CH), 122.1 (CH), 122.9 (CH), 130.4 (2xCH), 132.9 (2xCH), 133.9 (C), 143.2, (C), 143.8 (C), 144.6 (CH-imidazole), 163.2 (C), 166.1 (C). HRMS (ESI) *m/z* Calculated: 377.0769 [M + H]^+^, Found: 377.0752 [M + H]^+^.

*2- {[2-(1H-benzo[d]imidazol-1-yl)ethyl]-5-(phenethylthio)}-1,3,4-thiadiazole* **(9a).**

Prepared from 5-(2-(1*H*-benzo[*d*]imidazol-1-yl)ethyl)-1,3,4-thiadiazole-2-thiol (**7**) (0.1 g, 0.38 mmol) and 2-bromoethyl benzene (0.07 g, 0.42 mmol). Purified using gradient chromatography eluting with 100% EtOAc to afford the product as a white solid: Yield: 0.071 g (51%); mp 76–78 °C; TLC: CH_2_Cl_2_-MeOH 9:1 v/v, R_f_ 0.57; HPLC: 100% at R.T. 4.35 min. ^1^H NMR (DMSO-*d*_*6*_) δ: 2.97 (t, *J* = 7.3 Hz, 2H, SCH_2_CH_2_), 3.39 (t, *J* = 7.2 Hz, 2H, NCH_2_CH_2_), 3.44 (t, *J* = 6.7 Hz, 2H, SCH_2_), 4.69 (t, *J* = 6.7 Hz, 2H, NCH_2_), 7.19–7.31 (m, 7H, Ar), 7.59 (d, *J* = 7.8 Hz, 1H, Ar), 7.63 (d, *J* = 8.0 Hz, 1H, Ar), 8.22 (s, 1H, imidazole). ^13^CNMR (DMSO-*d*_*6*_) δ: 26.2 (NCH_2_CH_2_), 33.5 (SCH_2_), 35.3 (SCH_2_ CH_2_), 41.3 (NCH_2_), 110.7 (CH), 120.0 (CH), 122.1 (CH), 122.9 (CH), 127.0 (CH), 128.9 (2xCH), 129.1 (2xCH), 134.0 (C), 139.6 (C), 143.8 (C), 144.6 (CH-imidazole), 163.9 (C), 165.7 (C). HRMS (ESI) *m/z* Calculated: 366.0973 [M + H]^+^, Found: 366.0962 [M + H]^+^.

*2- {[2-(1H-benzo[d]imidazol-1-yl)ethyl]-5- [(4-chlorophenethyl)thio]}-1,3,4-thiadiazole* **(9b).**

Prepared from 5-(2-(1*H*-benzo[*d*]imidazol-1-yl)ethyl)-1,3,4-thiadiazole-2-thiol (**7**) (0.1 g, 0.38 mmol) and 4-chlorophenethyl bromide (0.092 g, 0.42 mmol). Purified using gradient chromatography eluting with 100% EtOAc to afford the product as a white solid: Yield: 0.06 g (40%); mp 96–98 °C; TLC: CH_2_Cl_2_-MeOH 9:1 v/v, R_f_ 0.60; HPLC: 100% at R.T. 4.49 min. ^1^H NMR (DMSO-*d*_*6*_) δ: 2.96 (t, *J* = 7.2 Hz, 2H, SCH_2_CH_2_), 3.38 (t, *J* = 7.1 Hz, 2H, NCH_2_CH_2_), 3.44 (t, *J* = 6.8 Hz, 2H, SCH_2_), 4.69 (t, *J* = 6.7 Hz, 2H, NCH_2_), 7.20 (m, 1H, Ar), 7.25 (m, 3H, Ar), 7.35 (d, *J* = 8.5 Hz, 2H, Ar), 7.59 (d, *J* = 7.7 Hz, 1H, Ar), 7.63 (d, *J* = 7.8 Hz, 1H, Ar), 8.22 (s, 1H, imidazole). ^13^C NMR (DMSO-*d*_*6*_) δ: 26.2 (NCH_2_CH_2_), 33.3 (SCH_2_), 34.5 (SCH_2_ CH_2_), 41.3 (NCH_2_), 110.7 (CH), 120.0 (CH), 122.1 (CH), 122.9 (CH), 128.8 (2xCH), 131.0 (2xCH), 131.7 (C), 134.0 (C), 138.6 (C), 143.8 (C), 144.6 (CH-imidazole), 163.8 (C), 165.8 (C). HRMS (ESI) *m/z* Calculated: 400.0583 [M + H]^+^, Found: 400.0555 [M + H]^+^.

*2- {[2-(1H-benzo[d]imidazol-1-yl)ethyl]-5- [(4-methoxyphenethyl)thio]}-1,3,4-thiadiazole* **(9c).**

Prepared from 5-(2-(1*H*-benzo[*d*]imidazol-1-yl)ethyl)-1,3,4-thiadiazole-2-thiol (**7**) (0.1 g, 0.38 mmol) and 4-methoxyphenethyl bromide (0.09 g, 0.42 mmol). Purified using gradient chromatography eluting with 100% EtOAc to afford the product as a white solid: Yield: 0.166 g (55%); mp 68–70 °C; TLC: CH_2_Cl_2_-MeOH 9:1 v/v, R_f_ 0.66; HPLC: 100% at RT = 4.34 min. ^1^H NMR (DMSO-*d*_*6*_) δ: 2.89 (t, *J* = 7.1 Hz, 2H, SCH_2_CH_2_), CH_2_ signal is obscured by H_2_O in DMSO-*d*_*6*_ signal, 3.44 (t, *J* = 6.7 Hz, 2H, SCH_2_), 3.73 (s, 3H, OCH_3_), 4.69 (t, *J* = 6.7 Hz, 2H, NCH_2_), 6.85 (d, *J* = 8.7 Hz, 2H, Ar), 7.13 (d, *J* = 8.7 Hz, 2H, Ar), 7.20 (td, *J* = 1.2, 7.2 Hz, 1H, Ar), 7.25 (td, *J* = 1.2, 8.1 Hz, 1H, Ar), 7.59 (d, *J* = 7.8 Hz, 1H, Ar), 7.63 (d, *J* = 7.8 Hz, 1H, Ar), 8.22 (s, 1H, imidazole). ^13^C NMR (DMSO-*d*_*6*_) δ: 26.2 (NCH_2_CH_2_), 33.9 (SCH_2_), 34.4 (SCH_2_ CH_2_), 41.3 (NCH_2_), 55.5 (OCH_3_), 110.7 (CH), 114.3 (2xCH), 120.0 (CH), 122.1 (CH), 122.9 (CH), 130.1 (2xCH), 131.5 (C), 140.0 (C), 143.8 (C), 144.6 (CH-imidazole), 158.4 (C), 164.0 (C), 165.7 (C). HRMS (ESI): Calculated: 380.1307 [M + H]^+^, Found: 380.1319 [M + H]^+^.

### Computational studies

Flexible alignment studies were performed using MOE. 2015.10 software [31]. Flexible alignment was performed using MMFF94 forcefield, flexible alignment mode and the resulting conformations were examined according to their grand alignment score (S). The latter is the sum of the similarity measure of configuration (F) and the average strain energy of the molecules in the alignment in kcal/mol (U). The lower S value indicates better alignment. Multiple sequence alignment was performed using the EMBL-EBI (European Bioinformatics Institute) Job Dispatcher framework server, using Clustal Omega [33], while the amino acid sequences were imported in their FASTA formats from UniProtKB [47].

Docking studies were performed using MOE. 2015.10 software [48] using the crystal structure of *Th. thermophilus* PheRS [PDB ID: 1JJC] [32]. All minimisations were performed with MOE until a RMSD gradient of 0.01 kcal/mol/Å with Amber99 forcefield and automatic calculation of the partial charges. Docking settings were set as the following: Amber99 forcefield for result refinement, Triangle Matcher placement was chosen to determine the poses, London ΔG scoring function was applied for rescoring and result refinement, and the structures were further refined with rigid receptor refinement. The resulting docking poses were generated in a database, which was arranged according to the final score function (S) that is the score of the last stage which was not set to zero.

Molecular dynamic simulations were run using either the crystal structure of *Th. thermophilus* PheRS [PDB ID: 1JJC] [32] or *S. aureus* PheRS previously published homology model [34]. PDB files were first optimised using protein preparation wizard in Maestro [49] version 11.8.012 by assigning bond orders, adding hydrogen, and correcting incorrect bond types. A default quick relaxation protocol was used to minimise the MD systems with the Desmond programme [49]. In Desmond, the volume of space in which the simulation takes place, the global cell, is built up by regular 3D simulation boxes, which was utilised as part of this system for protein interactions. The simulation system was generated by embedding the protein model in an orthorhombic 10 Å water box. Overlapping water molecules were deleted. The solvated system was neutralised by adding sodium ions and salt concentration 0.15 M. Force-field parameters for the complexes were assigned using the OPLS_2005 forcefield, that is, a 200 ns molecular dynamic run in the NPT ensemble (T = 300 K) at a constant pressure of 1 bar. Energy and trajectory atomic coordinate data were recorded at each 1.2 ns.

Binding affinity (ΔG) calculations was performed using Prime/MMGBAS, available in the Schrödinger Prime suite, to calculate the binding free energy of **8b** complexed with the *S. aureus* homology model.

ΔG (bind) = E_complex (minimised)-(E_ligand (minimised) + E_receptor (minimised)).

The mean ΔG (bind) was calculated from each frame starting from the last 100 ns to the final frame of the MD simulation.

### Microbiological evaluation

Compounds **5a**-**d**, **6a**-**c**, **8a**-**d** and **9a**-**c** were evaluated using the guidelines of ISO20776 (the International Organization of Standardization) broth microdilution method using Muller-Hinton broth and concentration range of 0.008—128 μg/mL [50]. The MIC values were determined against *S. aureus* (ATCC 29,213), *E. faecalis* (ATCC 29,212), *P. aeruginosa* (ATCC 29,853), *E. coli* (ATCC 25,922) and *Klebsiella pneumoniae* (ATCC 700,603) using ciprofloxacin as a reference drug. The tests were performed in microdilution trays and the amount of growth in each well was compared with that in the positive growth control, and the recorded MIC is the lowest concentration of the agent that completely inhibits visible growth.

## Supplementary Information

**Additional file 1**. Contains: a- Fig S1. Figure illustrating the ^1^H NMR spectrum of compound 6b, b- The ^1^H NMR and ^13^C NMR charts of the target compounds and c- The HPLC traces of the target compounds.

## Data Availability

The datasets used or analysed during the current study are available from the corresponding author on reasonable request.

## Abbreviations

PheRS: Phenylalanyl-tRNA synthetase
aa-AMP: Aminoacyl adenylate
Phe-AMP: Phenylalanyl adenylate
MD: Molecular dynamics
MIC: Minimum inhibitory concentration
aaRSs: Aminoacyl tRNA synthetases
PheS: Phenylalanyl tRNA synthetase α subunit
PheT: Phenylalanyl tRNA synthetase β subunit
ATP: Adenosine triphosphate
PDB: Protein data bank
DMF: Dimethylformamide
MOE: Molecular operating environment
