# Supplementary material for: Design, computational studies, synthesis and in vitro antimicrobial evaluation of benzimidazole based thio-oxadiazole and thio-thiadiazole analogues
Source: BMC Chem. 2021 Oct 28;15(1):58. doi: 10.1186/s13065-021-00785-8 (PMC8555319; doi:10.1186/s13065-021-00785-8)
Supplement: Supplementary file 1 — Additional file 1. Contains: a- Fig S1. Figure illustrating the 1H NMR spectrum of compound 6b, b- The 1H NMR and 13C NMR charts of the target compounds and c- The HPLC traces of the target compounds. [file 13065_2021_785_MOESM1_ESM.docx]

Design, computational studies, synthesis and *in vitro* antimicrobial evaluation of benzimidazole based thio-oxadiazole and thio-thiadiazole analogues

Nada A. Noureldin^1,2*^, Jennifer Richards^3^, Hend Kothayer^2^, Mohammed M. Baraka^2^, Sobhy M. Eladl^2^, Mandy Wootton^3^, Claire Simons^1^

1 School of Pharmacy and Pharmaceutical Sciences, Cardiff University, Cardiff CF10 3NB, United Kingdom

2 Department of Medicinal Chemistry, Faculty of Pharmacy, Zagazig University, Zagazig P.C. 44519, Egypt

3 Specialist Antimicrobial Chemotherapy Unit, University Hospital of Wales, Heath Park, Cardiff CF14 4XW, United Kingdom

*Correspondence: [NANoureddine@pharmacy.zu.edu.eg](mailto:NANoureddine@pharmacy.zu.edu.eg)

^1^ School of Pharmacy and Pharmaceutical Sciences, Cardiff University, Cardiff CF10 3NB, United Kingdom

1. Department of Medicinal Chemistry, Faculty of Pharmacy, Zagazig University, Zagazig P.C. 44519, Egypt

**Supplementary material for** **^1^H NMR and ^13^C NMR of target compounds**

1. **A figure illustrating the ^1^H NMR spectrum of compound 6b:**


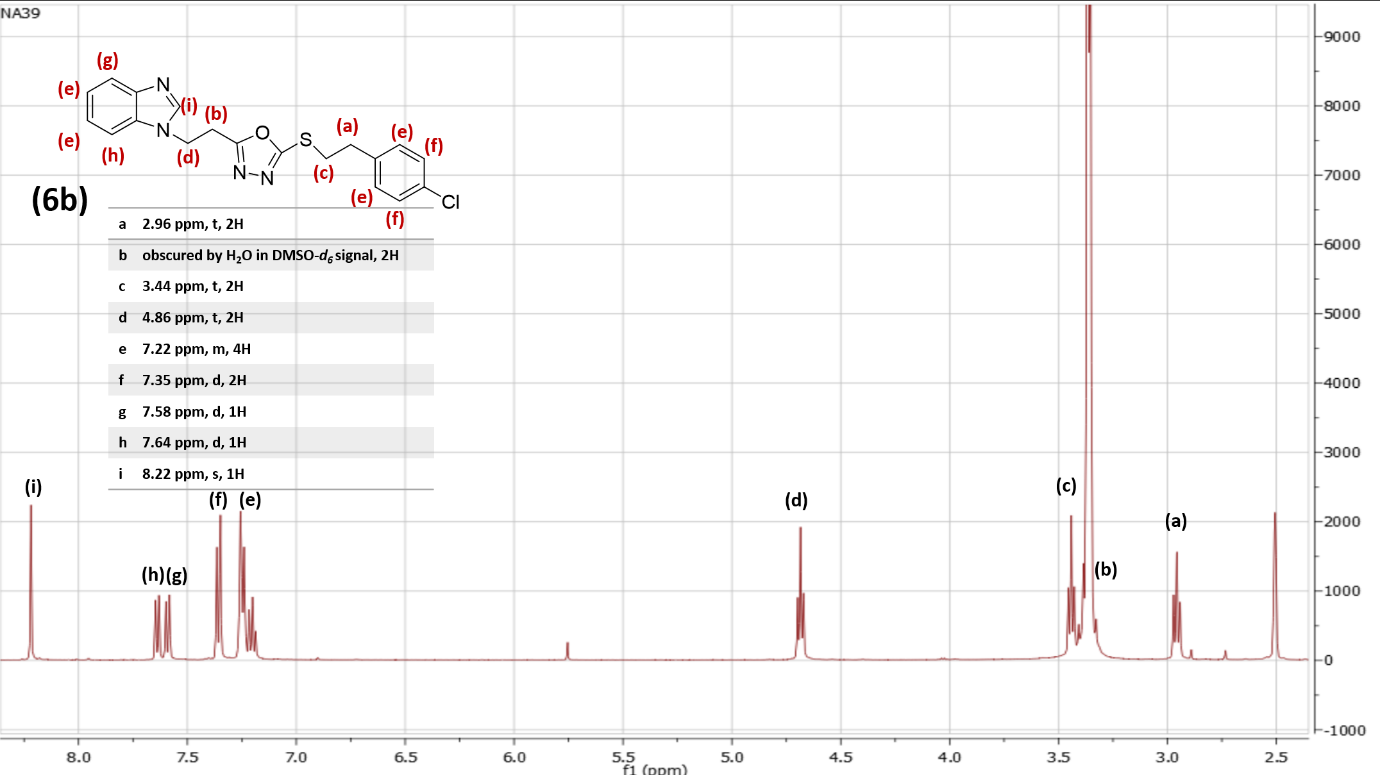
 **Fig S1. Figure illustrating the ^1^H NMR spectrum of compound 6b**

1. **^1^H NMR and ^13^C NMR charts of target compounds:**

**
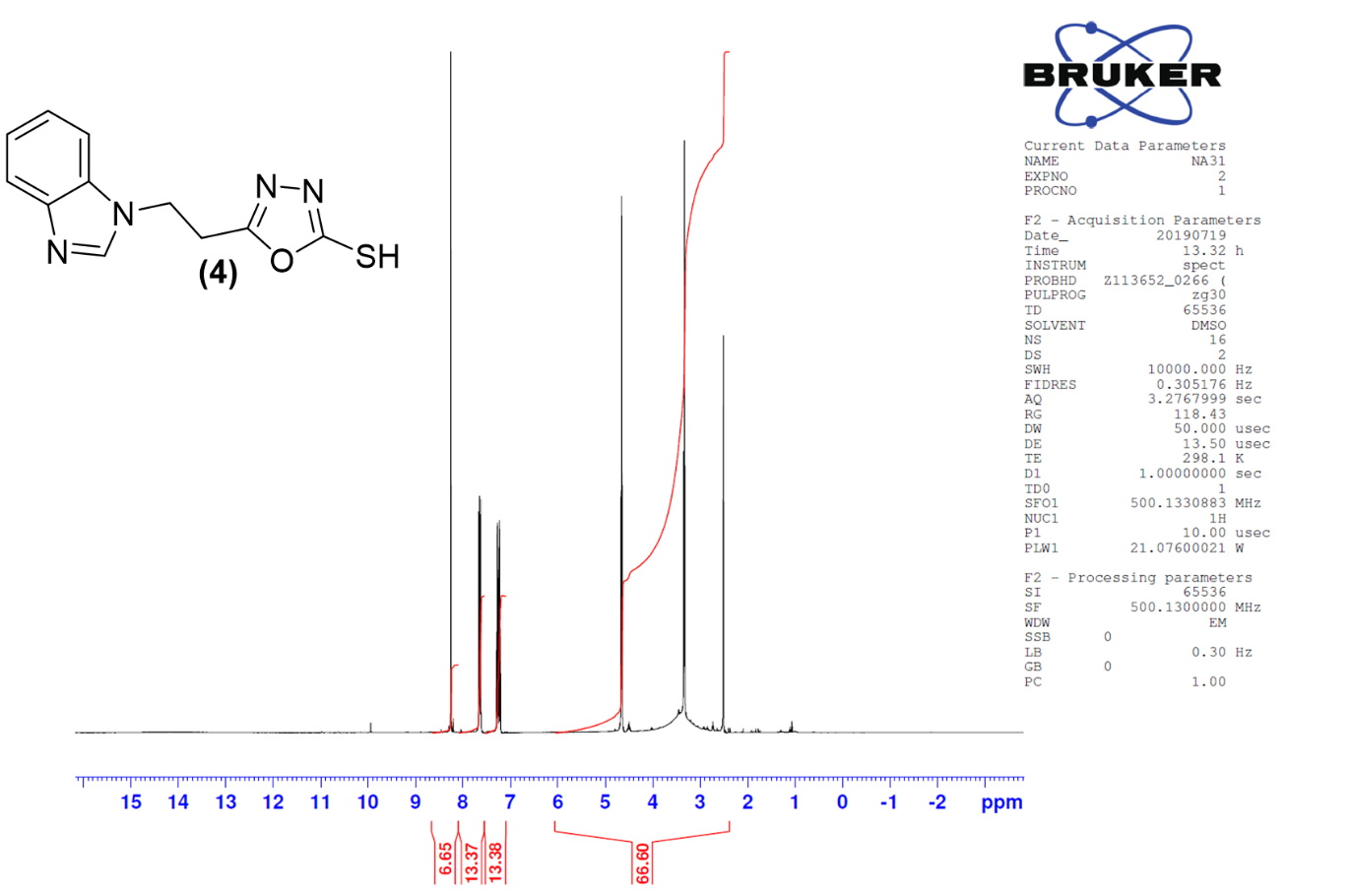
**


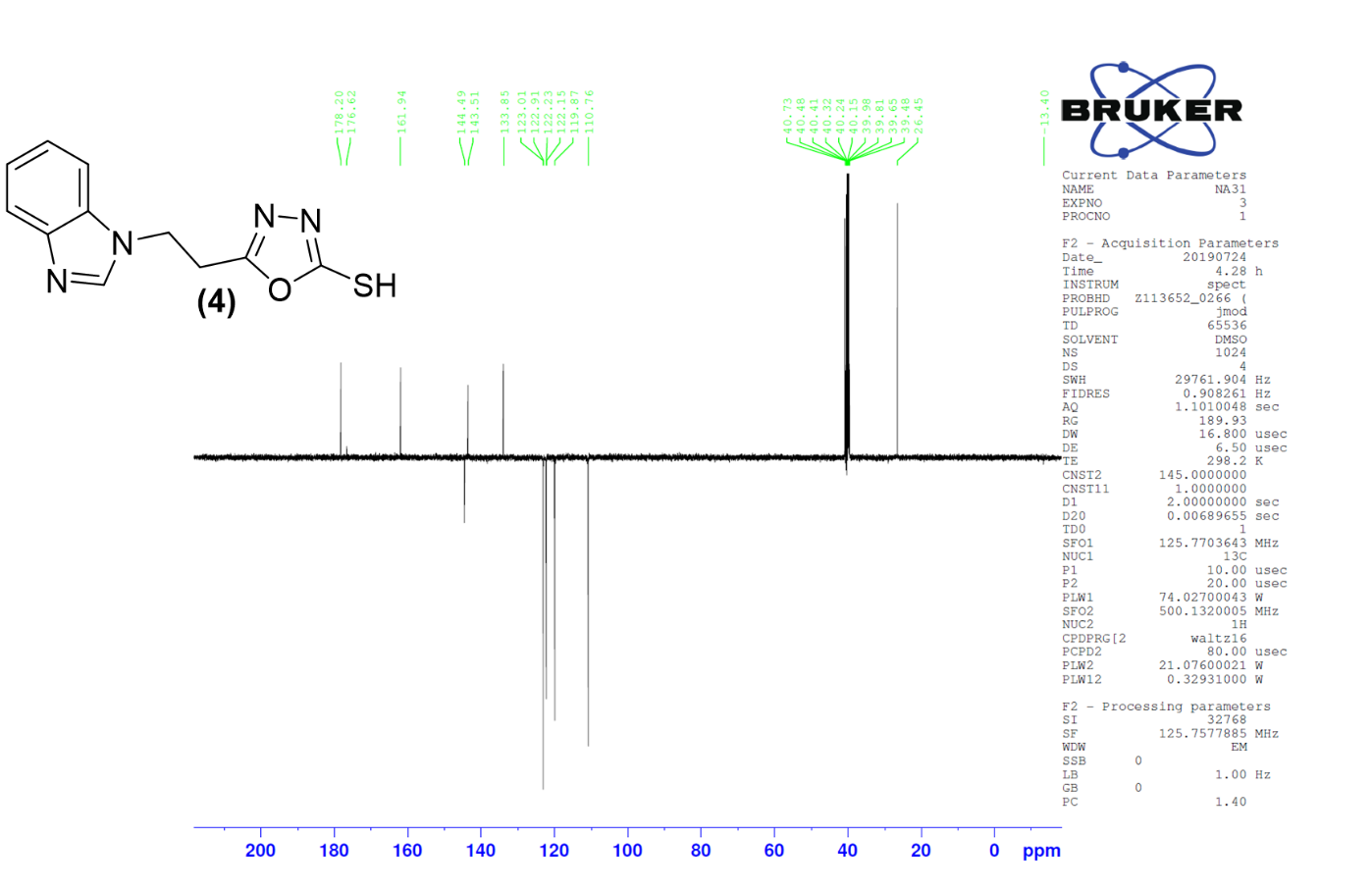


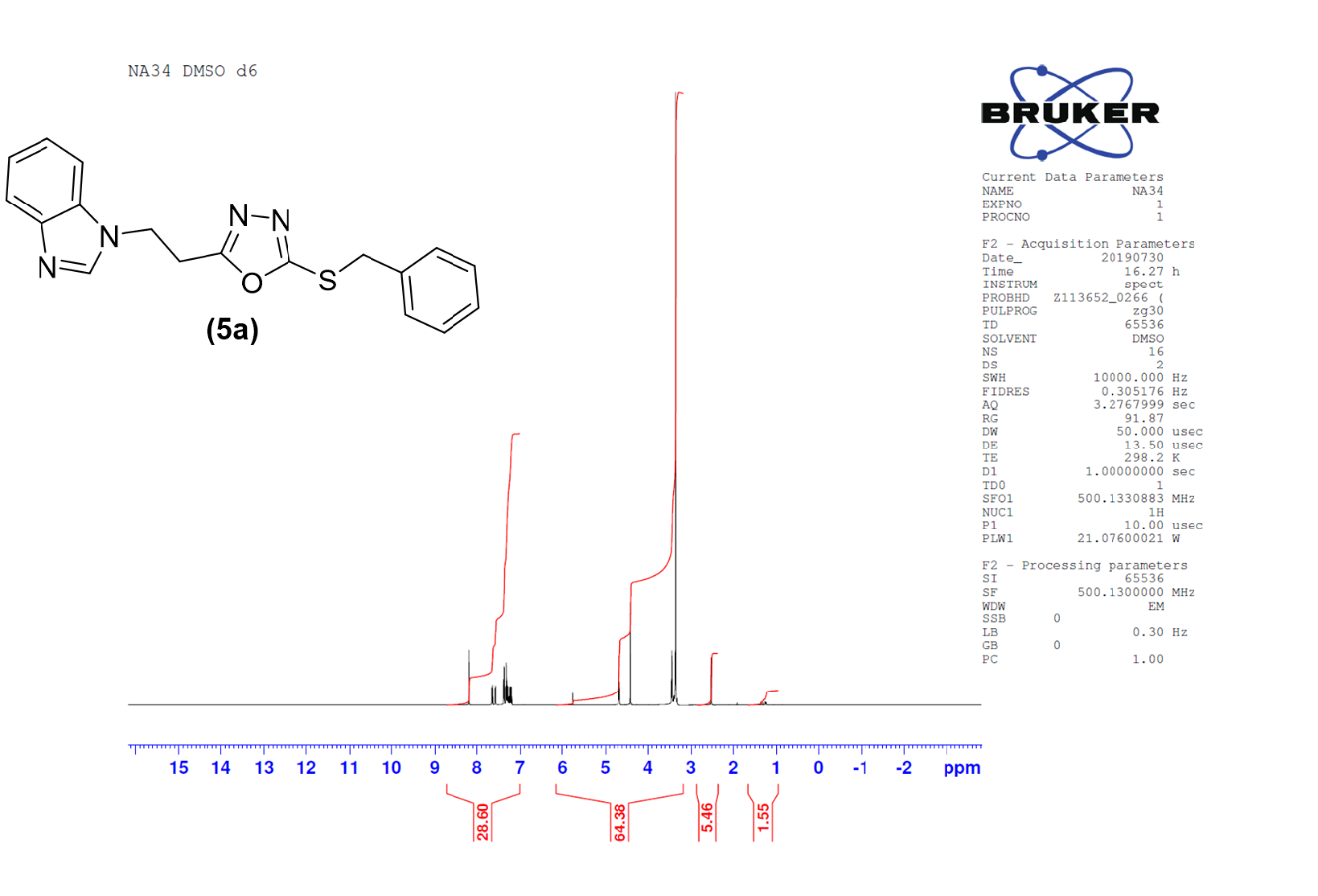


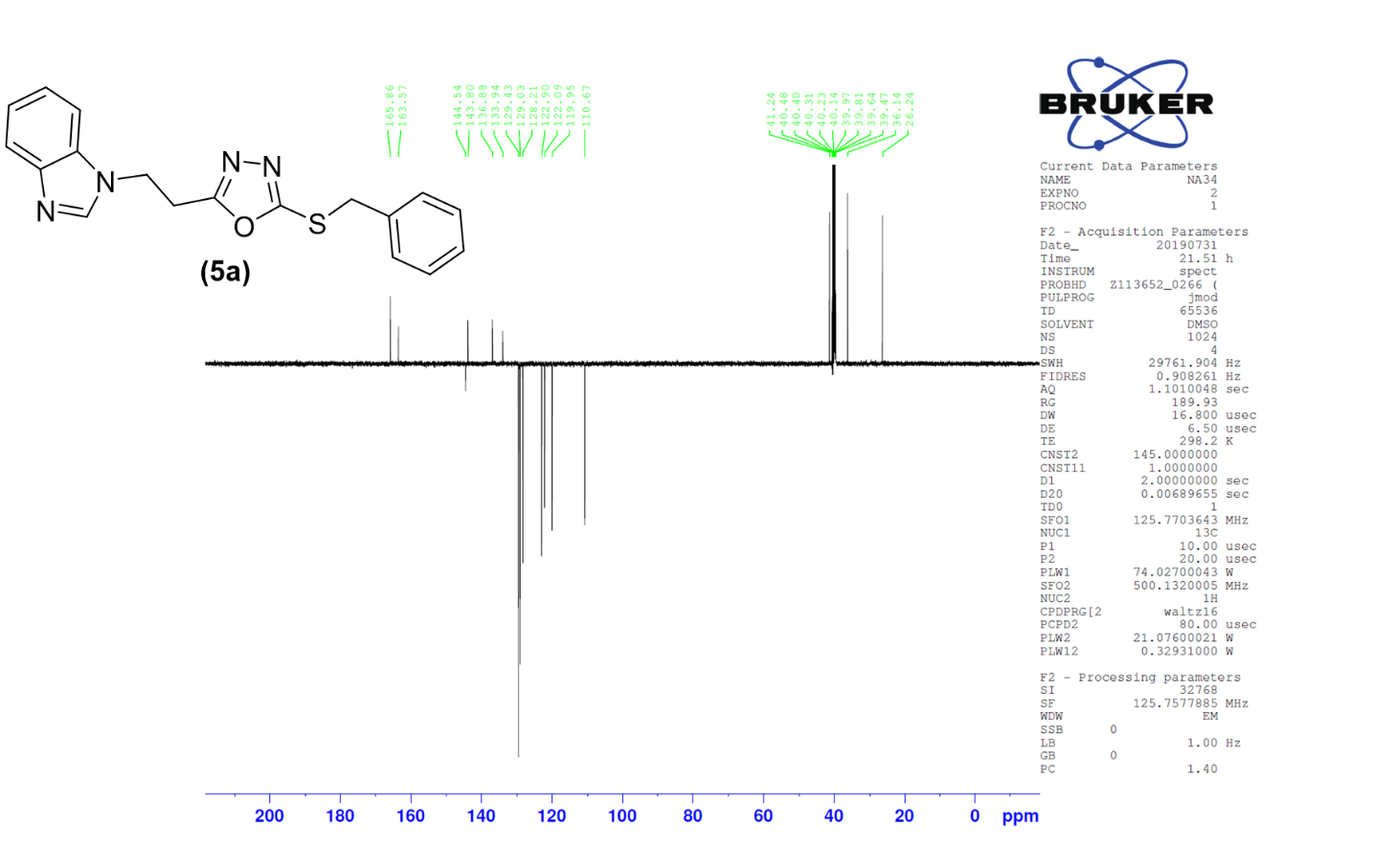


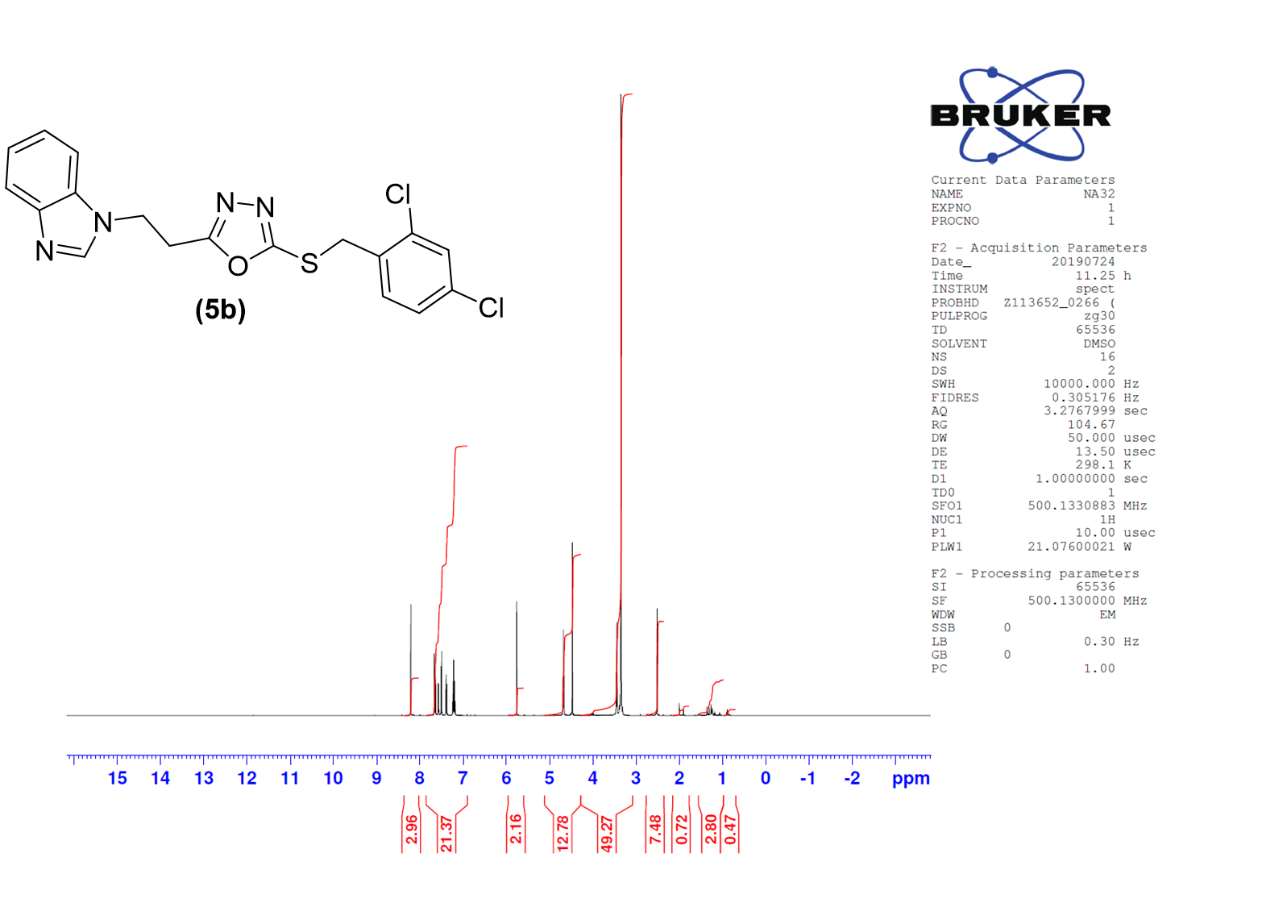

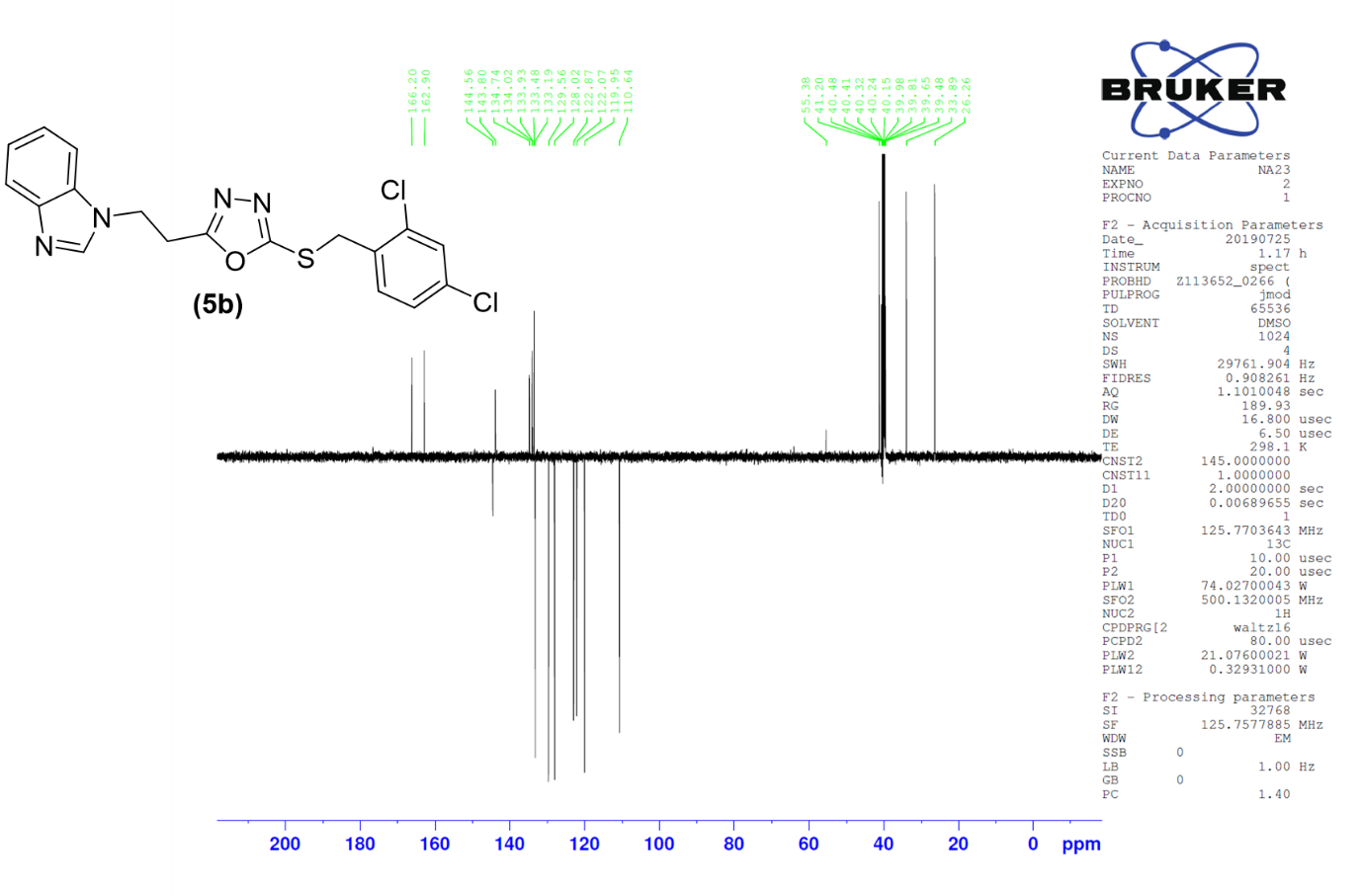


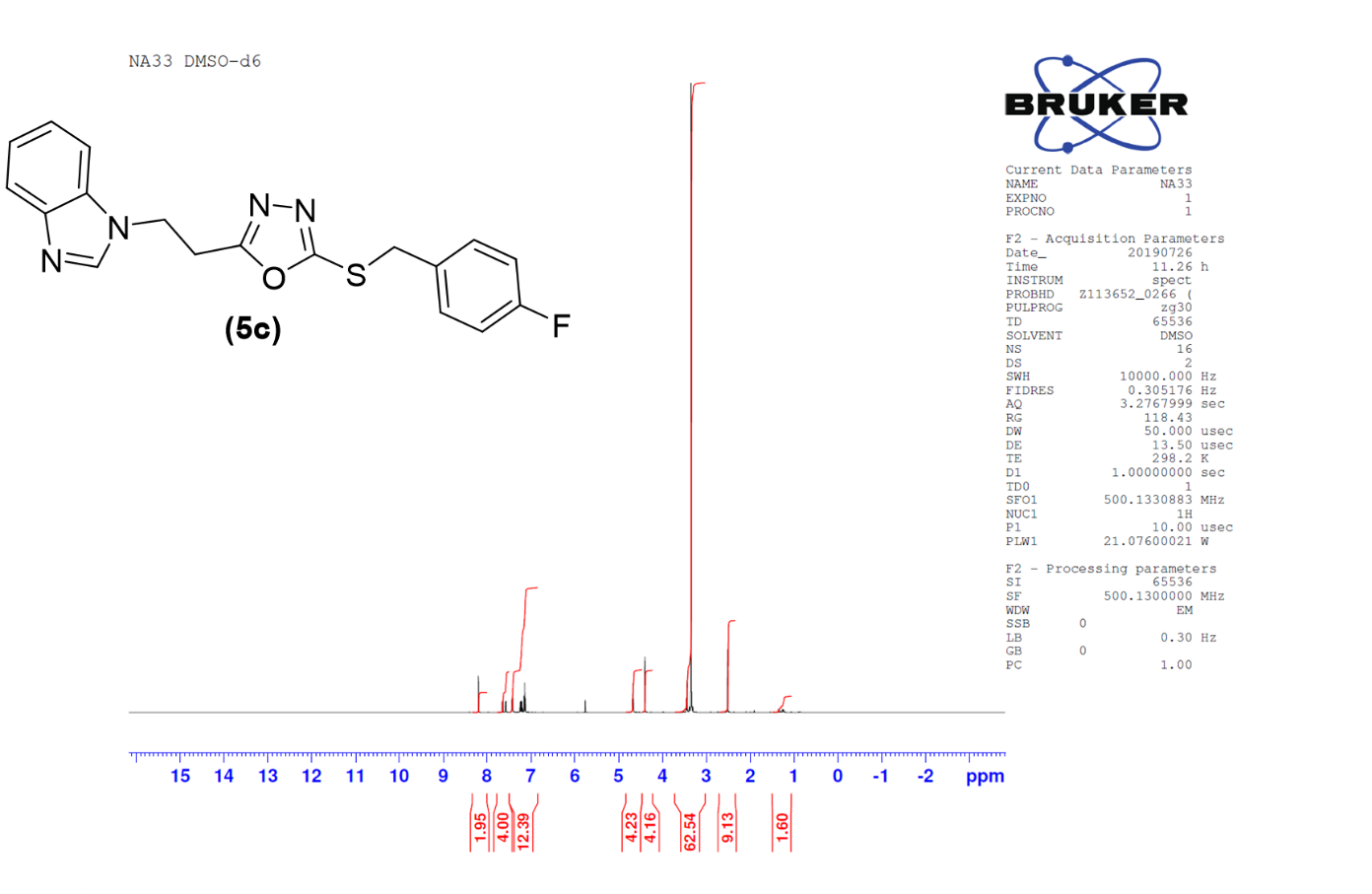


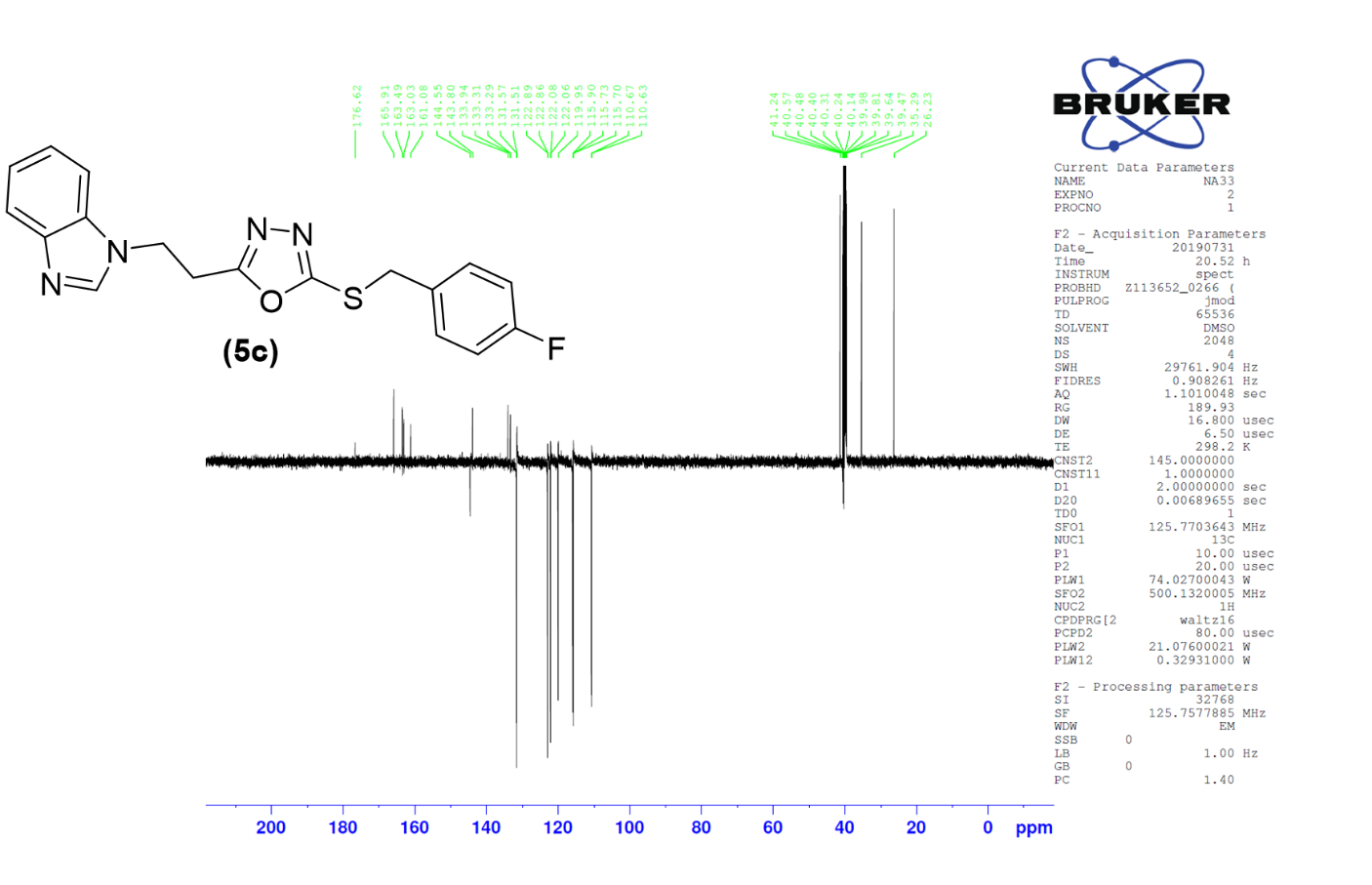


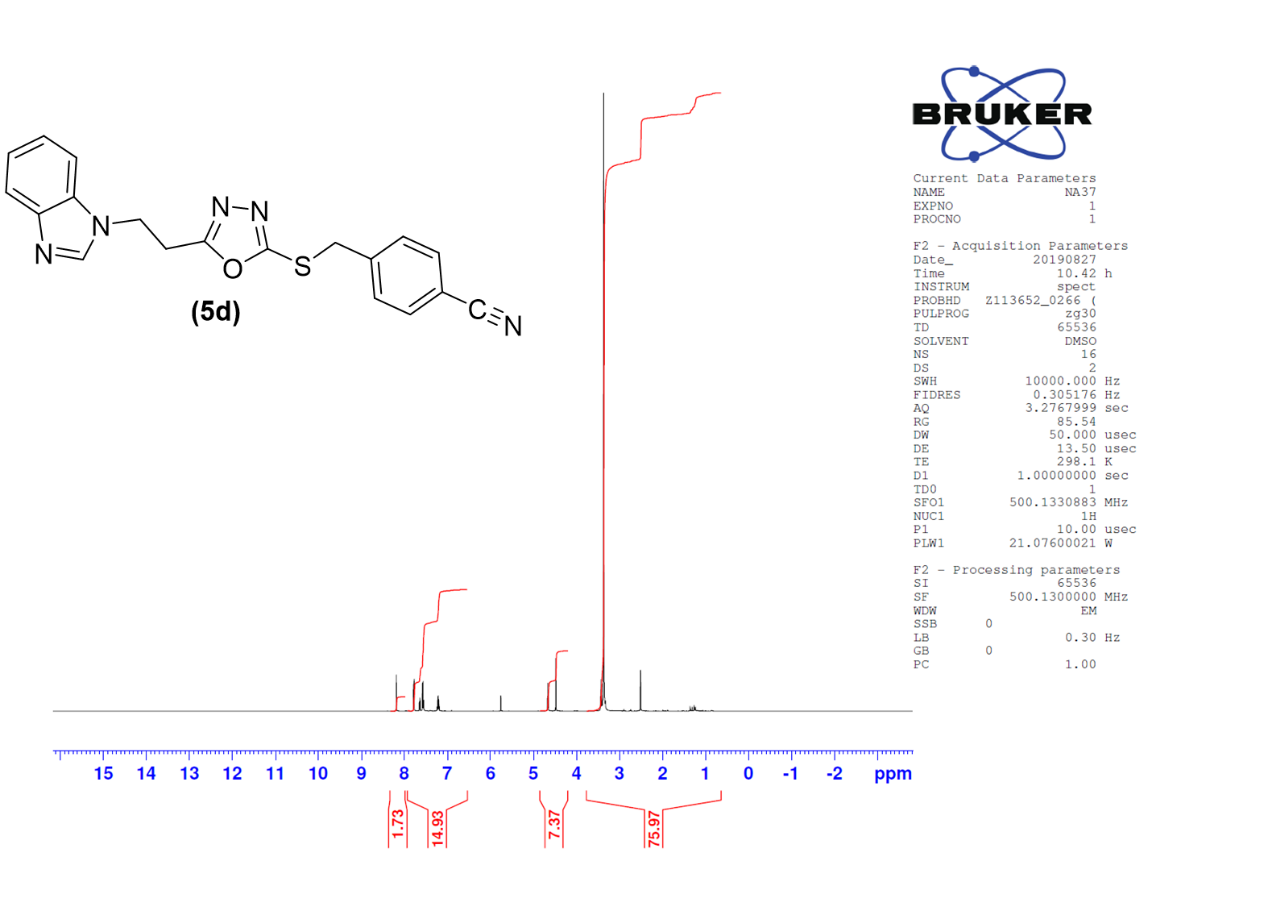


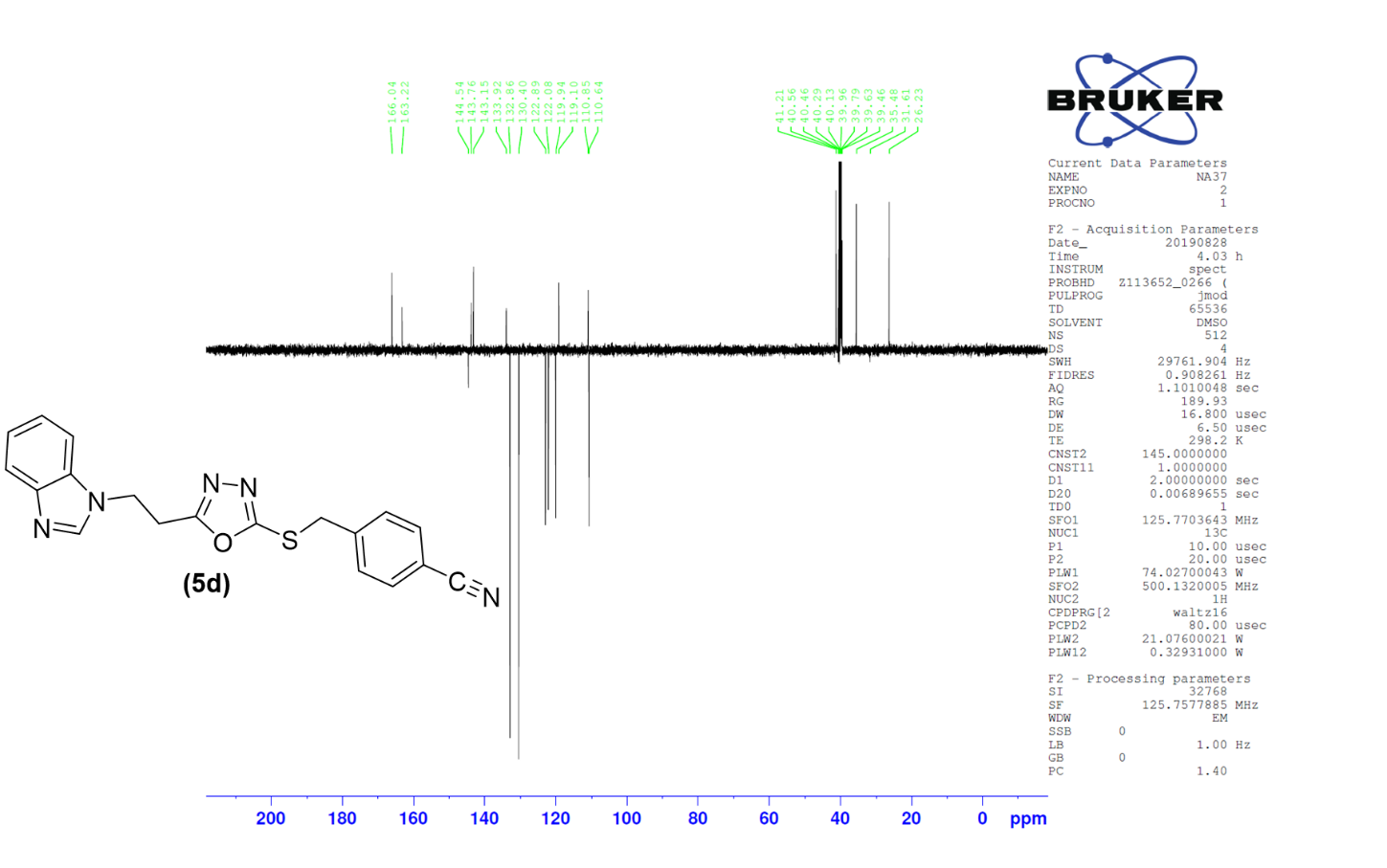


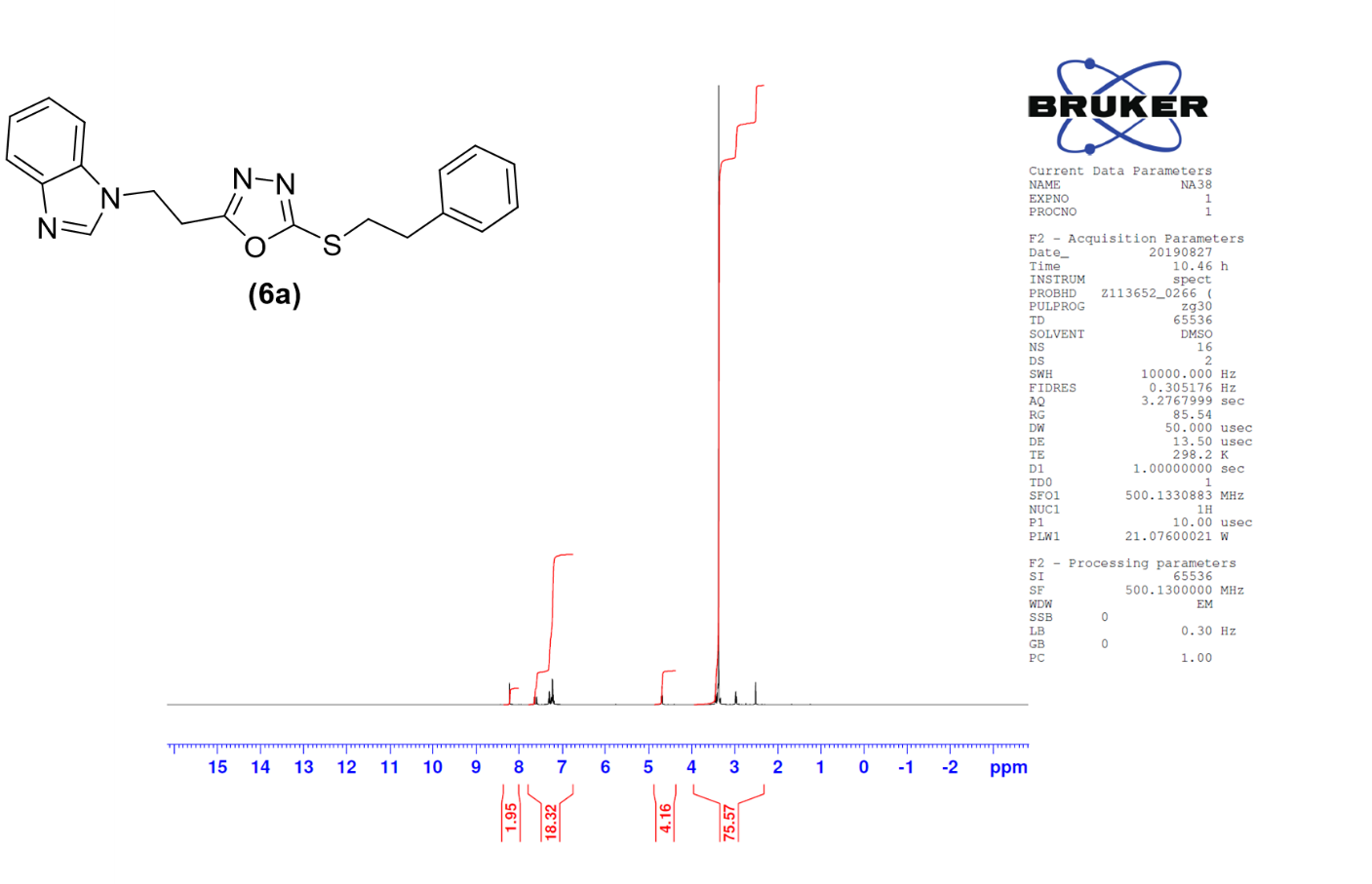


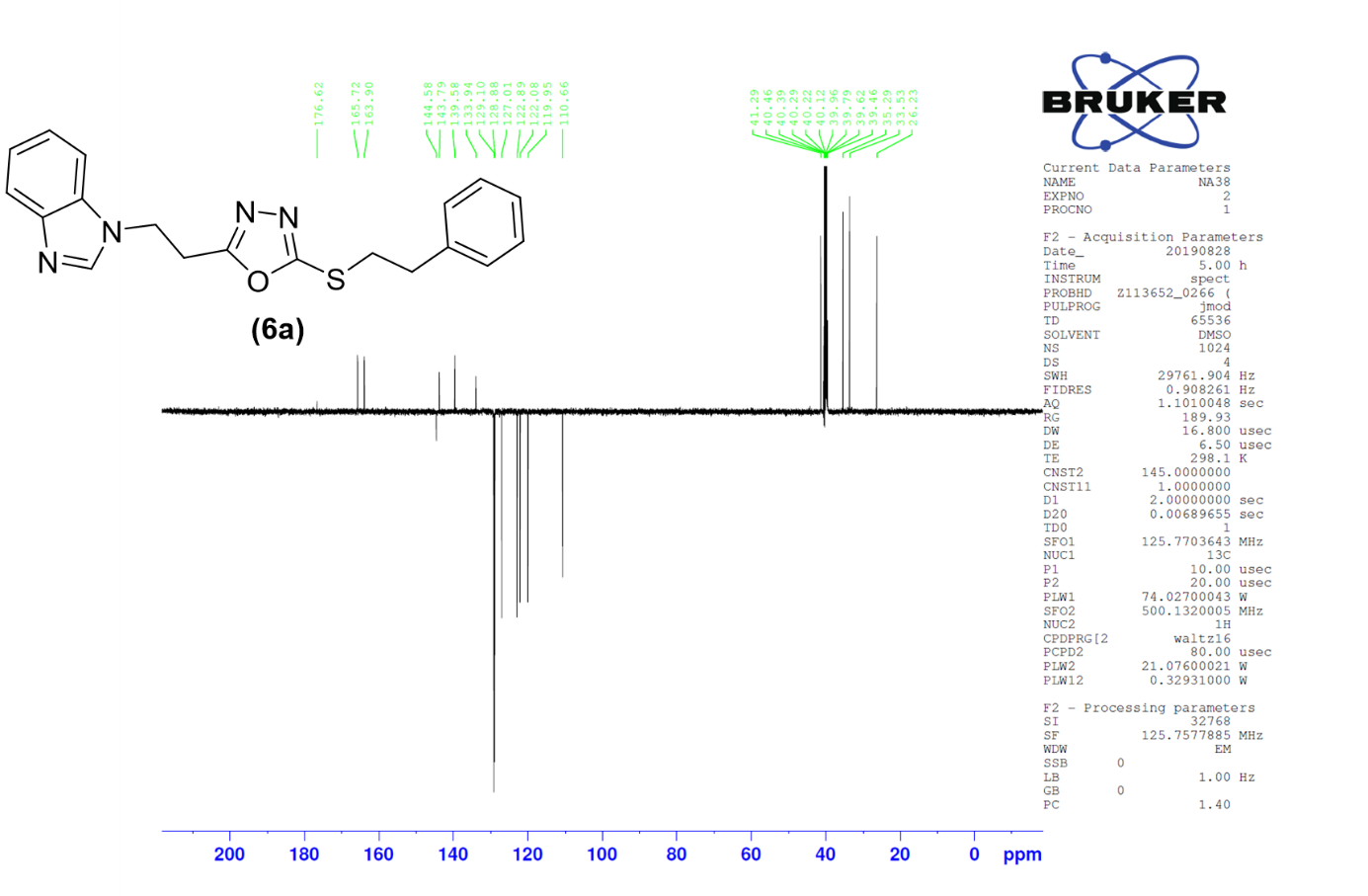


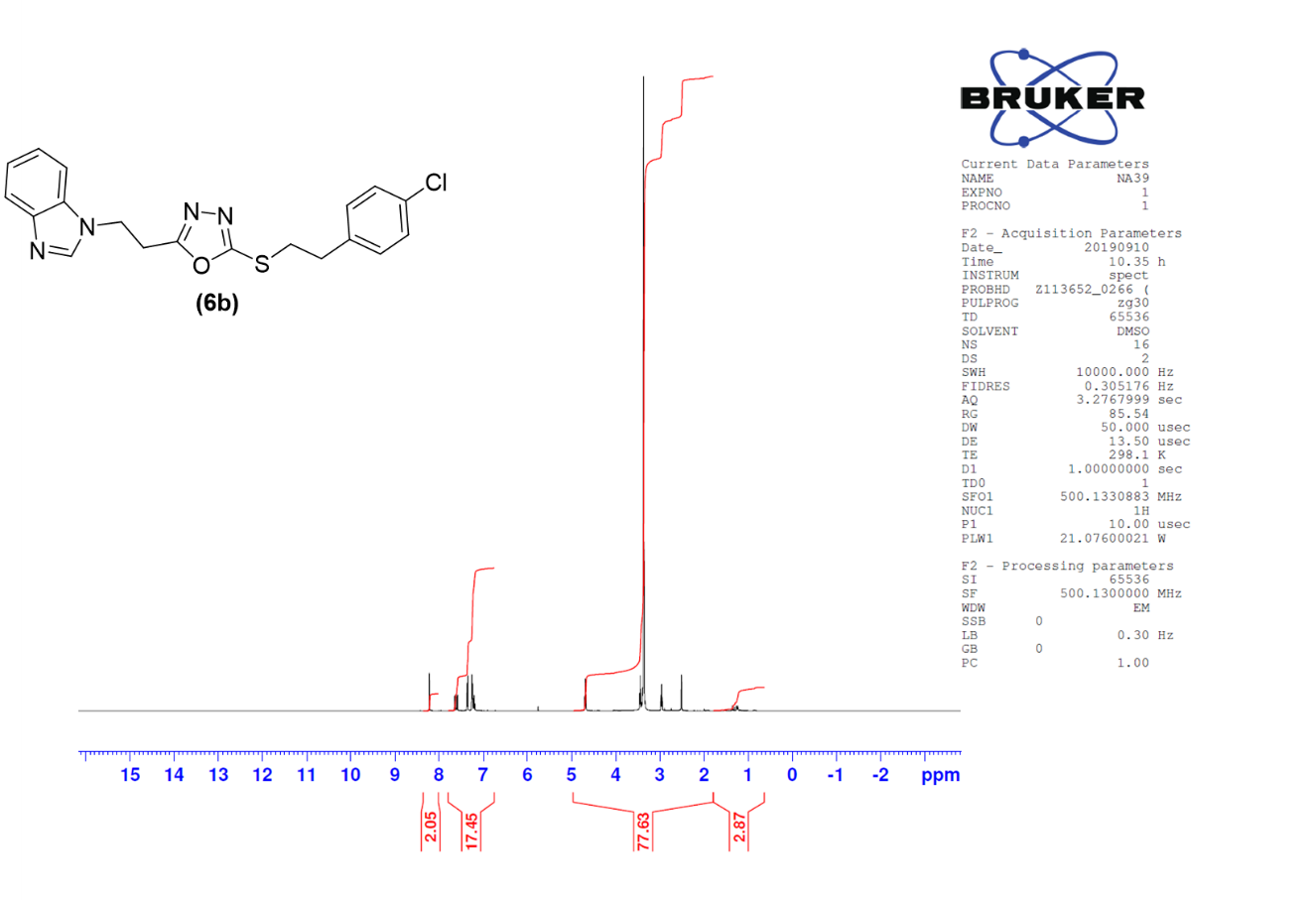


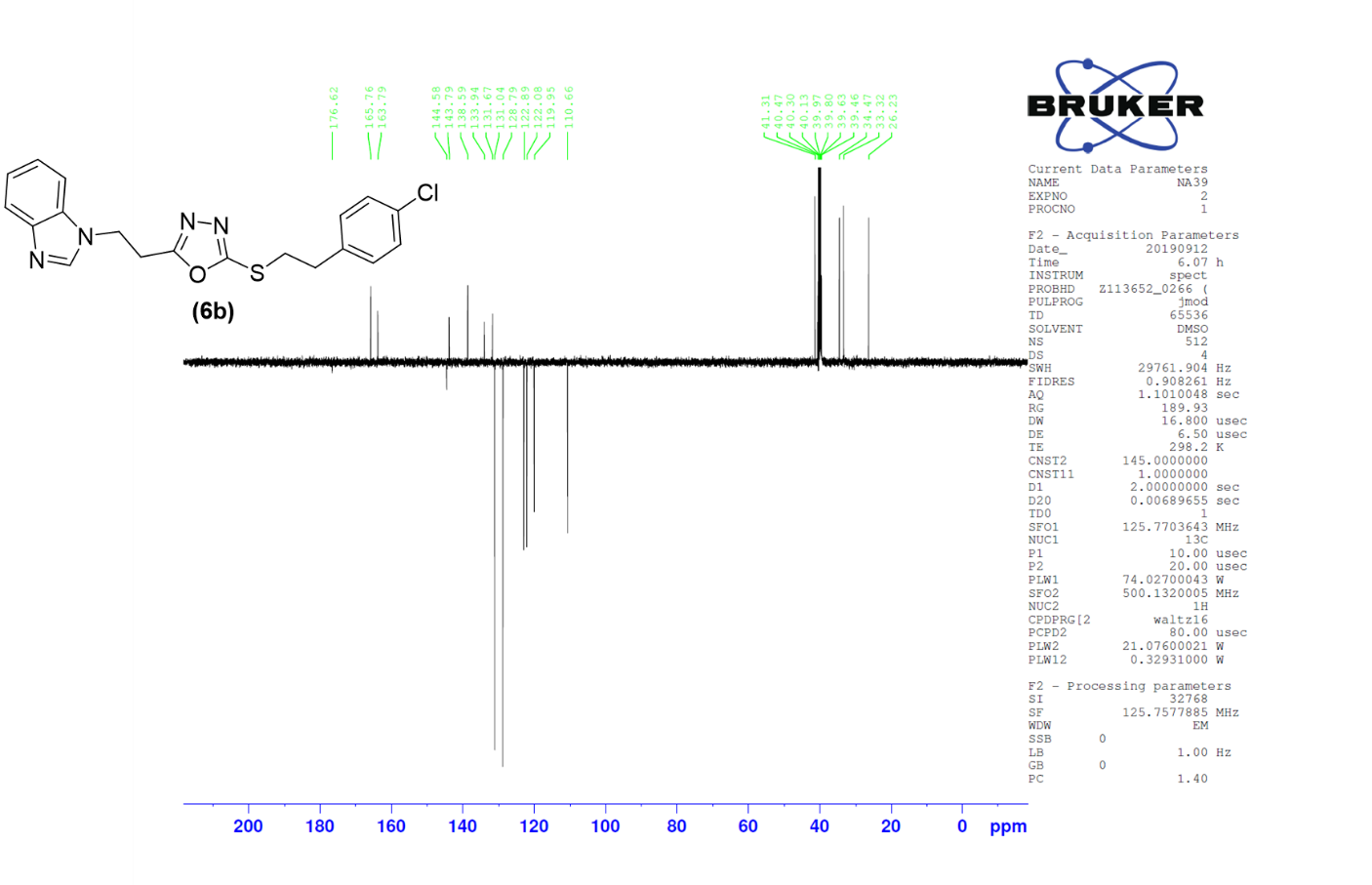


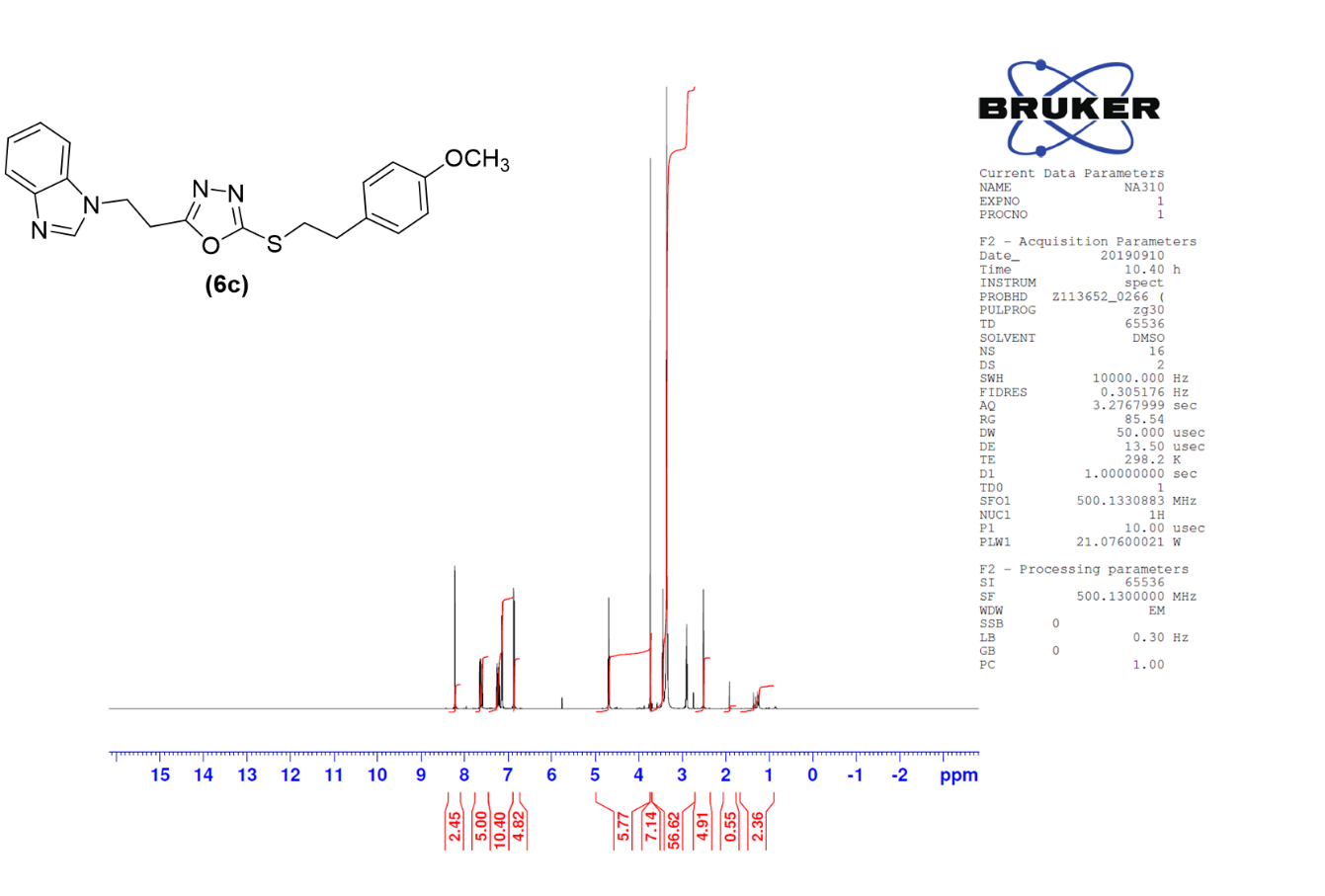


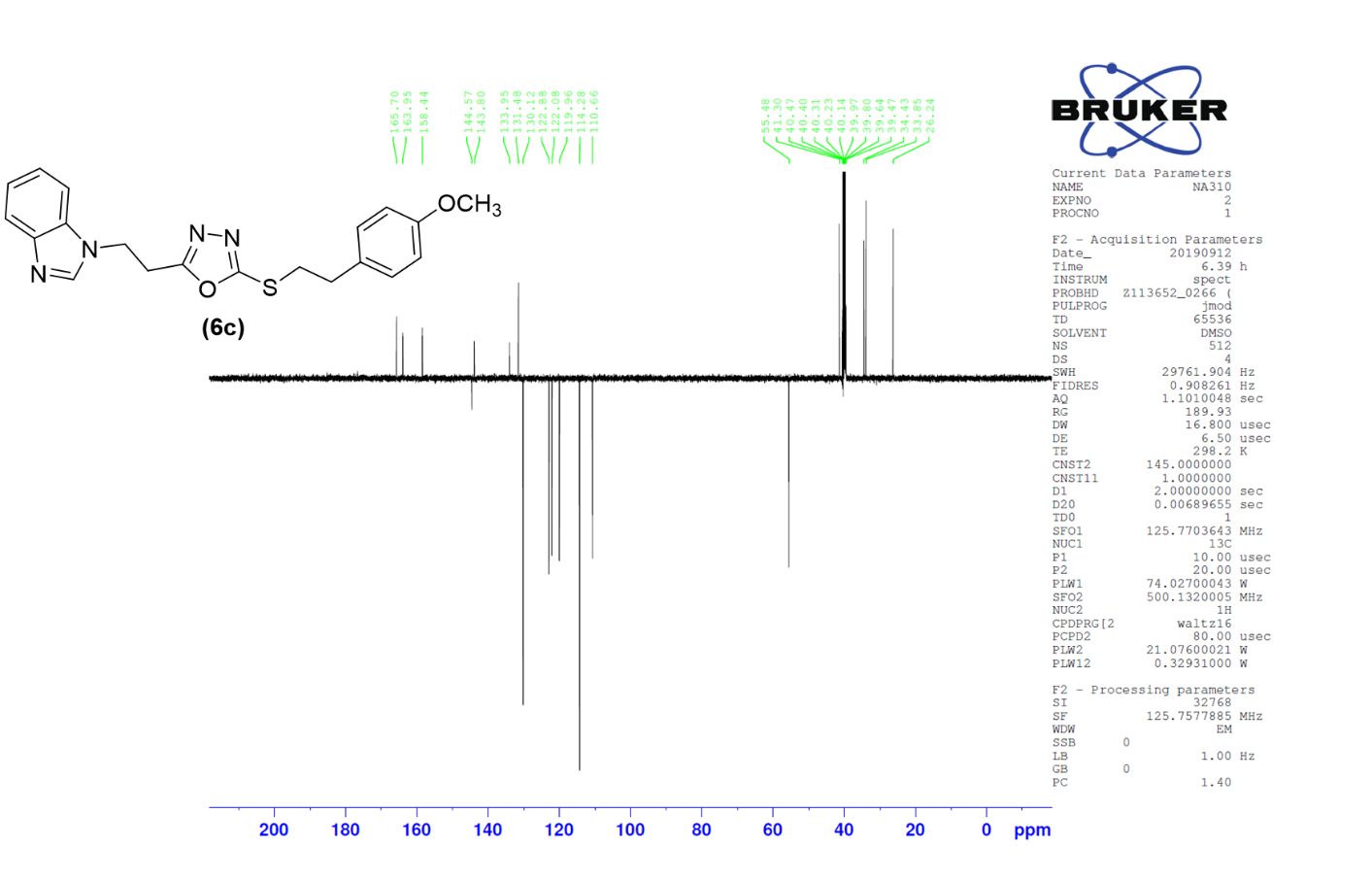


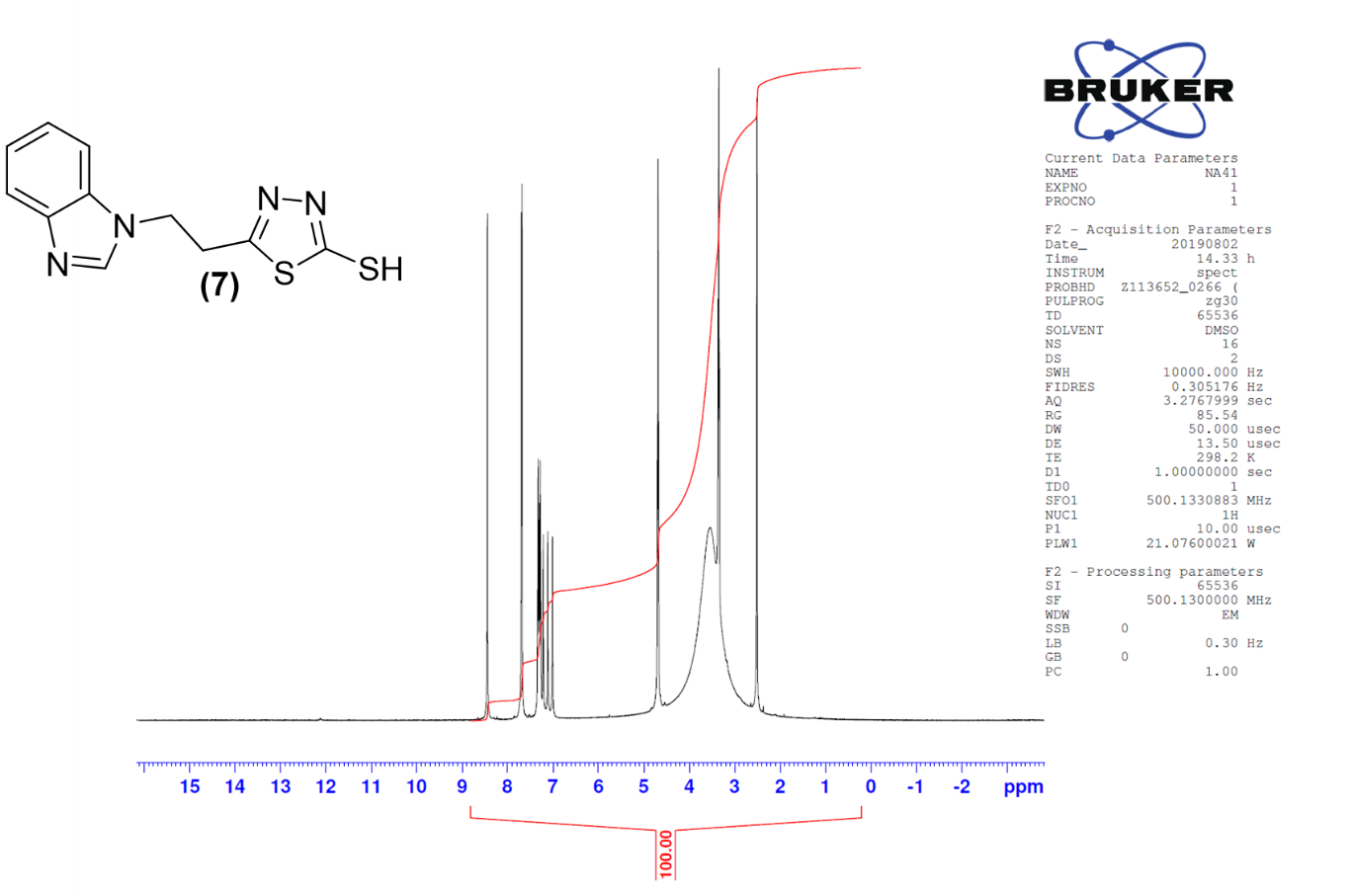


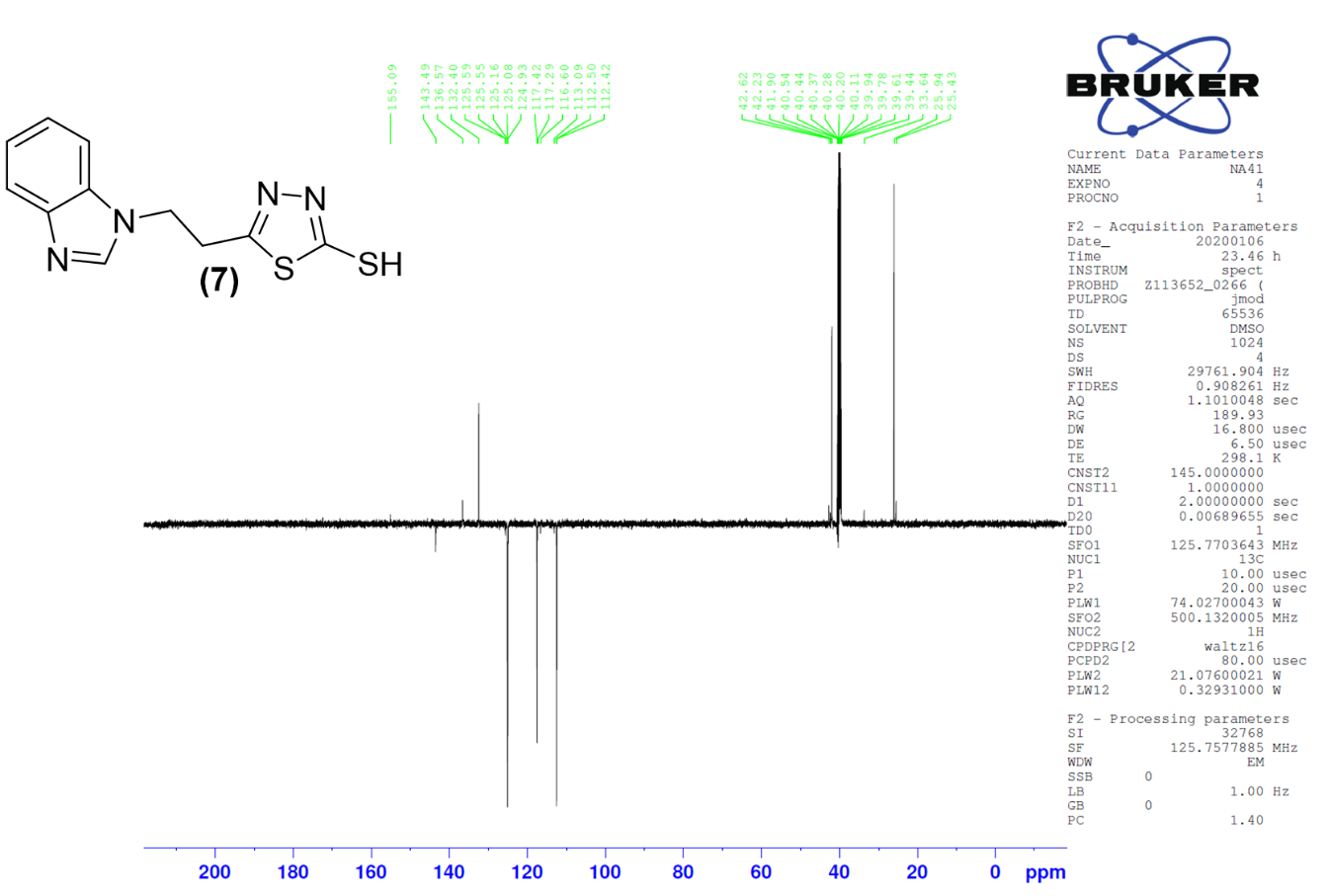


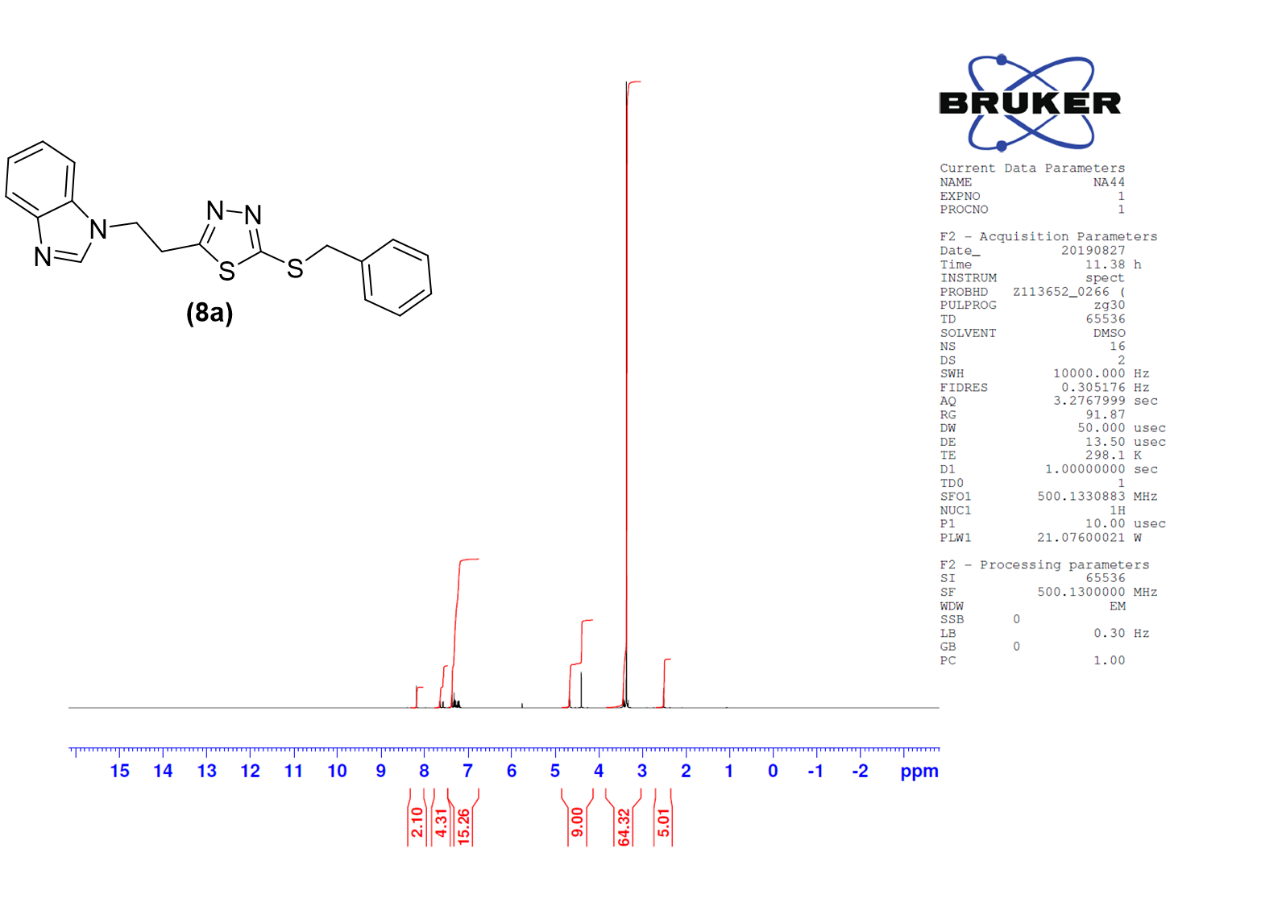


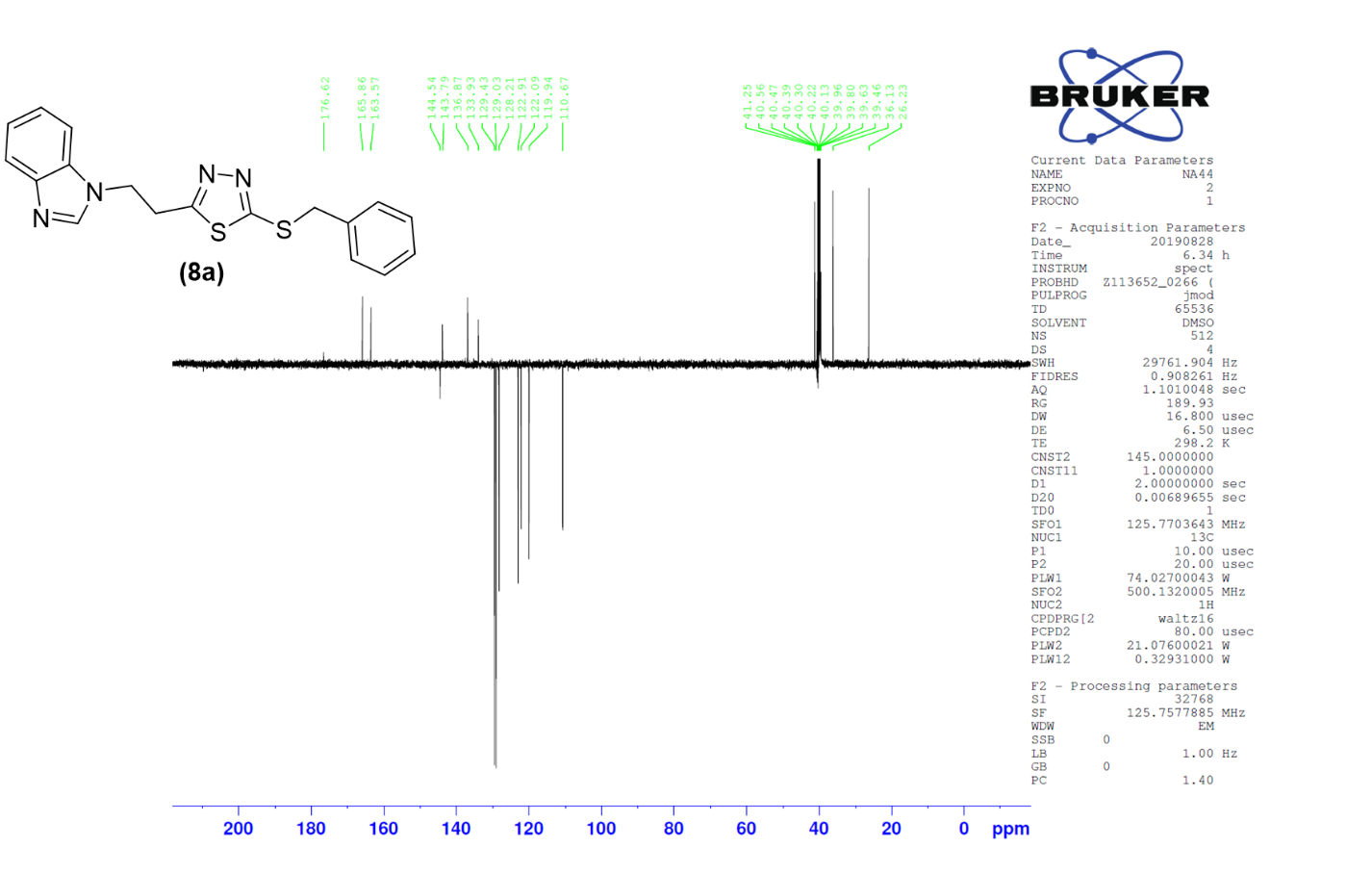


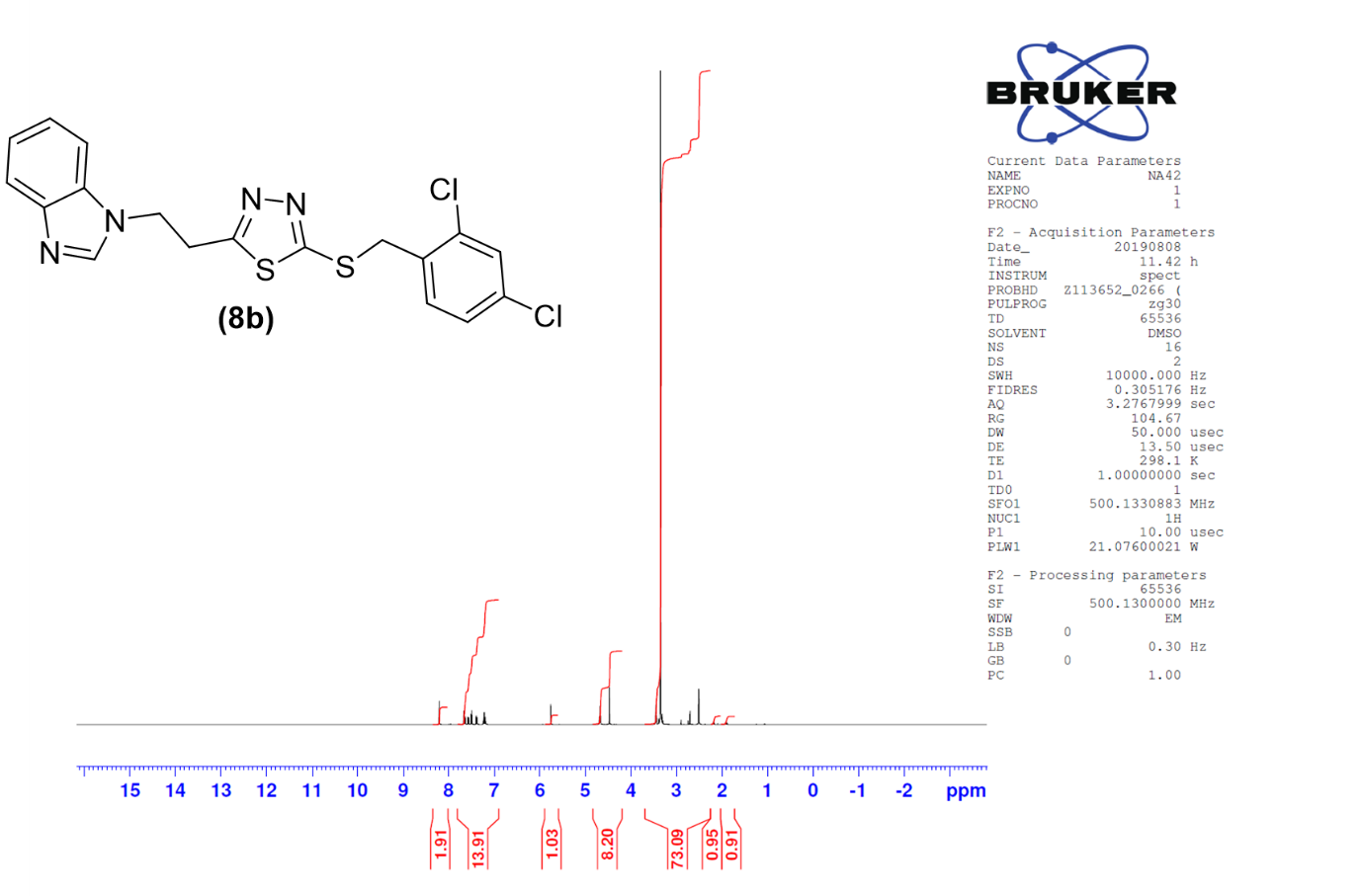


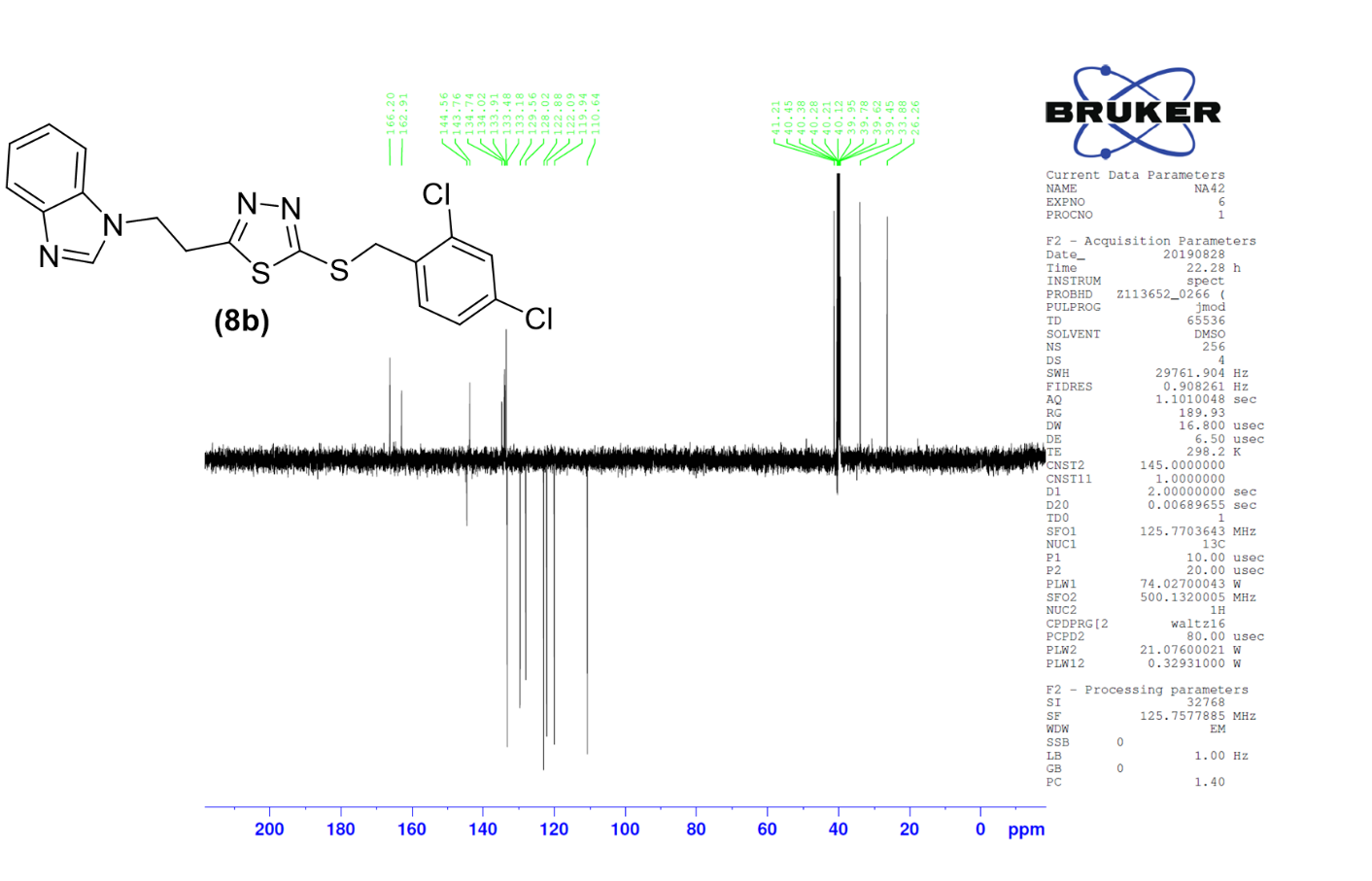


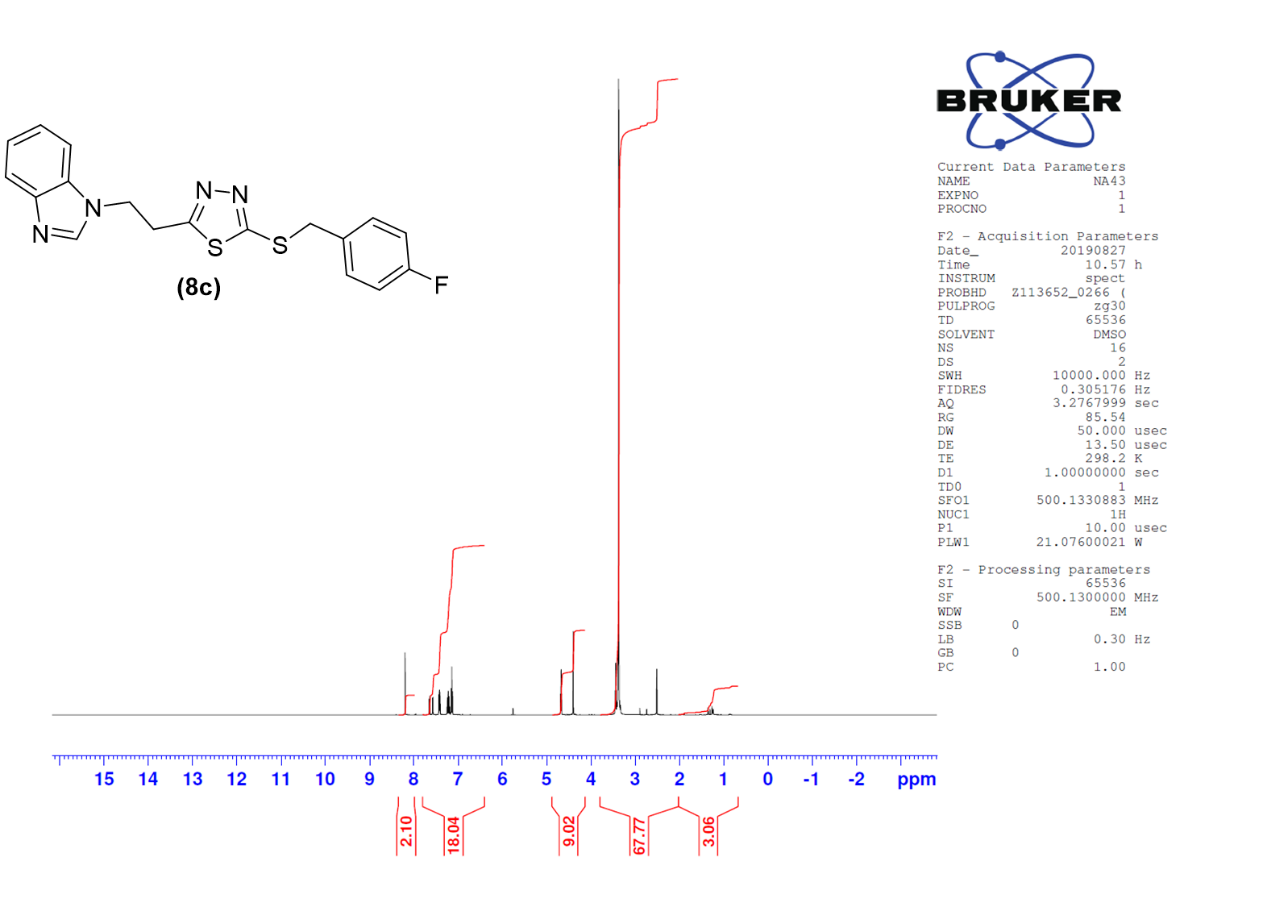


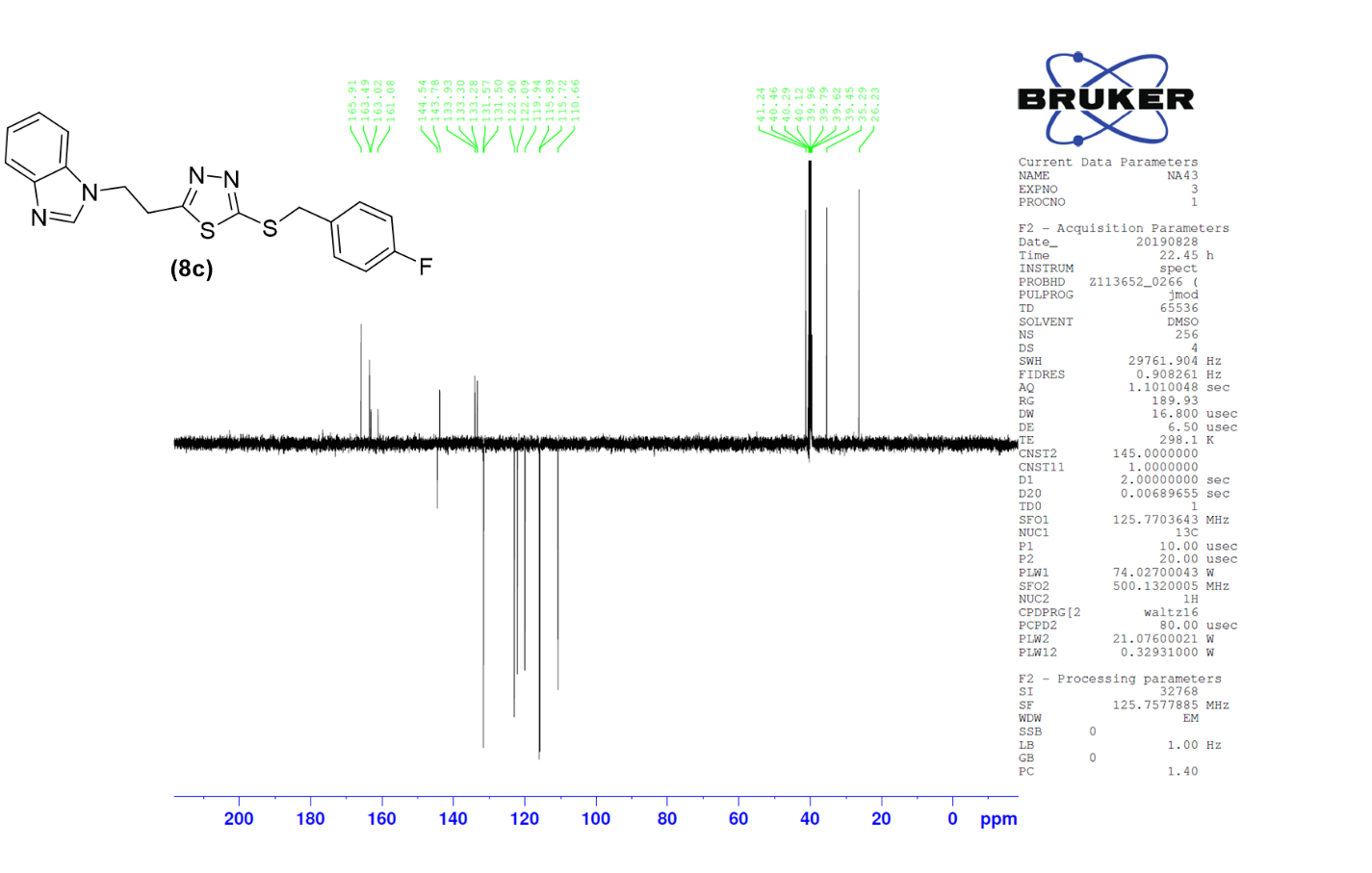


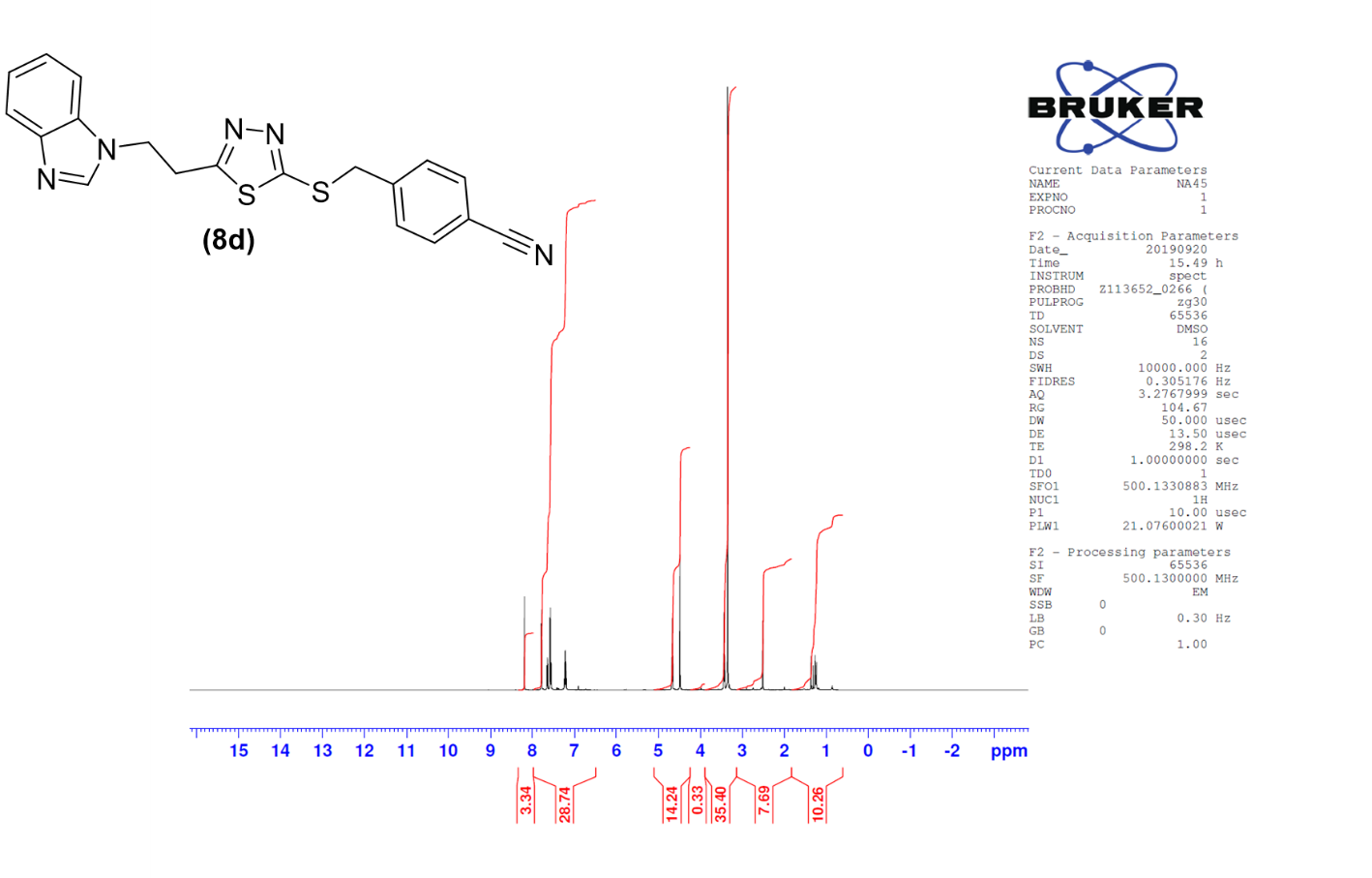


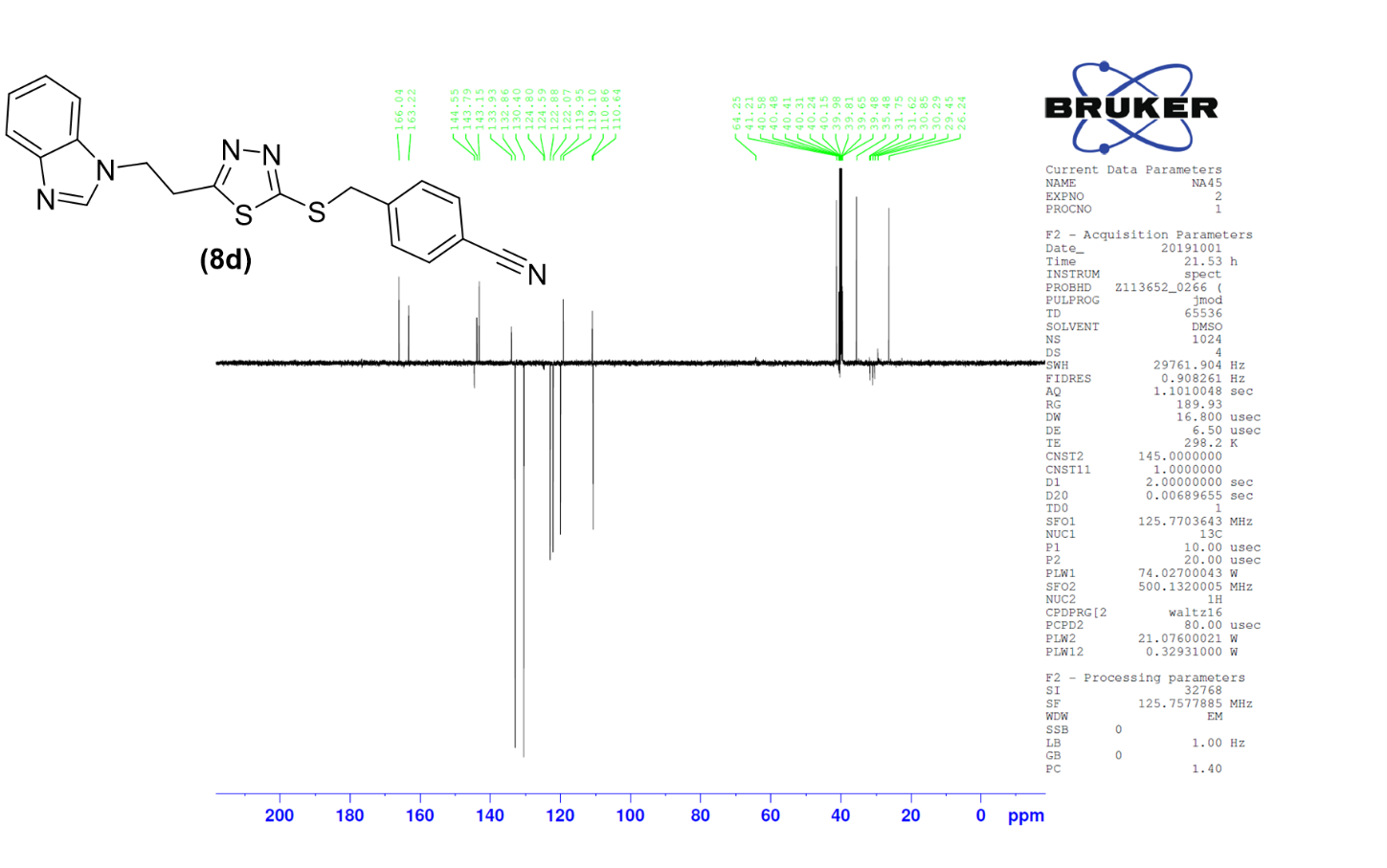


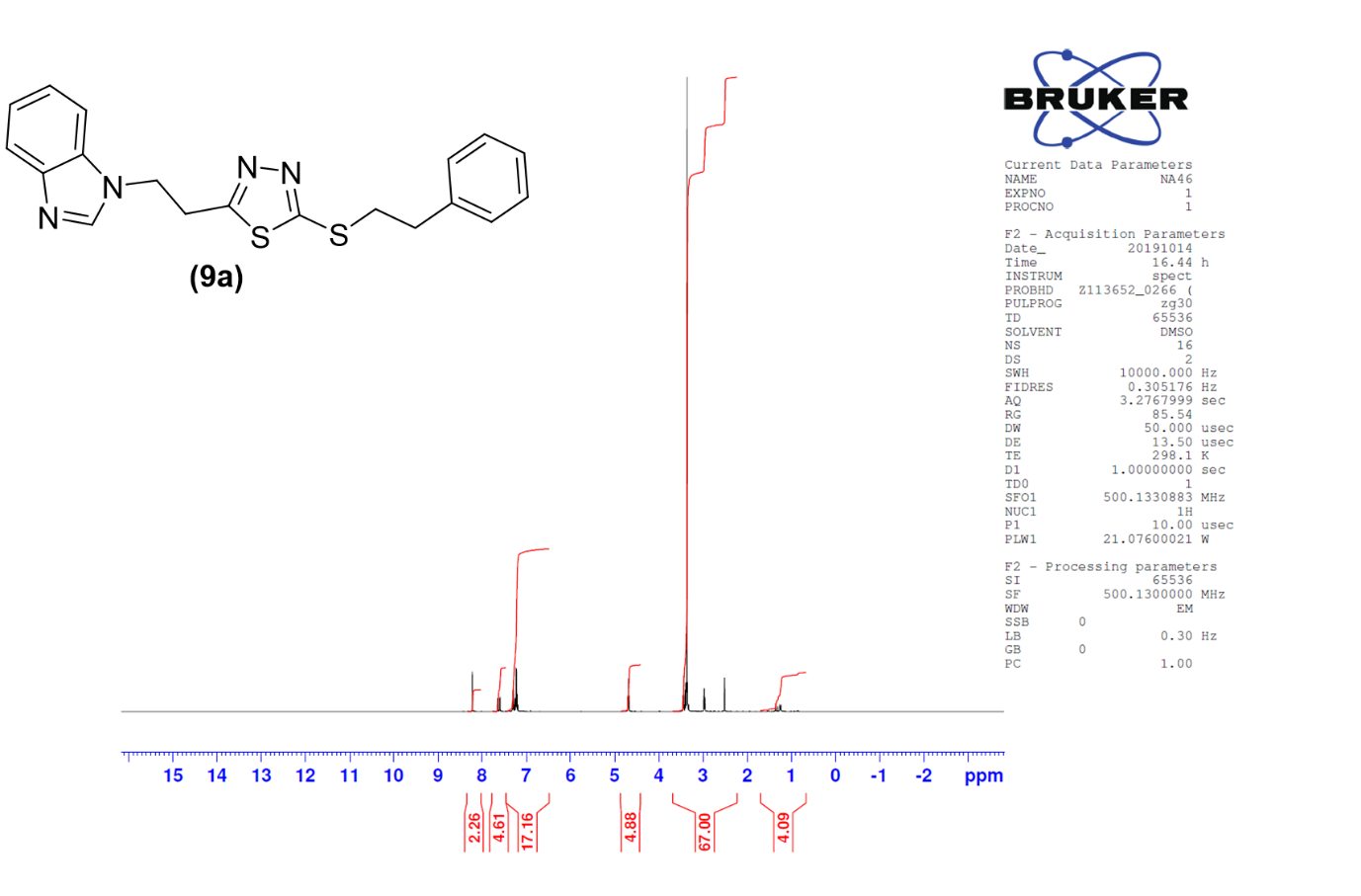


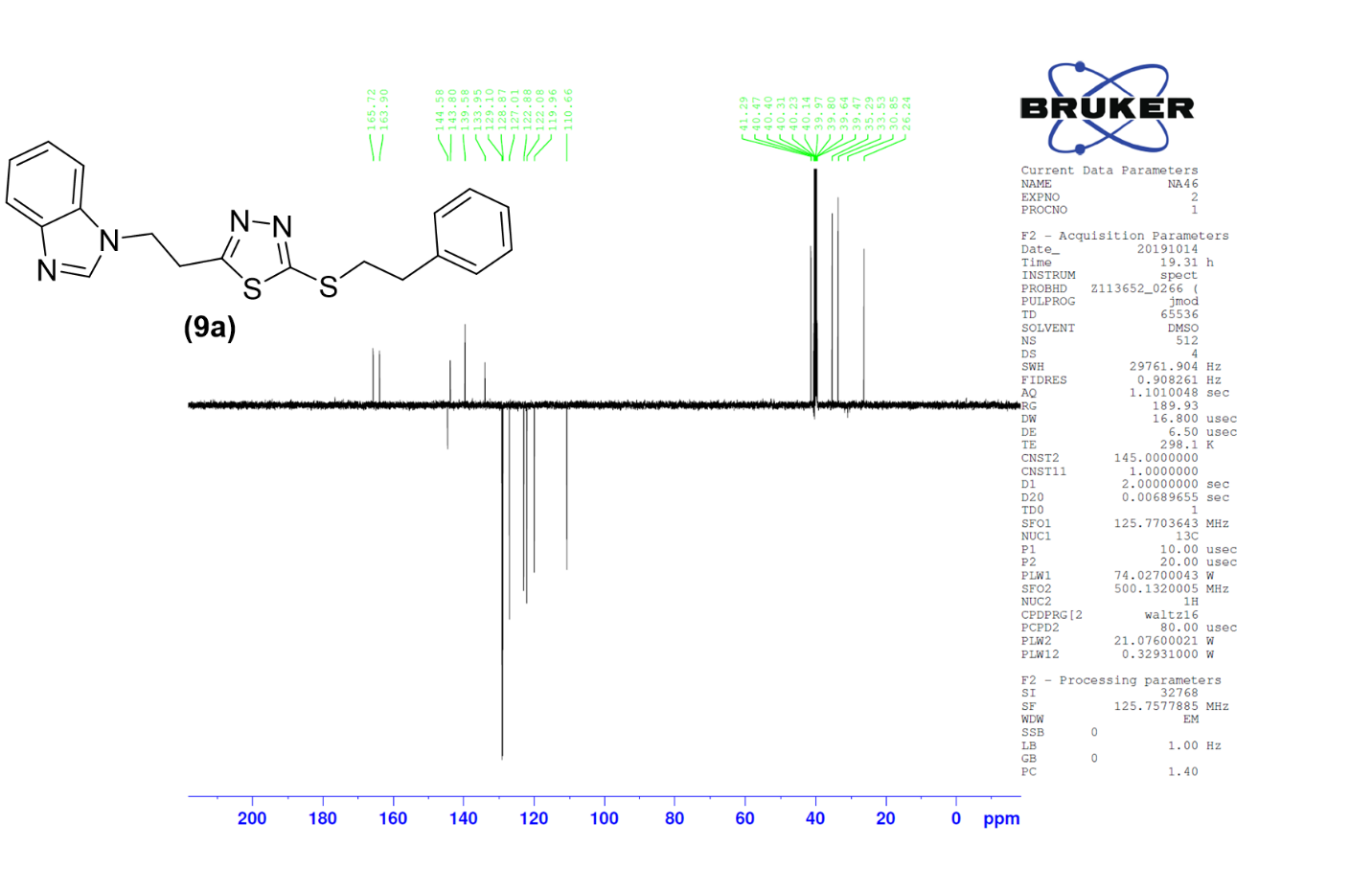


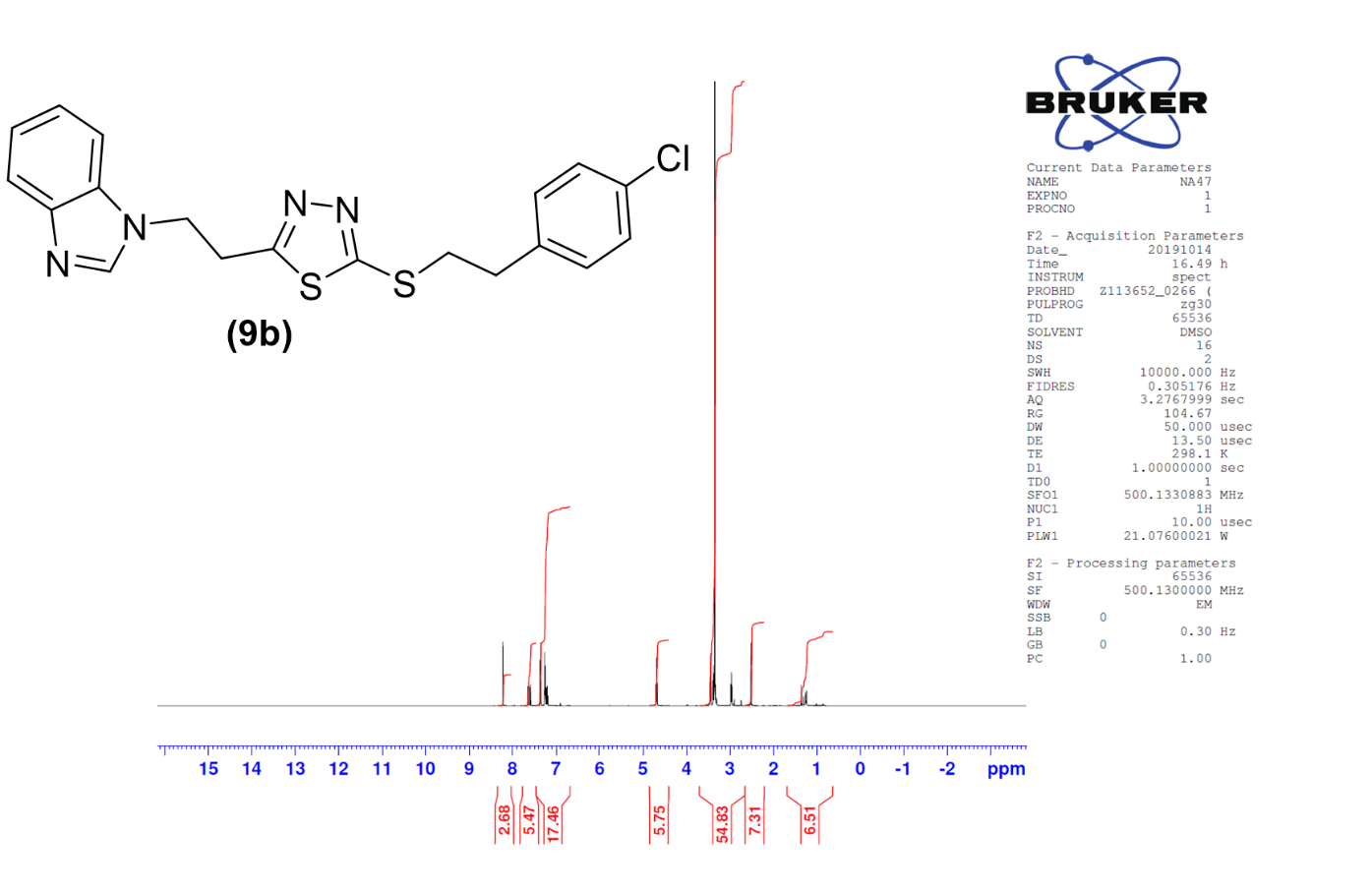


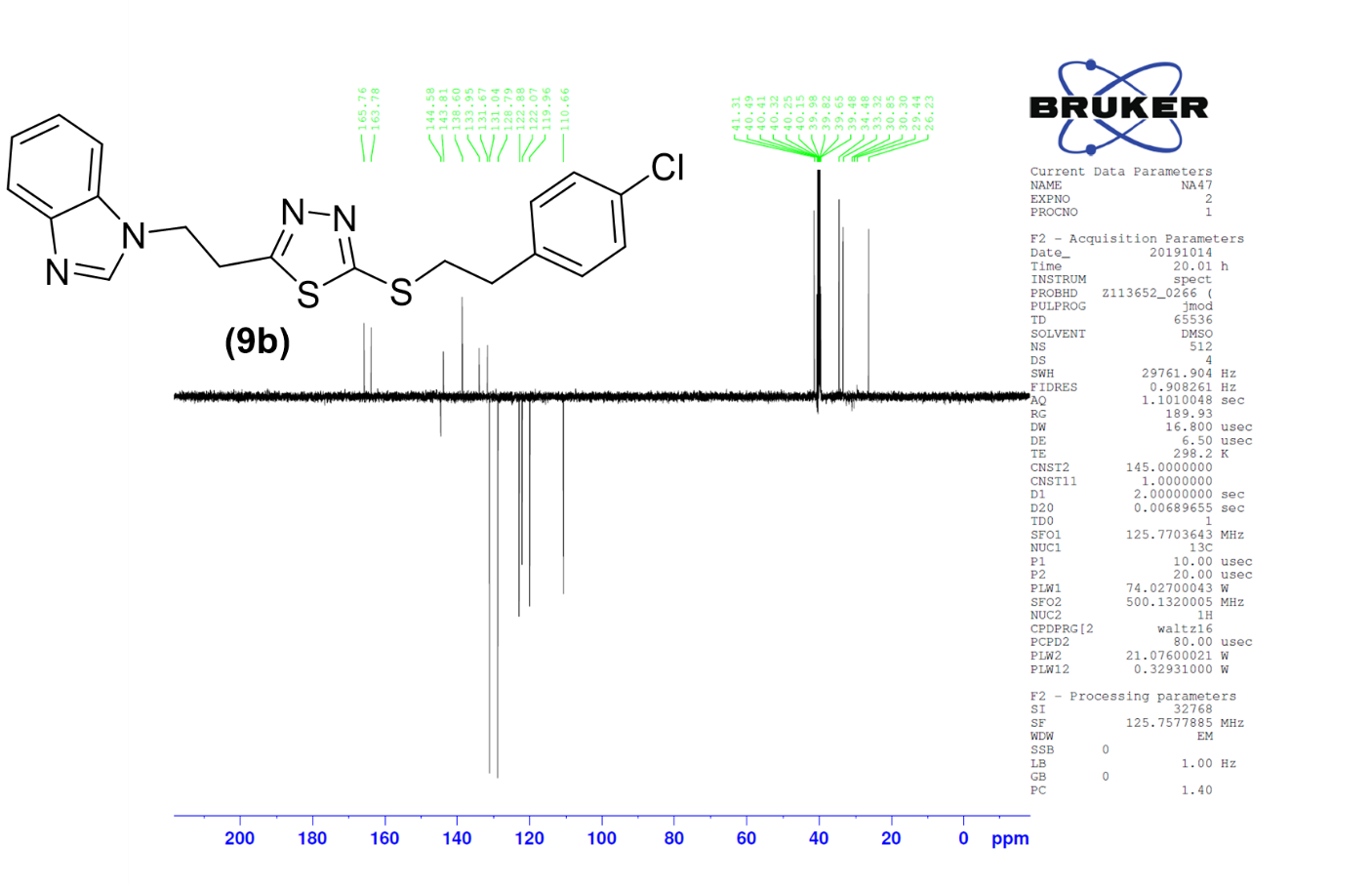


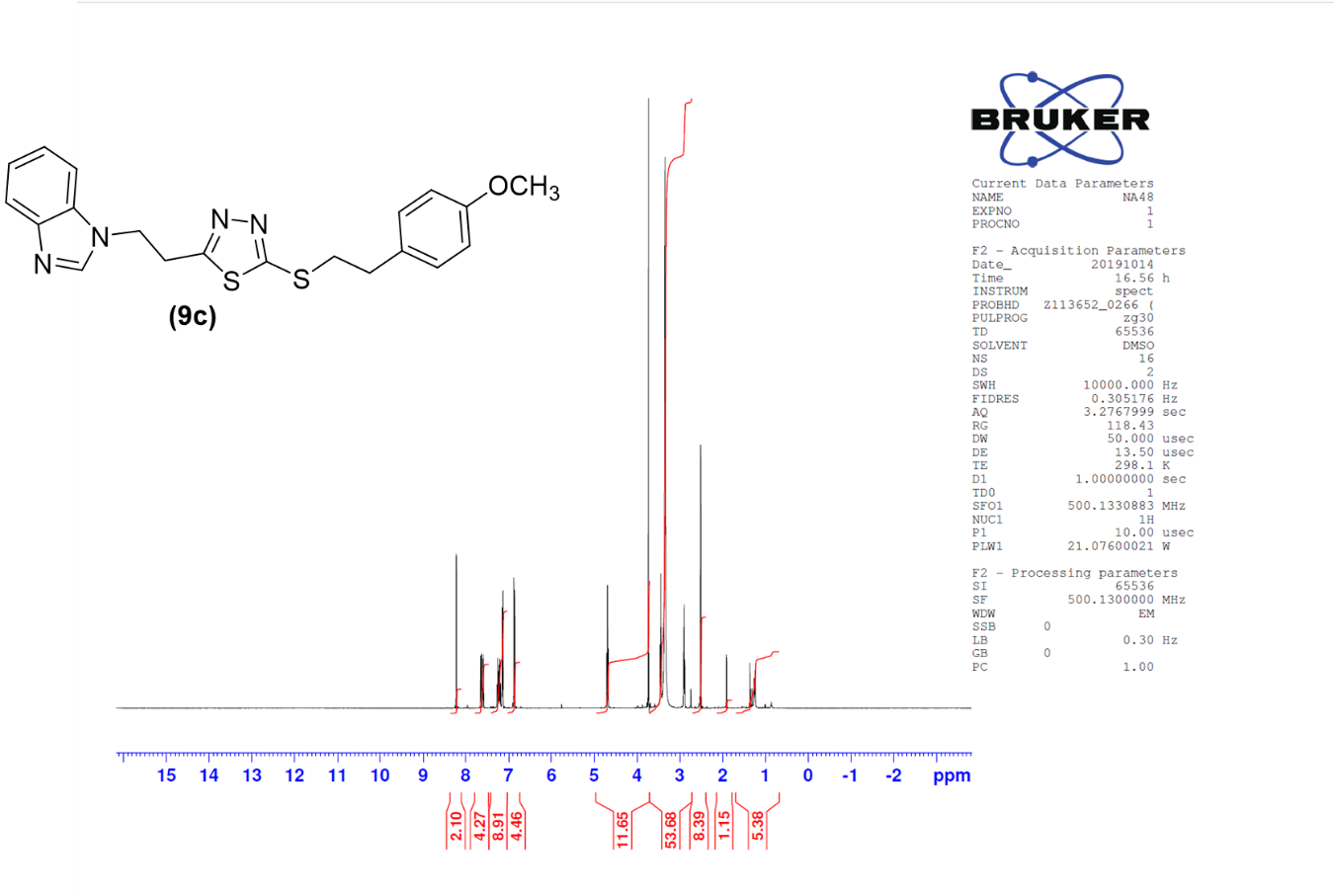


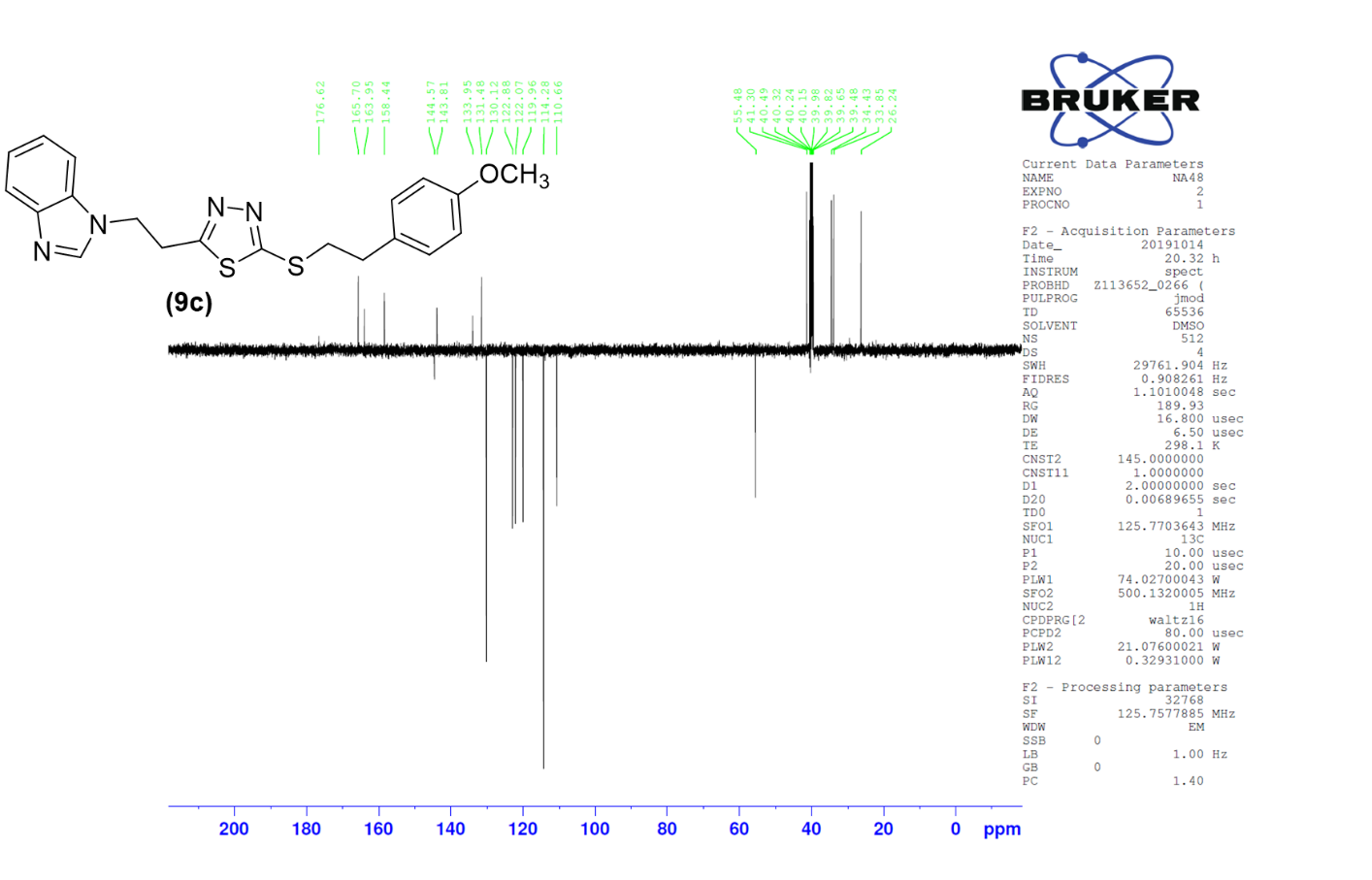


**c) HPLC traces****:**


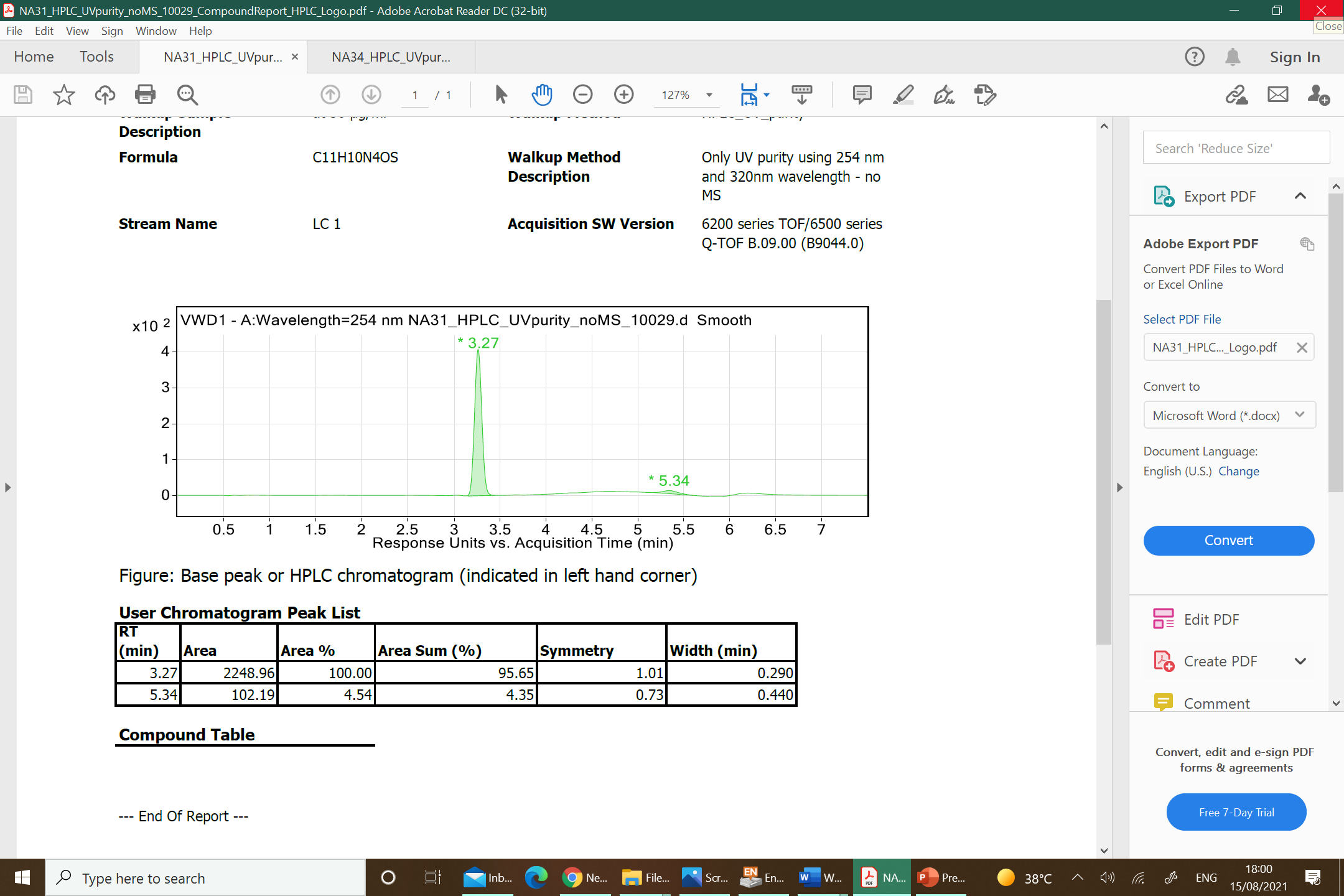


**4**


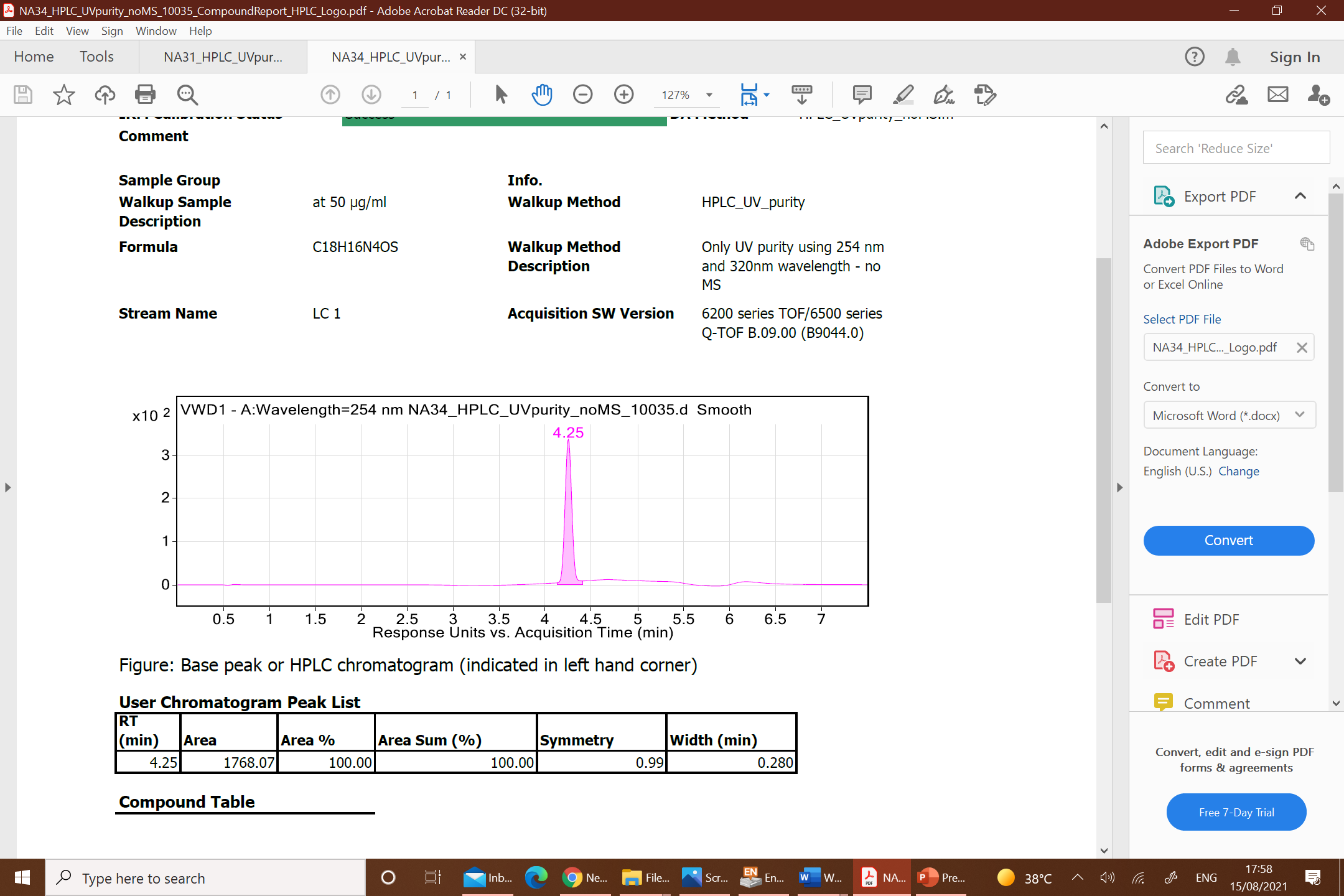


**5a**


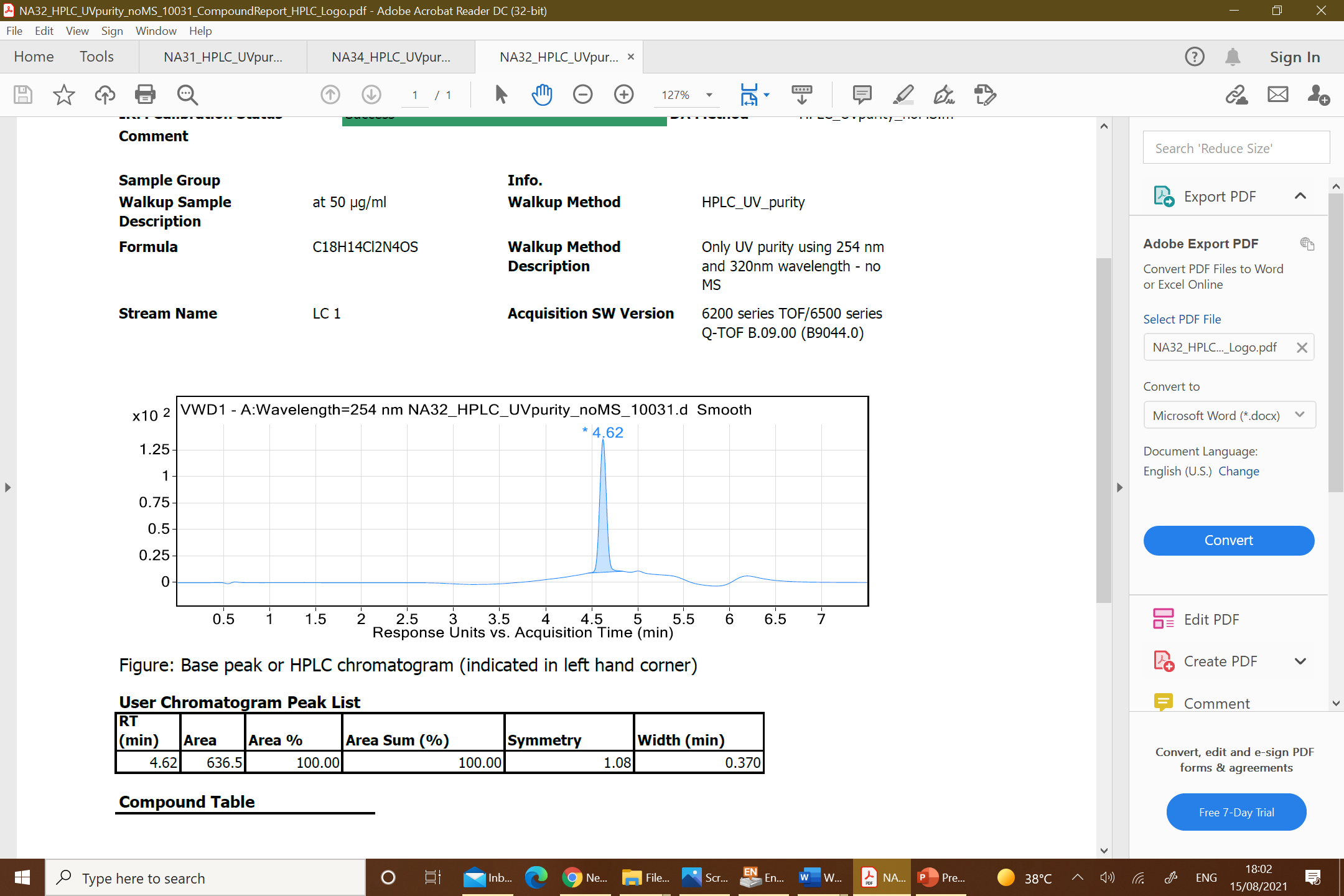


**5b**


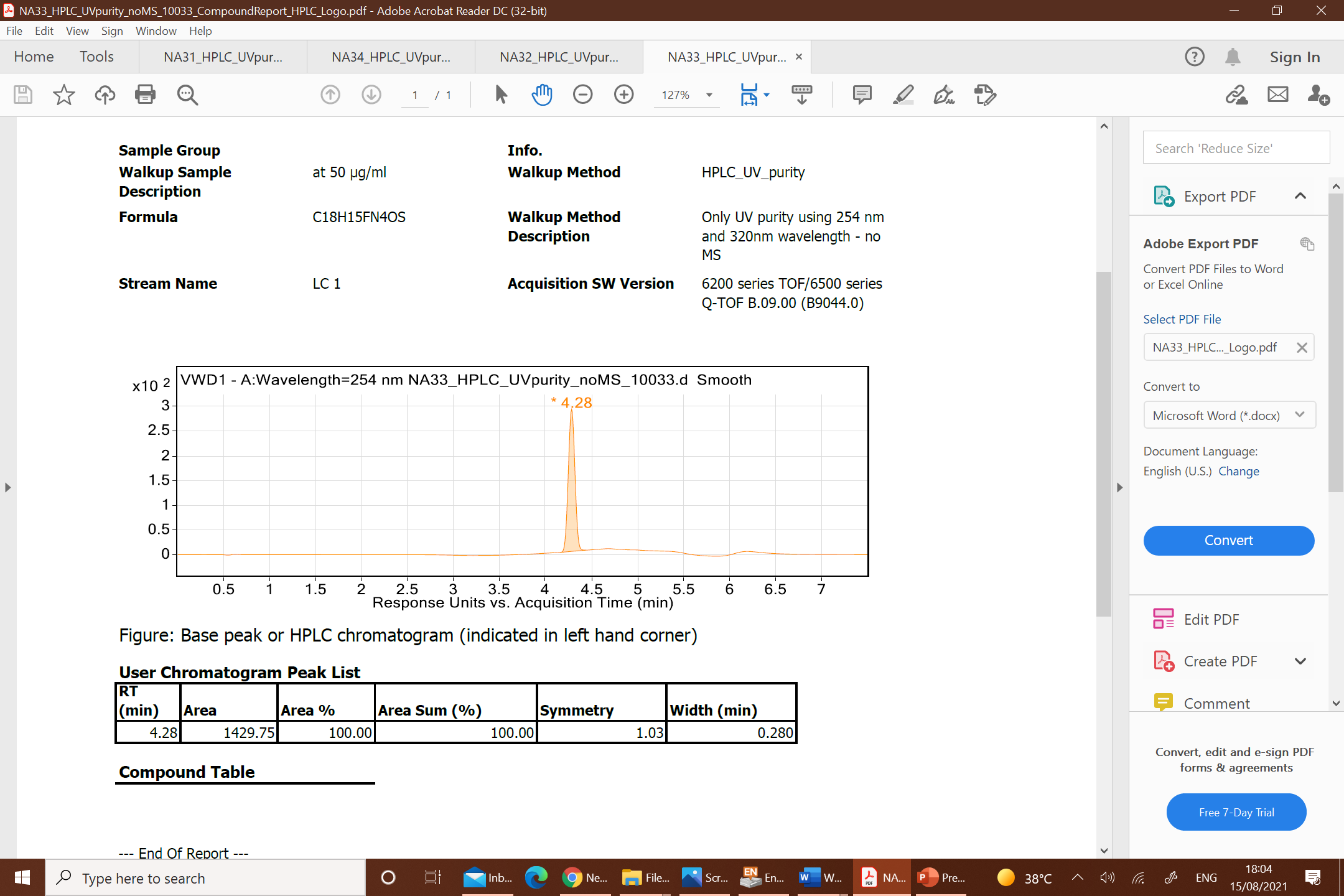


**5c**


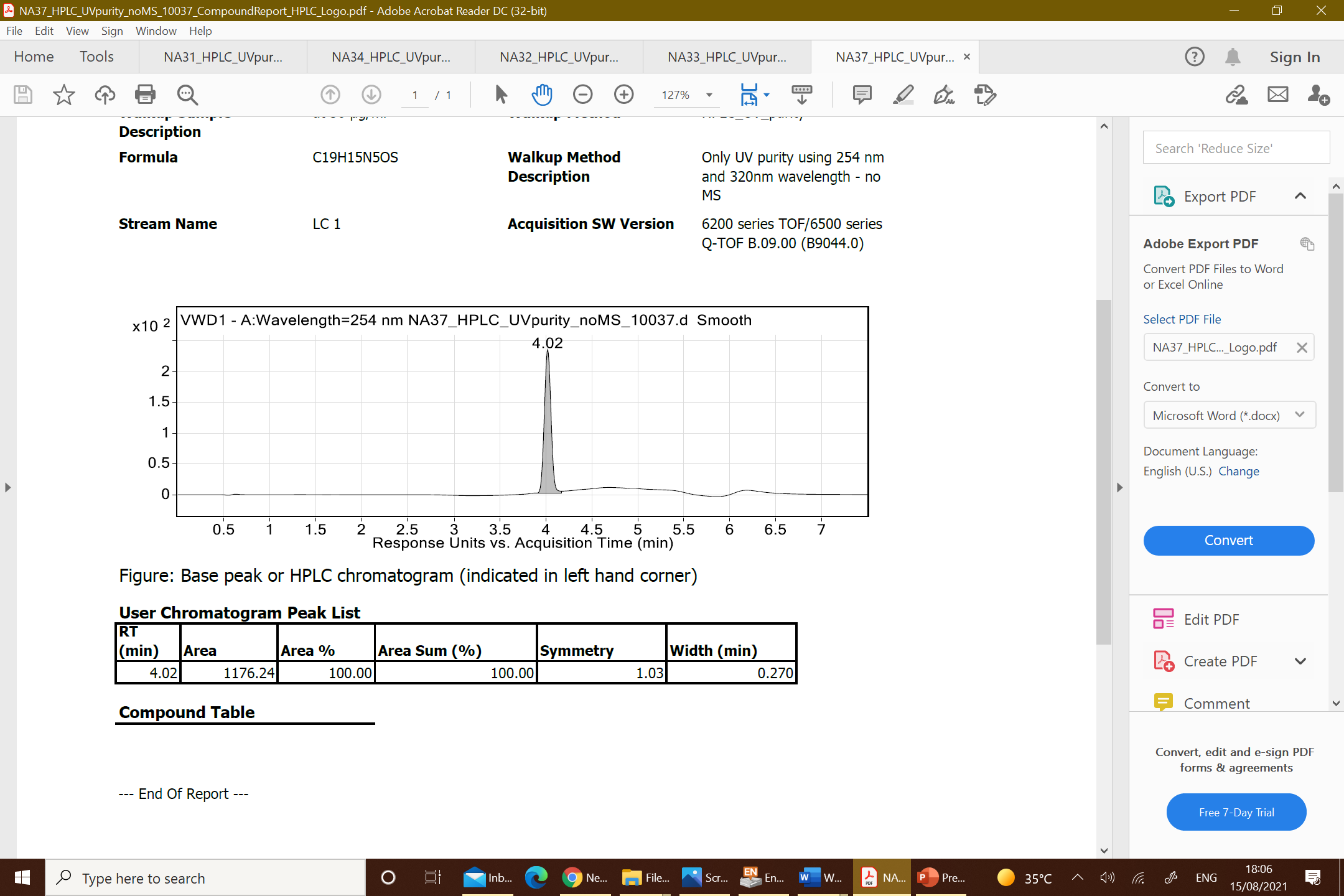


**5d**


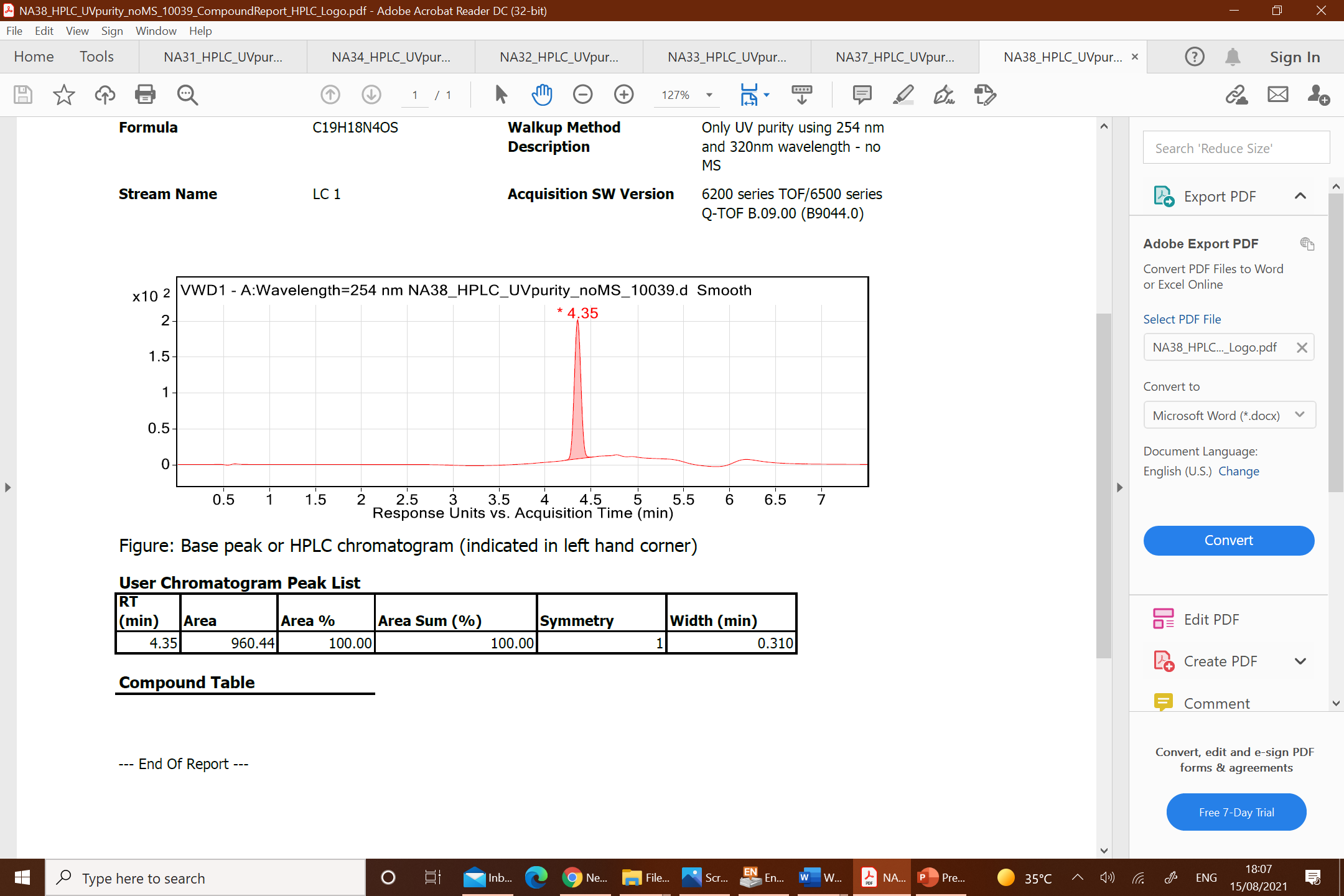


**6a**


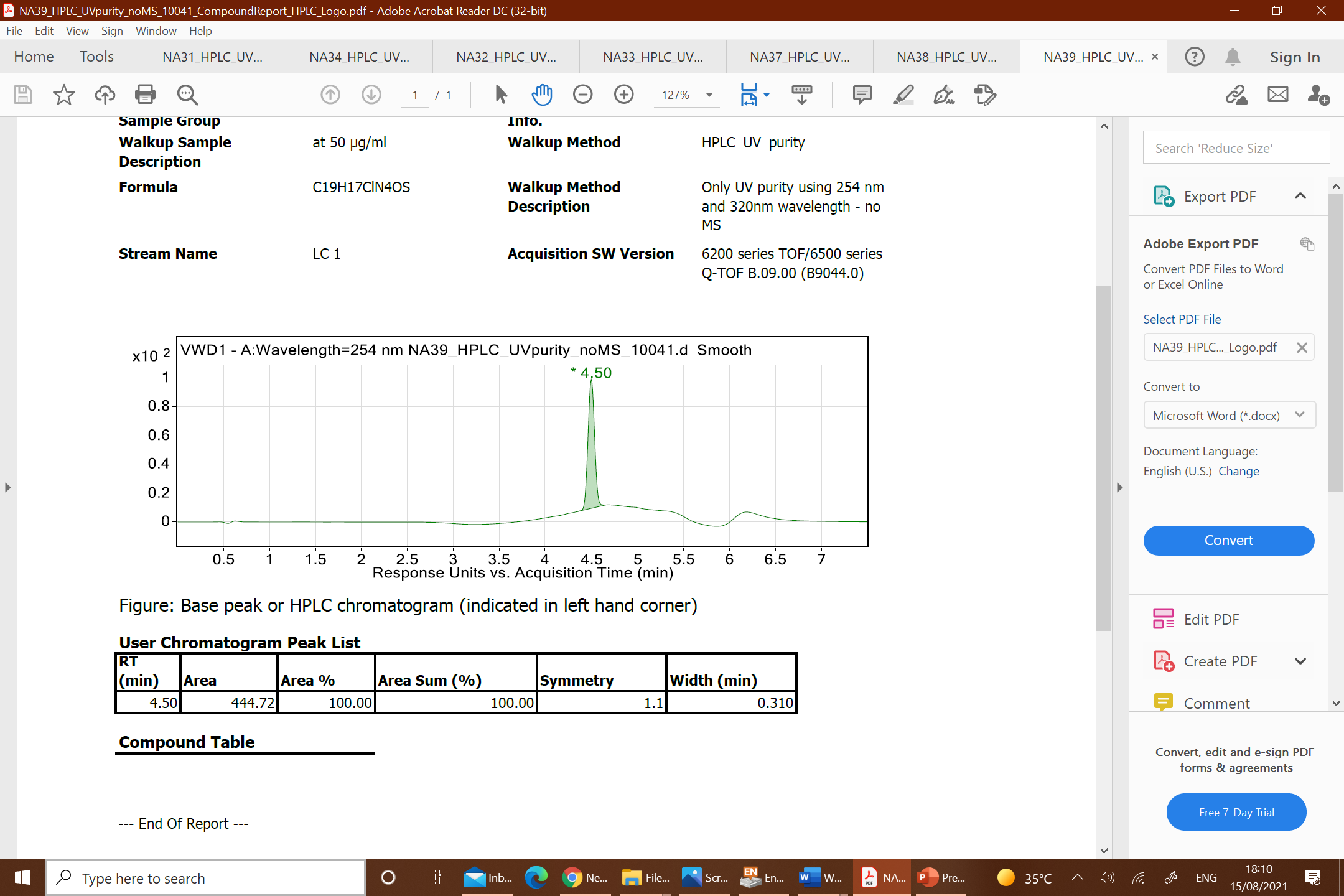


**6b**


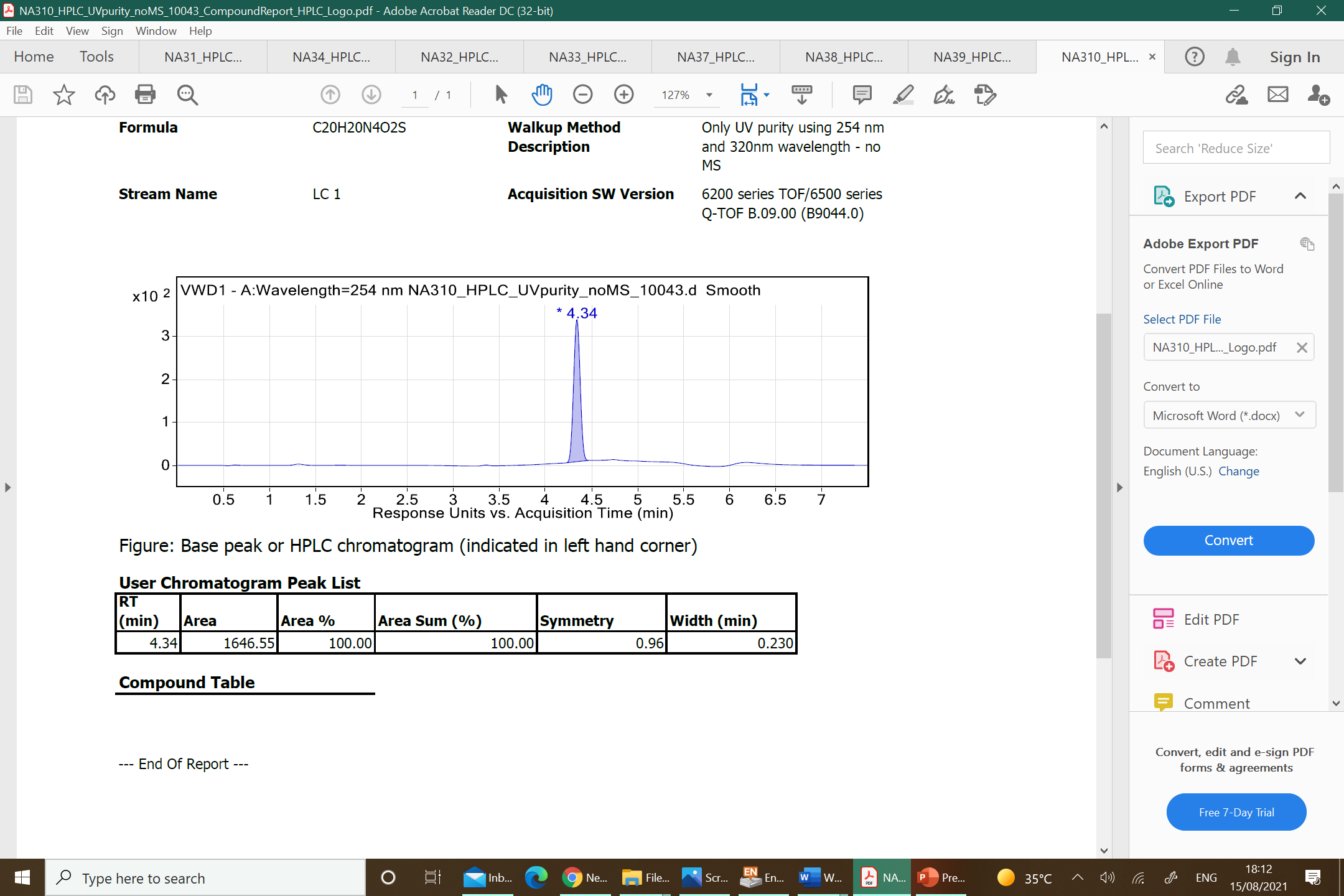


**6c**


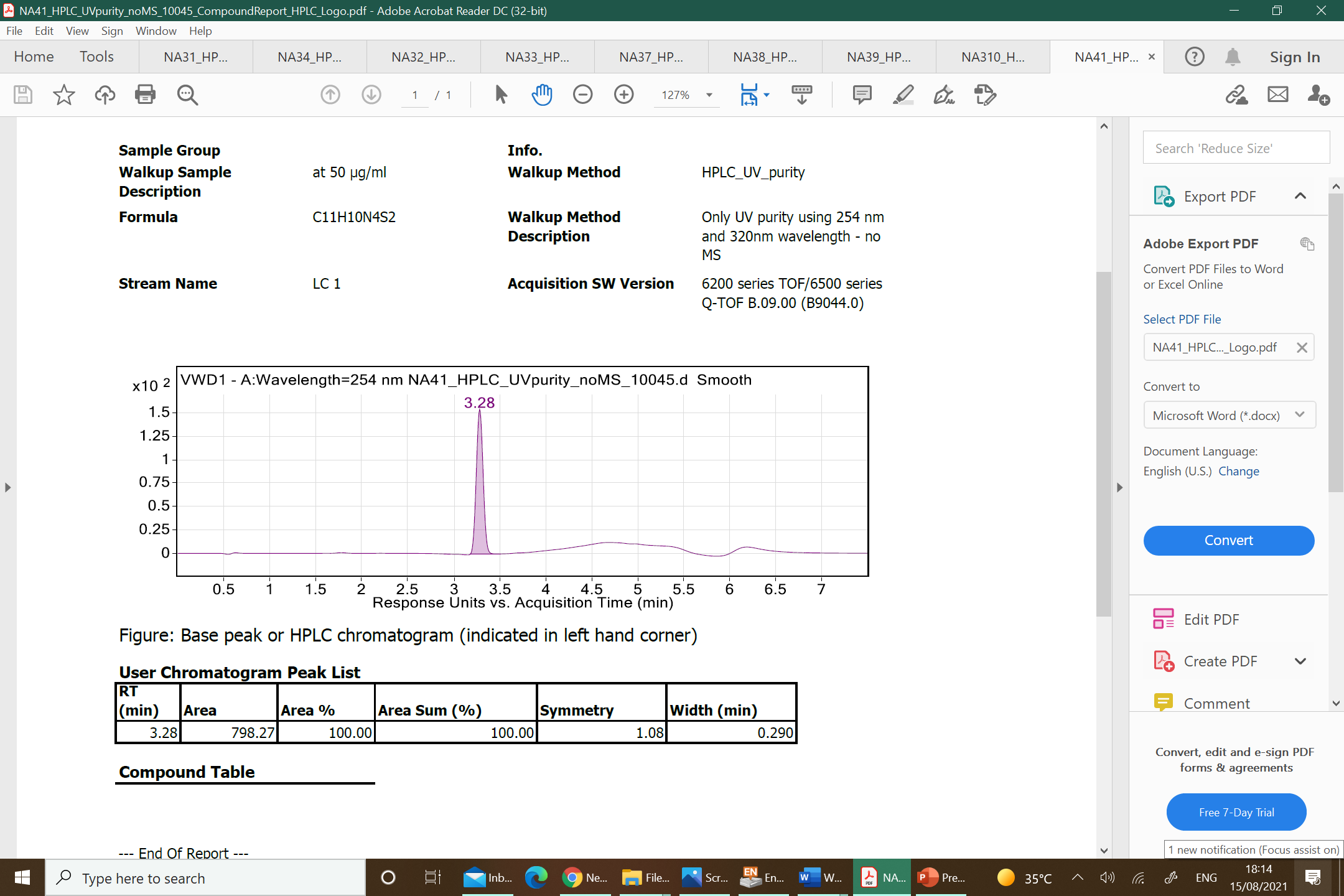


**7**


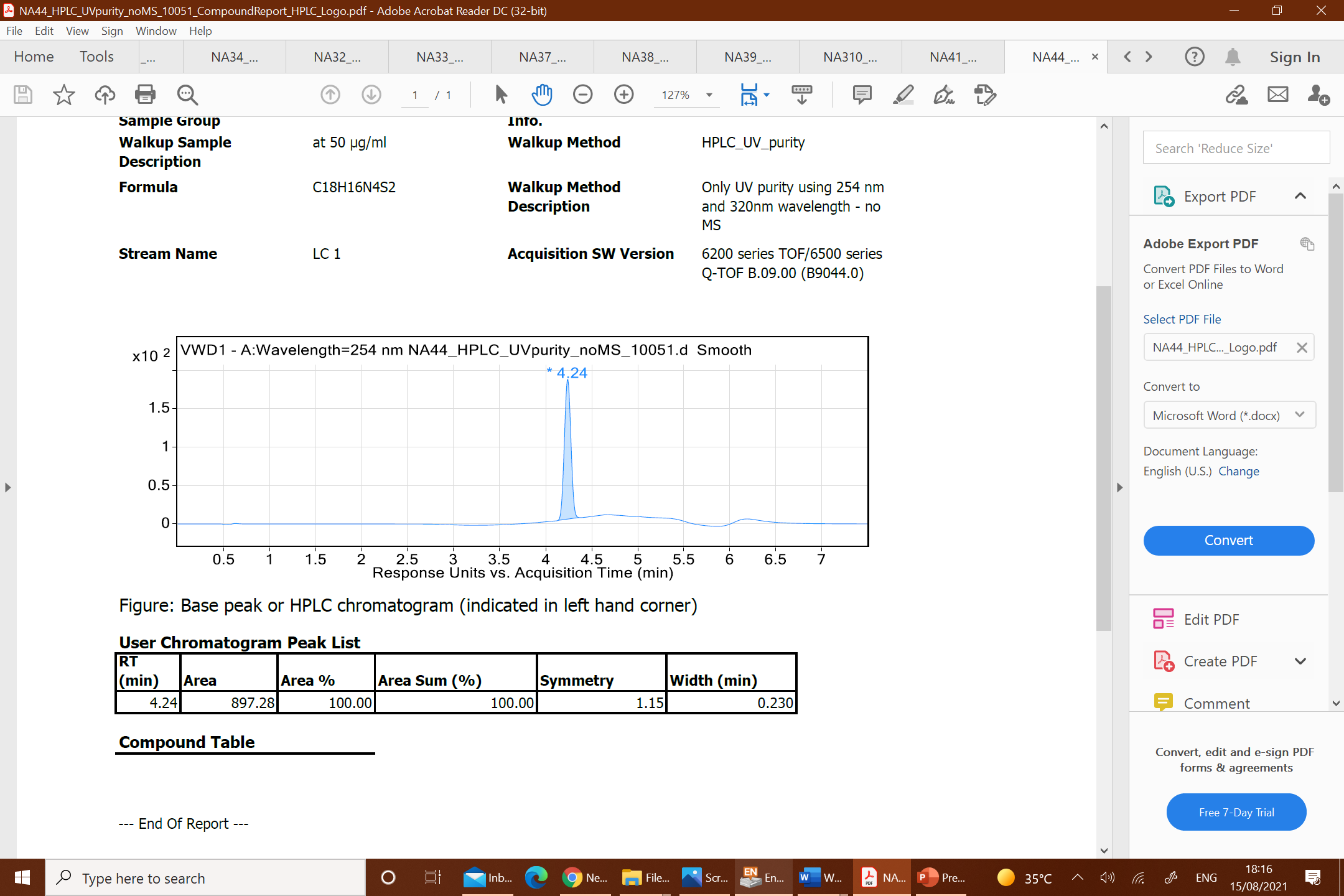


**8a**


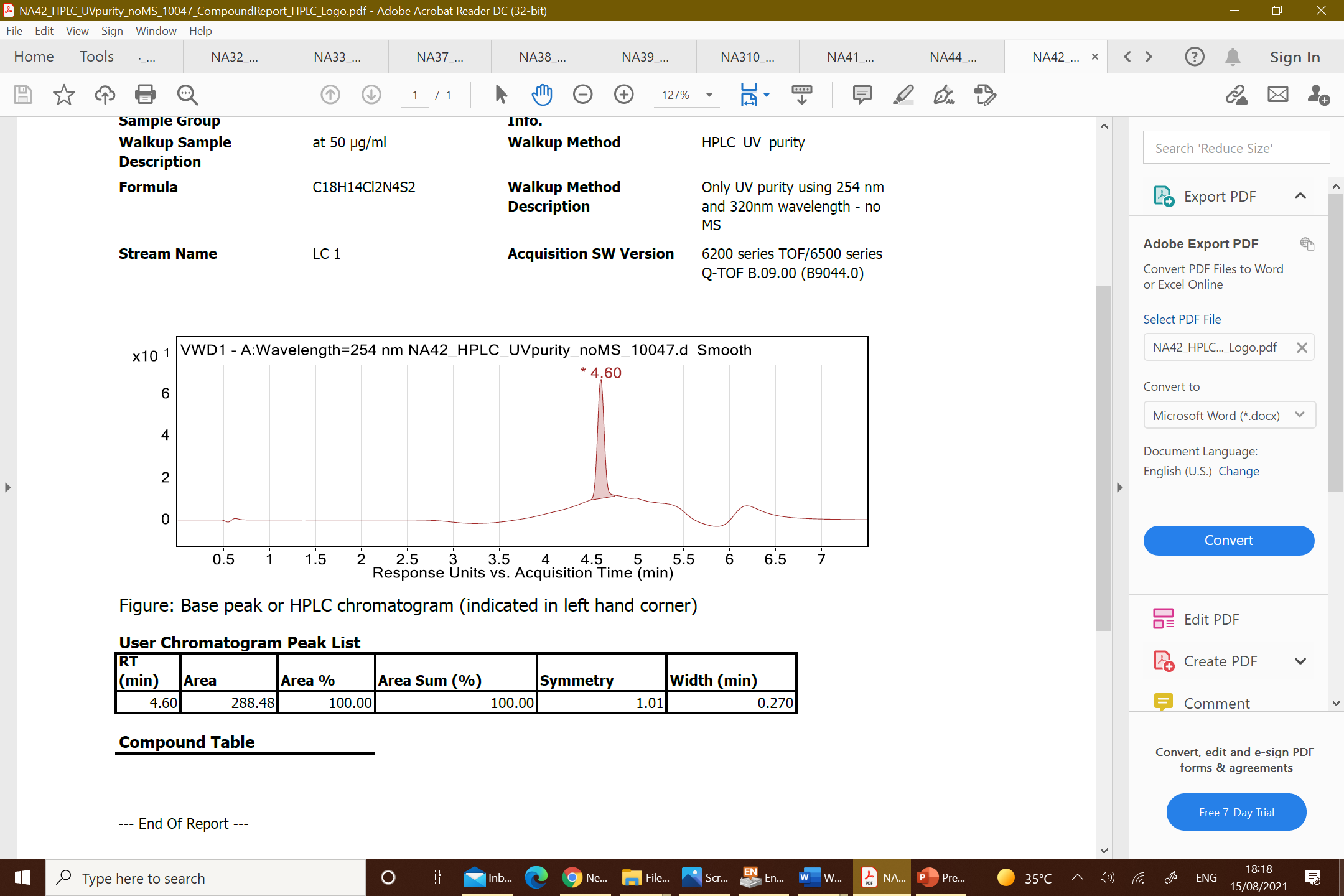


**8b**


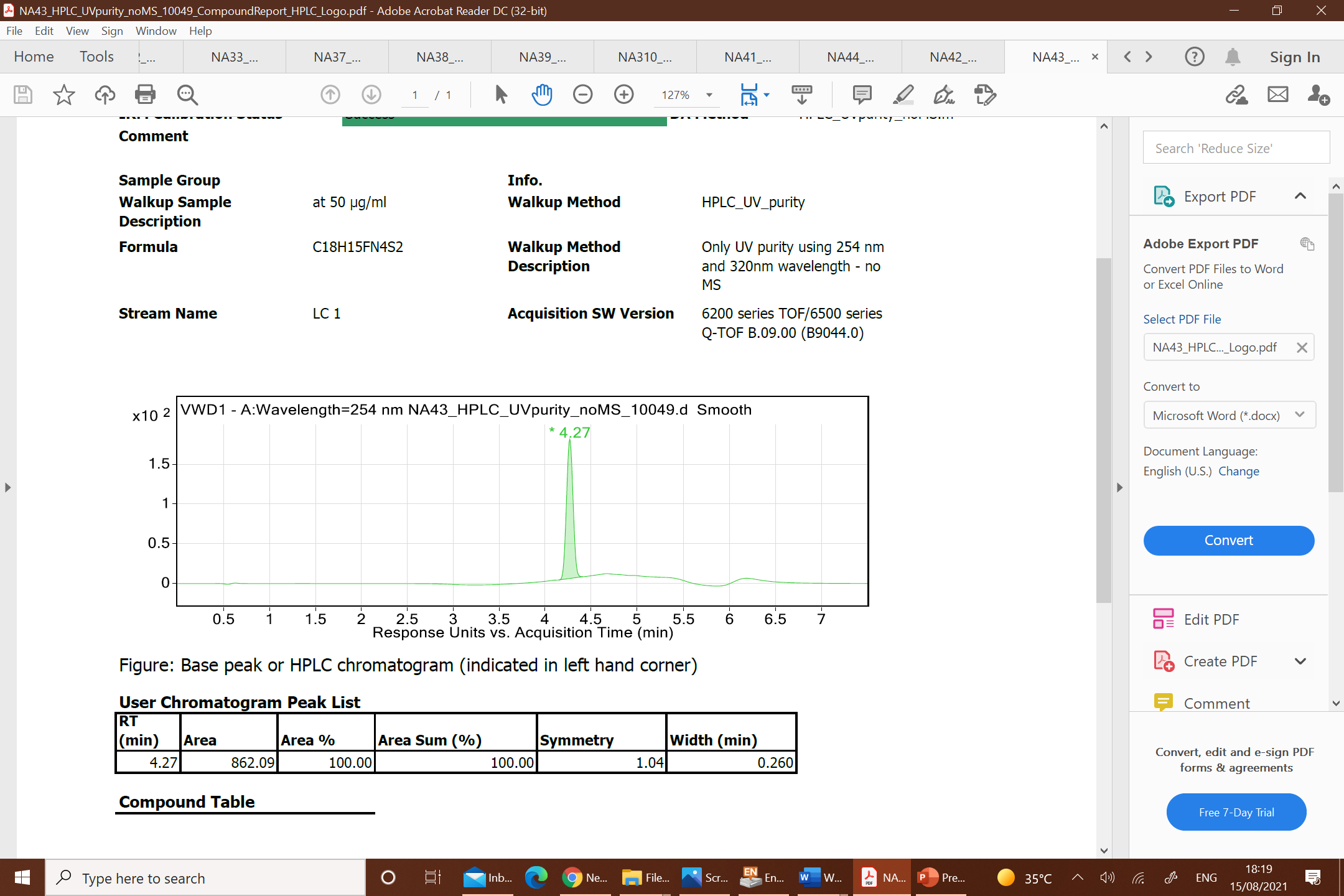


**8c**


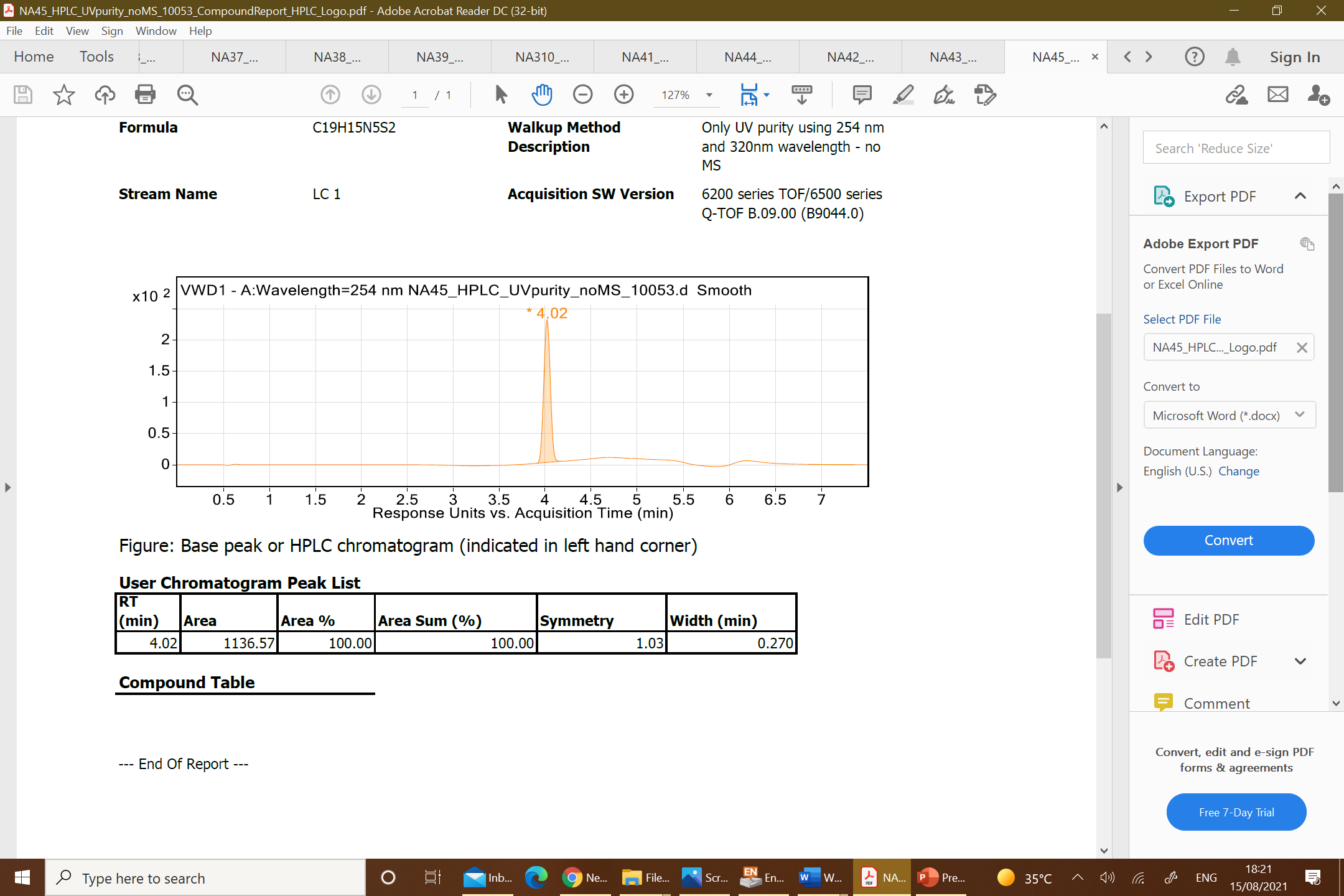


**8d**


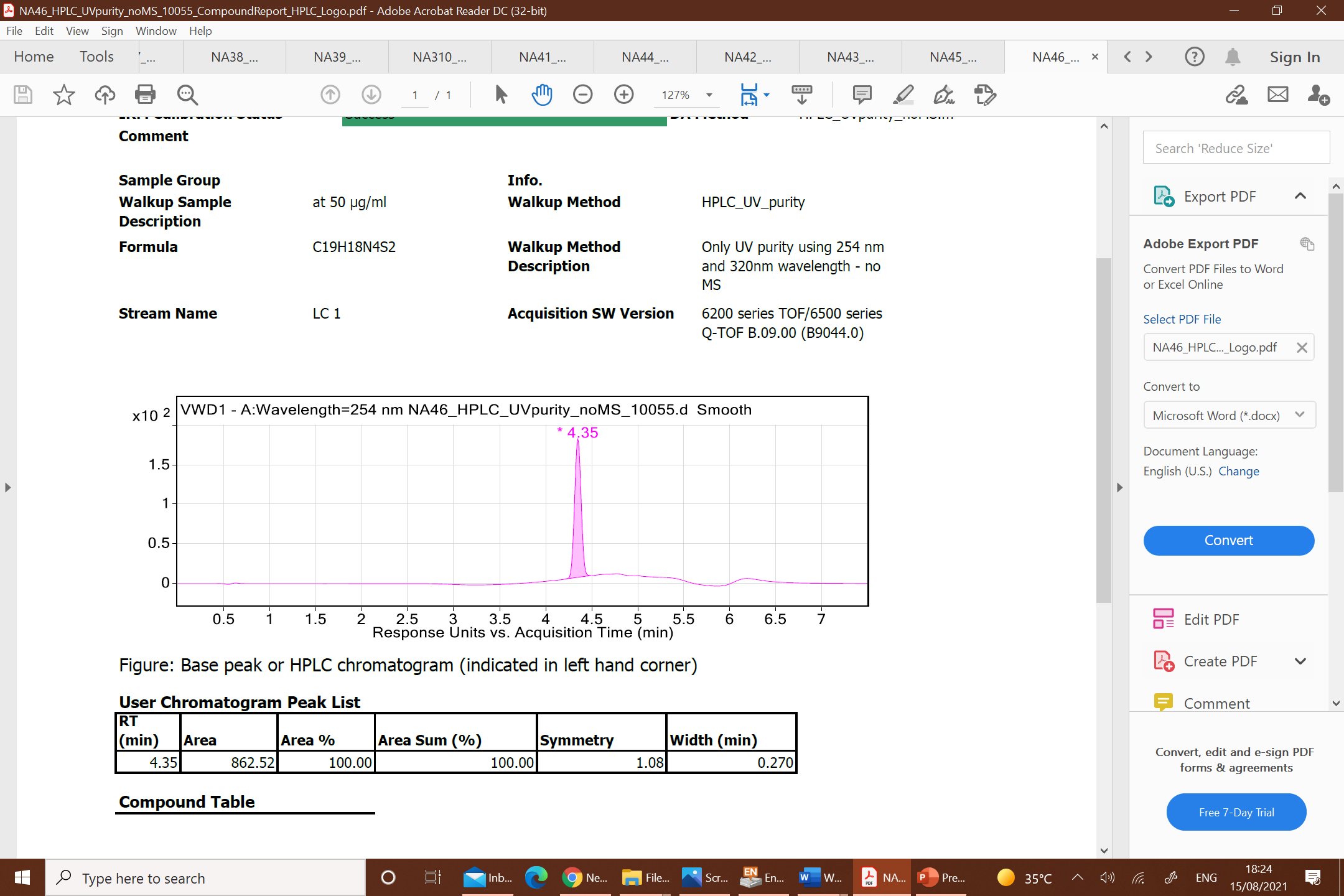


**9a**


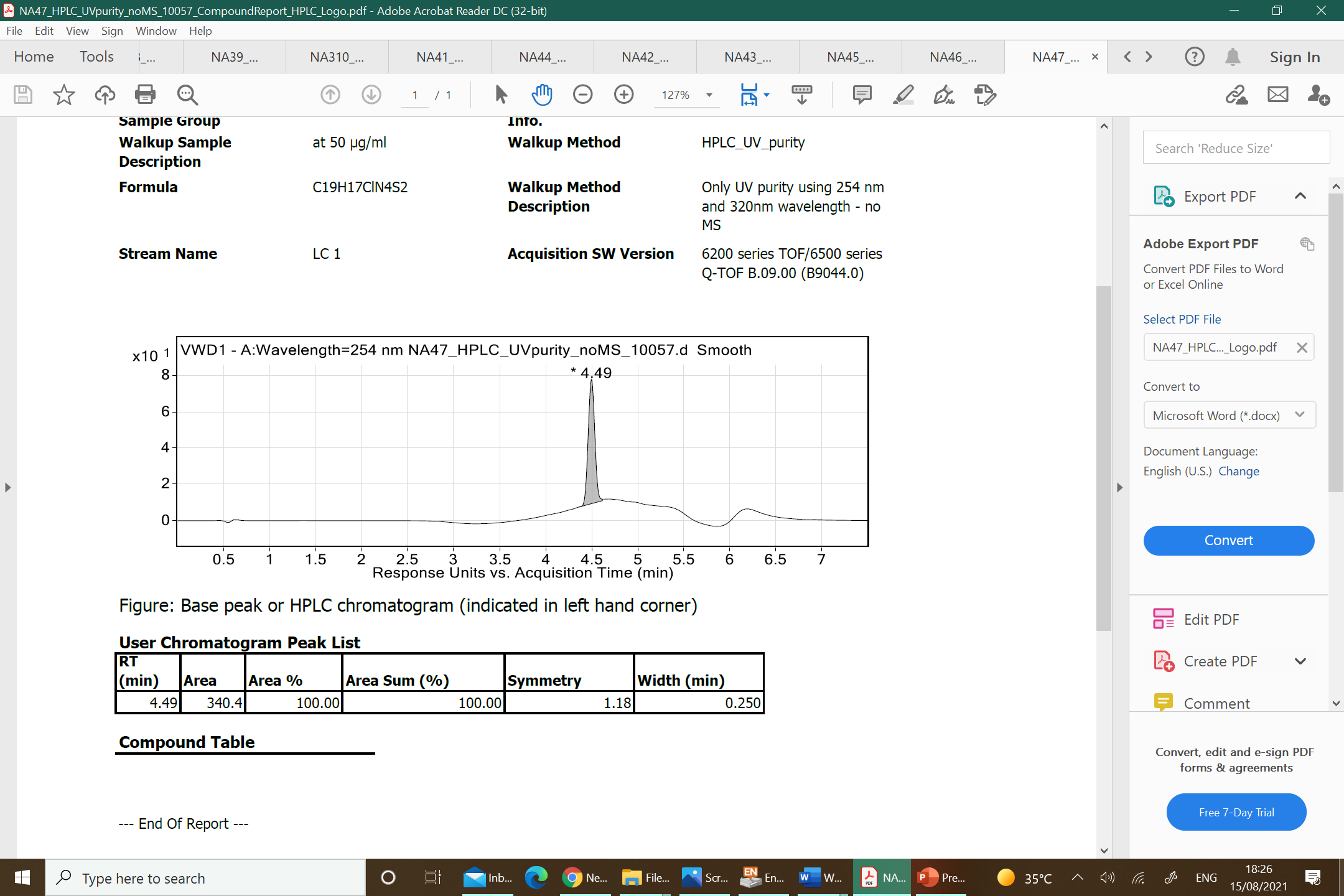


**9b**


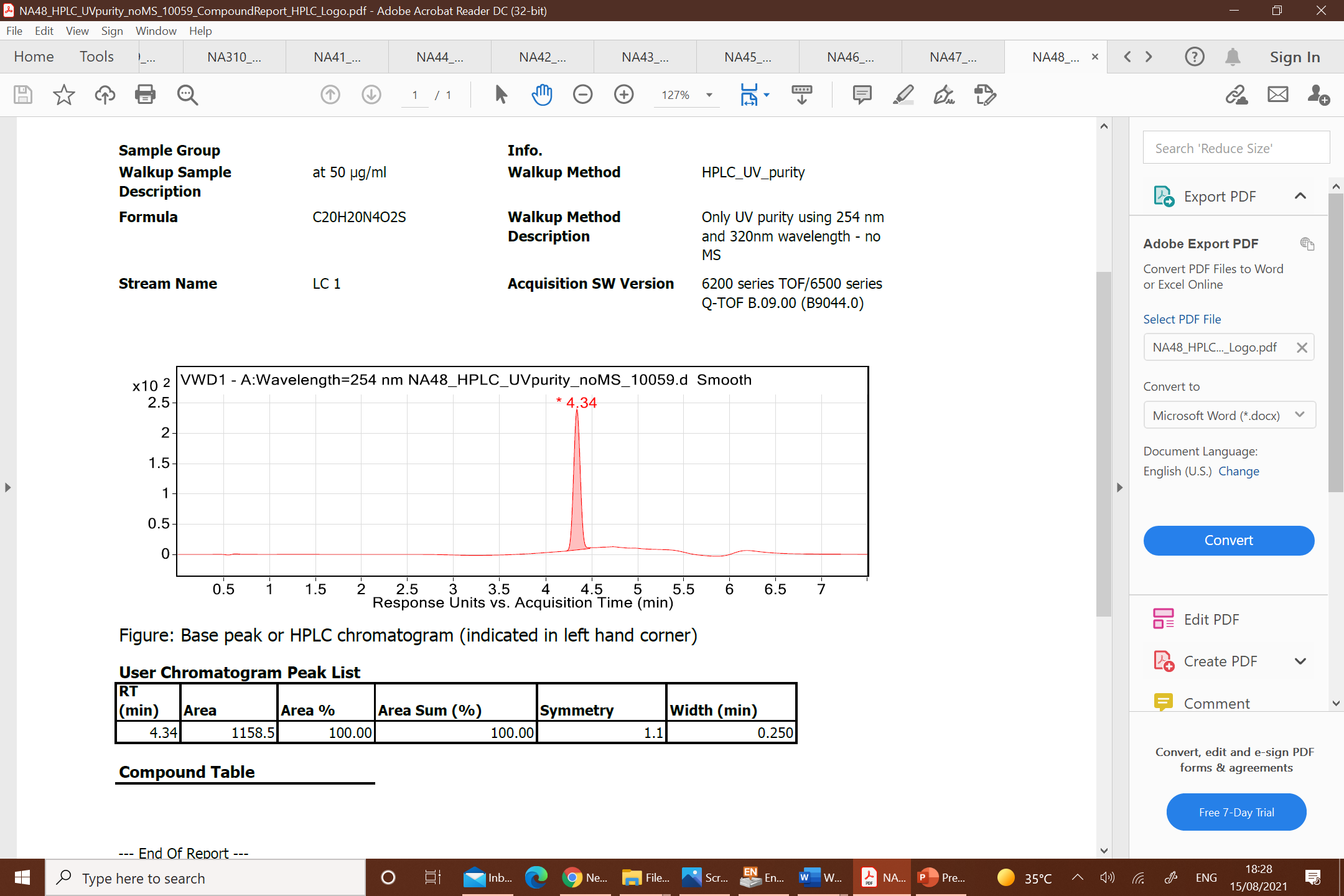


**9c**
